# The retinoblastoma protein regulates hypoxia-inducible genetic programs, tumor cell invasiveness and neuroendocrine differentiation in prostate cancer cells

**DOI:** 10.18632/oncotarget.8301

**Published:** 2016-03-23

**Authors:** Mark P. Labrecque, Mandeep K. Takhar, Rebecca Nason, Stephanie Santacruz, Kevin J. Tam, Shabnam Massah, Anne Haegert, Robert H. Bell, Manuel Altamirano-Dimas, Colin C. Collins, Frank J.S. Lee, Gratien G. Prefontaine, Michael E. Cox, Timothy V. Beischlag

**Affiliations:** ^1^ The Faculty of Health Sciences, Simon Fraser University, Burnaby, British Columbia, Canada; ^2^ Department of Urologic Sciences, The Vancouver Prostate Centre, University of British Columbia, Vancouver, British Columbia, Canada

**Keywords:** hypoxia, retinoblastoma protein, invasion, neuroendocrine differentiation, prostate cancer

## Abstract

Loss of tumor suppressor proteins, such as the retinoblastoma protein (Rb), results in tumor progression and metastasis. Metastasis is facilitated by low oxygen availability within the tumor that is detected by hypoxia inducible factors (HIFs). The HIF1 complex, HIF1α and dimerization partner the aryl hydrocarbon receptor nuclear translocator (ARNT), is the master regulator of the hypoxic response. Previously, we demonstrated that Rb represses the transcriptional response to hypoxia by virtue of its association with HIF1. In this report, we further characterized the role Rb plays in mediating hypoxia-regulated genetic programs by stably ablating Rb expression with retrovirally-introduced short hairpin RNA in LNCaP and 22Rv1 human prostate cancer cells. DNA microarray analysis revealed that loss of Rb in conjunction with hypoxia leads to aberrant expression of hypoxia-regulated genetic programs that increase cell invasion and promote neuroendocrine differentiation. For the first time, we have established a direct link between hypoxic tumor environments, Rb inactivation and progression to late stage metastatic neuroendocrine prostate cancer. Understanding the molecular pathways responsible for progression of benign prostate tumors to metastasized and lethal forms will aid in the development of more effective prostate cancer therapies.

## INTRODUCTION

A characteristic of many solid tumors is that they contain regions of low oxygen availability (hypoxia) and express elevated levels of hypoxia inducible factors (HIFs) [1]. The HIF1 complex, HIF1α and dimerization partner the aryl hydrocarbon receptor nuclear translocator (ARNT/HIF1β), is the master regulator of the hypoxic response. During hypoxia, HIFs accumulate, translocate to the nucleus, and bind ARNT [2]. The HIF1 complex then binds to hypoxia response elements and recruits co-activators such as the thyroid hormone receptor/retinoblastoma-interacting protein 230 (TRIP230) [3], CBP/p300 [4] and Brm/Brg-1 [5] to modulate the expression of genes. Typical HIF1-regulated genes include angiogenic and metabolic targets, such as vascular endothelial growth factor (VEGF) [6] and GLUT1 [7] but also include metastatic markers, like CXCR4 [8] and procollagen-lysine 2-oxoglutarate 5-dioxygenase 2 (PLOD2) [9]. Thus, the microenvironment of solid tumors is conducive to the activation of hypoxia-regulated genetic programs and these support tumor growth.

Rb is a tumor suppressor protein with a well characterized and canonical function as a cell cycle regulator by repression of E2F-mediated transcriptional activity [10]. Hypo-phosphorylated Rb binds E2F and prevents transcription of E2F-dependent mitotic and cell cycle programs. Thus, loss of Rb expression or function is a crucial step preceding tumor development [10, 11]. However, we recently demonstrated that TRIP230 and Rb form a complex with HIF1 and that hyper-phosphorylated Rb represses the function of TRIP230 and the transcriptional response to hypoxia [12]. Additionally, loss of Rb combined with hypoxia led to exacerbated HIF1-mediated transcriptional responses and concomitant increases in target protein expression and invasion in MCF7 breast cancer cells [12]. Interestingly, loss of Rb function is also associated with progression of several other cancers, including brain [13], lung [14] and prostate [15–17].

In prostate cancer, Rb-loss occurs in 25–50% of cases [15, 18]. Despite the high frequency of Rb inactivation, few studies have addressed the impact of this on the cellular response to hypoxia. In this study, we examined the consequences of Rb-loss and hypoxia in two different prostate cancer cell lines, 22Rv1 and LNCaP. Using short-hairpin RNA in LNCaP cells to knockdown Rb expression in concert with DNA micro-array technology, we found that Rb-loss deregulates the expression of hypoxia-mediated transcriptional programs that govern angiogenesis, metastasis and neuroendocrine differentiation (NED). Ultimately, this leads to acquisition of a more invasive phenotype and expression of bona fide NED protein markers in human prostate cancer cells.

## RESULTS

### Loss of Rb leads to deregulation of hypoxia-regulated genes and hypoxia-dependent acquisition of an invasive phenotype

**Figure 1:**
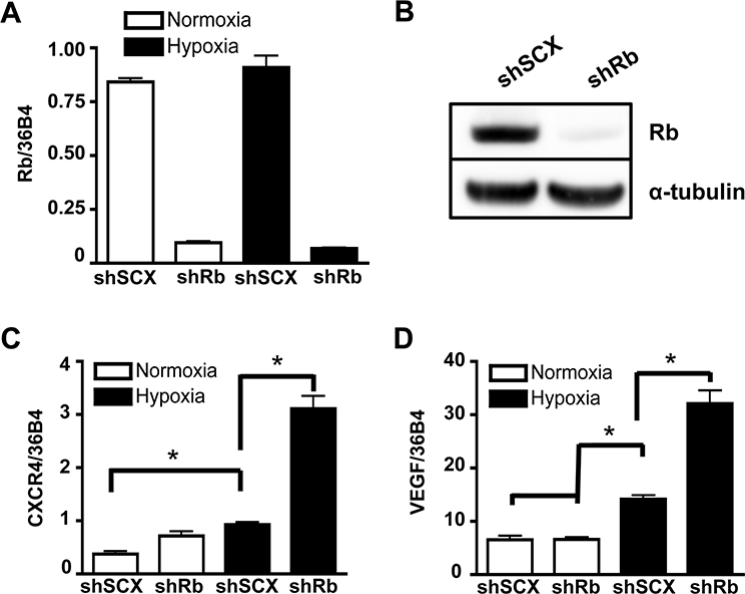
Ablation of Rb leads to transcriptional dysregulation of HIF1-target genes involved in metastasis and angiogenesis (**A**) LNCaP cells stably infected with either a shRNA to Rb (shRb) or a scrambled negative control shRNA (shSCX) were maintained in normoxic conditions or at 1% O_2_ for 24 h then gene expression was determined by quantitative real-time PCR after isolation and reverse transcription of total RNA. Rb expression was normalized to the constitutively active 36B4 gene expression. (**B**) Immunoblot analysis of whole cell extracts from shSCX and shRb LNCaP cells using anti-Rb or anti-α-tubulin primary antibodies. α-tubulin is the loading control. (**C**) CXCR4 and (**D**) VEGF mRNA accumulation was determined by RT-PCR after shSCX and shRb LNCaP cells were treated as described in (A). Open bars represent normoxia (20% O_2_) and closed black bars represent hypoxia (1% O_2_). Error bars represent ± S.D. and statistical significance was determined using a one-way ANOVA (*p < 0.05).

Previously, we demonstrated that ARNT, TRIP230 and Rb form a complex and that Rb represses the function of TRIP230 and the transcriptional response to hypoxia [12]. To more clearly define the role of Rb in hypoxia-mediated signaling, we used a retroviral vector expressing a short-hairpin (sh) RNA directed to Rb to permanently knockdown Rb expression in LNCaP prostate cancer cells. Stably infected LNCaP cells had either wild type Rb expression from a scrambled negative control vector (shSCX) or a stably ablated Rb protein (shRb) profile. Total Rb mRNA and protein levels were significantly attenuated in the LNCaP-shRb cells during both normoxia and hypoxia (Figure 1A and 1B). In addition, VEGF and CXCR4 mRNA accumulation in LNCaP-shSCX cells displayed typical hypoxia induction profiles, however, significantly exacerbated transcriptional responses occurred in LNCaP-shRb cells subjected to 24 hours of hypoxia when compared to scrambled controls (Figure 1C and 1D). Taken together, this data supports our notion that loss of Rb leads to dysregulation of hypoxia-inducible transcriptional processes in prostate cancer and reinforces the shRNA LNCaP lines as appropriate models to study this paradigm.

**Figure 2:**
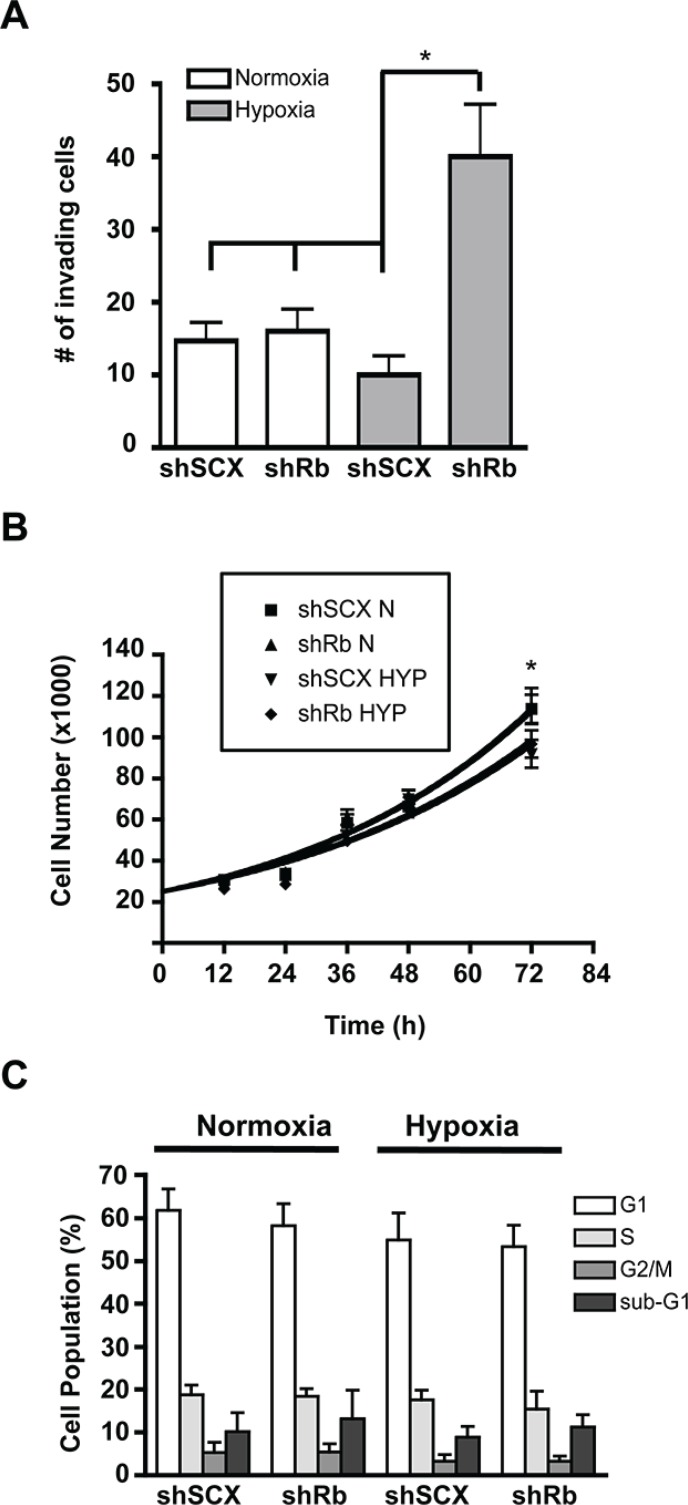
Hypoxia-inducible increase in invasion but not cell cycle or proliferation in LNCaP prostate cancer cells lacking Rb (**A**) shRNA LNCaP cells (1 × 10^4^) were seeded in Matrigel invasion chambers and then maintained in normoxic conditions or at 1% O_2_ for 36 h. Chambers were then prepared according to manufacturers protocols and cells were counted under a microscope. Assays were performed in triplicate. Error bars represent ± S.D. and statistical significance was determined using a one-way ANOVA (*p < 0.05). (**B**) Knockdown of Rb in LNCaP cells does not alter cell proliferation in response to hypoxia. Cells were either left at normoxia or treated with 1% O_2_ and cells were counted at 0, 12, 24, 36, 48, and 72 h later. Error bars represent ± S.E.M. and statistical significance was determined using a one-way ANOVA (*p < 0.05). (**C**) Knock-down of Rb in LNCaP cells does not alter cell cycle in response to hypoxia. Cell cycle status was determined by propidium iodide (PI) staining and flow cytometry. LNCaP cells with a scrambled negative control or with Rb ablated, were treated with hypoxia or left at normoxic conditions for 36-hours. The percentage of cells in each stage of the cell cycle was determined using FlowJo analysis software based on the PI staining profile of FSC/SSC-gated population. Assay was performed three times and each sample was read in triplicate. Error bars represent ± S.E.M.

Exacerbated expression of the metastatic marker CXCR4 with Rb-loss and hypoxia led us to hypothesize that LNCaP cells lacking Rb may acquire a more invasive phenotype compared to control cells. In order to determine this, we used Matrigel invasion chambers in concert with 36 hours of hypoxia or normoxia and shRb or shSCX LNCaP cells to test cell-line specific invasive potentials. A significant increase in invasion occurred only in cells depleted of Rb that had been exposed to hypoxia (Figure 2A). Next, we monitored cell growth over a 72-hour period to ascertain if increased growth characteristics contributed to the observed increase in invasion. Indeed, loss of Rb alone did not affect proliferation rates when compared to scrambled controls (Figure 2B). However, proliferation was significantly inhibited in both shSCX and shRb cells after 72-hours of hypoxia (*p* < 0.05) supporting the findings of others [19, 20]. Furthermore, subjecting shRNA LNCaP cells to hypoxia and then FACS sorting after propidium iodide staining revealed no significant differences between treatments at any stage of the cell cycle [G1, G2, S or sub-G1] (Figure 2C). Hence, these data strongly suggest that loss of Rb in LNCaP cells promotes cell invasion in a hypoxia-dependent fashion and that this effect is not due to increased cell growth or proliferation.

### Rb regulates specific hypoxia-regulated genetic programs

With the shRNA cell lines validated, we next used Agilent Genome-Wide human expression arrays and shRNA LNCaP cells either left at normoxia or treated with 1% O_2_ to delineate the role of Rb in hypoxia-regulated transcriptional programs. We narrowed our scope to focus only on genes whose expression was further exaggerated by loss of Rb in a hypoxia-dependent fashion as these are the genes that are most likely regulated by the HIF1-Rb complex. Thus, we selected genes from the shRb-hypoxia-treated data set that were up- or down-regulated significantly (*p* < 0.05) at least 2.0 fold when compared to the other treatments.

**Figure 3:**
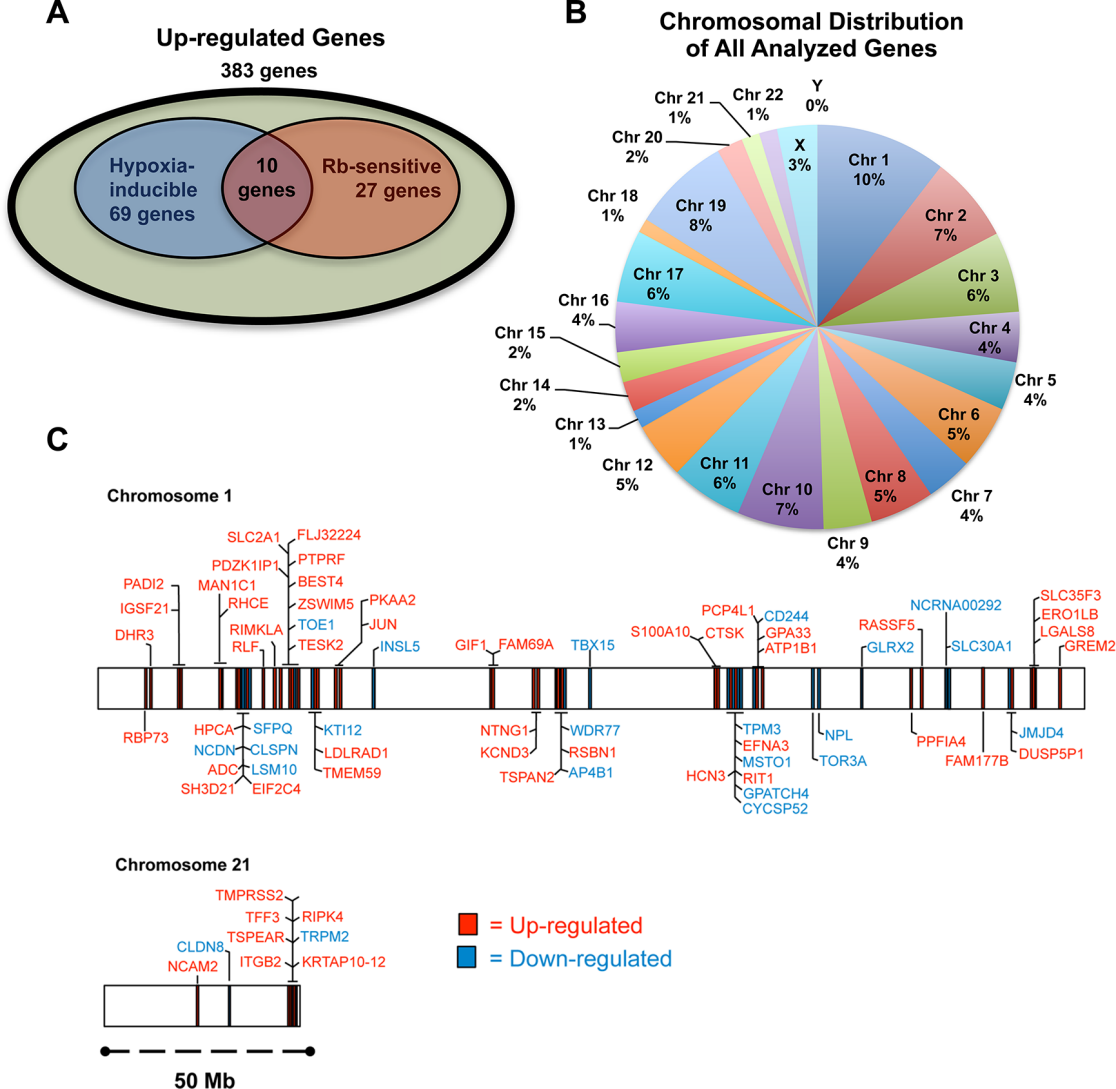
The role of Rb in hypoxia-regulated transcription LNCaP cells infected with either a short-hairpin control RNA (shSCX) or a short-hairpin to Rb (shRb) were maintained under hypoxic (1% O_2_) or normoxic (20% O_2_) conditions for 24 h. Extracted RNA was subjected to microarray analysis. Experiments were performed in triplicate and only genes that were up- or down-regulated at least 2-fold under hypoxic conditions with a *p*-value < 0.05 were considered significant. (**A**) Venn diagram of up-regulated genes, showing the overlap between hypoxia inducible genes, genes sensitive to loss of Rb and up-regulated genes sensitive to Rb-loss and hypoxia in combination. Olive shaded area represents genes that are from the shRb-hypoxia-treated data set that were up-regulated significantly (*p* < 0.05) at least 2.0 fold when compared to the other treatments. Blue shaded area represents genes that are hypoxia-inducible. Red shaded area represents genes that are up-regulated by Rb-loss. (**B**) A pie chart was used to illustrate the percentage of genes on each chromosome that were up- or down-regulated in Rb knockdown LNCaP cells. (**C**) Genes regulated by the Rb-HIF1 complex are in close proximity to one another at certain loci on select chromosomes. Chromosome maps of all the up- and down-regulated genes on chromosomes 1 and 21 according to their annotated start and stop sites are represented to scale.

For all up-regulated genes (Hyp-Rb vs. all other conditions; > 2-fold increase), micro-array analysis revealed that there are 383 genes that are significantly up-regulated by loss of Rb and hypoxia (Supplementary Data File 1, Table S1). In addition, of the 383 up-regulated genes, 69 are hypoxia inducible (Hyp-SCX vs Norm-SCX; > 2-fold increase), 27 are sensitive to loss of Rb (Norm-Rb vs Norm-SCX; > 2-fold increase) and 10 genes are both hypoxia inducible and sensitive to Rb-loss. Thus, 297 up-regulated genes are not sensitive to either loss of Rb or hypoxia alone but have exacerbated transcriptional responses with Rb-loss and hypoxia in combination (Figure 3A). We realize that due to our arbitrary 2-fold increase cut-off, many of these 297 genes are likely regulated by hypoxia or are sensitive to Rb-loss but these changes are ignored in this analysis. Nevertheless, the analysis narrowed our focus so that only the most sensitive targets were highlighted for subsequent investigation, as these targets are likely the true effectors of cancer cell transformation via the Rb-HIF1 transcriptional complex. The complete dataset from the arrays can be viewed at (http://www.ncbi.nlm.nih.gov/geo/query/acc.cgi?acc%20=%20GSE78245).

Evidence in the literature suggests that Rb may mediate transcriptional repression by recruiting chromatin modifiers to manipulate chromatin structure [12, 21–24]. Furthermore, knockdown or loss of epigenetic regulators can affect distinct chromosomes or modulate specific gene clusters [25–27]. Thus, we were interested to determine if Rb attenuates hypoxia-regulated transcription in the same fashion. A pie chart of all the up- and down-regulated genes and their associated chromosome is presented in Figure 3B. A Fisher's exact test demonstrated that the total number of genes on each chromosome from our list is not significantly different from the normal distribution of genes in the genome on each chromosome. However, we used the BioMart program and Ensemble Genes 70 and *Homo sapiens* genes (GRCh37.p10) databases to arrange significantly up- and down-regulated genes on chromosomes according to their annotated start and stop base pairs. When genes were mapped in this fashion, for example on chromosomes 1 and 21, a clustering pattern was observed (Figure 3C) for a large number of genes. Graphical representation of all genes and associated chromosomal start and stop locations are presented in the Supplementary Data File 2. To test if these distributions are different the distance between genes was calculated for genes on chromosome 1 and 21. This was done once for the genes of interest and then for 65 randomly chosen genes from chromosome 1 and 21. For these genes the median distance was calculated. The random choosing was repeated 100 times to generate a distribution of random medians. For both chromosome 1 and 21, the difference between the distribution of genes on our list and the randomly chosen genes was significantly different (*p*-value = 2.156e-05 and *p*-value < 2.2e-16, respectively). This pattern was observed on many other chromosomes (Supplementary Data File 2) suggesting that many of the Rb-sensitive hypoxia-regulated genes are in close proximity to one another (over several million basepairs). Taken together, our data suggest that the Rb regulates well-ordered hypoxia-inducible genetic programs. In addition, loss of Rb function permits exaggerated expression of genes within specific genomic regions and this may facilitate prostate cancer progression.

### Loss of Rb dysregulates hypoxia-mediated metastatic and neuroendocrine transcriptional programs in human prostate cancer cells

We identified a cohort of genes whose hypoxia-inducible transcriptional activity is either bolstered or activated by loss of Rb (383 genes). Conversely, we have also identified a cohort of down-regulated genes whose transcription is further repressed by Rb-loss and hypoxia (155 genes, Supplementary Data File 1, Table S2). Surprisingly, of the top 25 genes identified whose transcription is enhanced after Rb-loss under hypoxic conditions, 7 are neuronal markers or are associated with neuroendocrine differentiation (NED), including HTR5A [28], RORA [29], KISS1R [30], ALDOC [31], and ENO2 (a clinical hallmark of NED) [32]. Furthermore, 7 genes in the top 25 are associated with metastasis and/or angiogenesis, such as CXCR4, ANGPTL4 [33], PLOD2 [34], NDRG1 [35] and STC1 [36]. Finally, 15 of the top 25 up-regulated genes are directly regulated by either HIF1α or HIF2α or are known to be hypoxia-inducible (Table 1) [9, 37–48]. For the 10 remaining up-regulated genes, we performed an *in silico* consensus ARNT:HIF1α binding site analysis using reported DNA sequences and the JASPAR database [49, 50]. The analysis determined that all 10 genes contained multiple consensus sequences for HIF1 binding sites that may bind the ARNT:HIF1α transcriptional complex (Supplementary Data File 1). This data supports our previous findings that Rb regulates the HIF1 transcriptional complex [12] and that Rb-loss under hypoxic stress leads to aberrant expression of HIF1 target genes involved in metastasis and NED.

**Table 1:** The top 25 up-regulated genes that are induced greater than 2-fold by a combination of loss of Rb and hypoxia when compared to negative controls

Gene Name	Probe Name	Fold Induction (vs shSCX-N)			Hypoxia Inducible/HIF1-regulated
		shRb-N	shSCX-HYP	shRb-HYP	
HTR5A	A_23_P42565	2.07	27.41	**256.40**	[44]
PLOD2	A_33_P3318581	2.53	24.26	**219.02**	[9, 42, 44]
SLC16A3	A_23_P158725	1.73	24.85	**200.17**	[45]
ATP4A	A_23_P430728	2.46	12.53	**157.93**	*
PLA2G4D	A_33_P3361611	1.15	10.05	**97.72**	*
NIM1	A_23_P254863	1.32	5.73	**91.57**	*
CYP26A1	A_23_P138655	1.15	2.87	**68.51**	*
CXCR4	A_23_P102000	1.30	2.65	**62.56**	[43]
KISS1R	A_33_P3231357	0.61	8.51	**61.94**	*
ANGPTL4	A_33_P3295358	1.92	3.63	**53.17**	[39, 44]
GPR26	A_23_P305581	0.98	9.84	**53.00**	*
MYBPC2	A_33_P3257182	1.18	3.74	**50.39**	*
FOS	A_23_P106194	1.47	1.41	**49.01**	[44]
PPFIA4	A_23_P420692	1.61	8.32	**39.76**	[47]
CA9	A_23_P157793	1.18	2.09	**28.63**	[42, 44, 48]
NFATC4	A_33_P3250083	1.52	4.70	**21.97**	[42]
PFKFB4	A_24_P362904	1.53	2.94	**21.82**	[41, 42, 44]
PCP4L1	A_32_P214665	1.97	2.51	**21.02**	*
RORA	A_23_P26124	1.40	2.57	**20.81**	[40]
AMPD3	A_24_P304154	0.91	0.89	**19.43**	*
ALDOC	A_23_P78108	1.46	4.85	**19.33**	[42, 44]
ENO2	A_24_P236091	1.80	2.61	**19.09**	[42]
SCNN1G	A_23_P206626	1.10	1.30	**19.01**	*
STC1	A_23_P314755	1.10	4.42	**19.00**	[38]
NDRG1	A_23_P20494	1.19	6.80	**18.60**	[37, 46]

* The (*) denotes genes containing putative HREs identified in the *in silico* consensus ARNT:HIF1α binding sequence analysis (Supplementary Data File 1).

**Figure 4:**
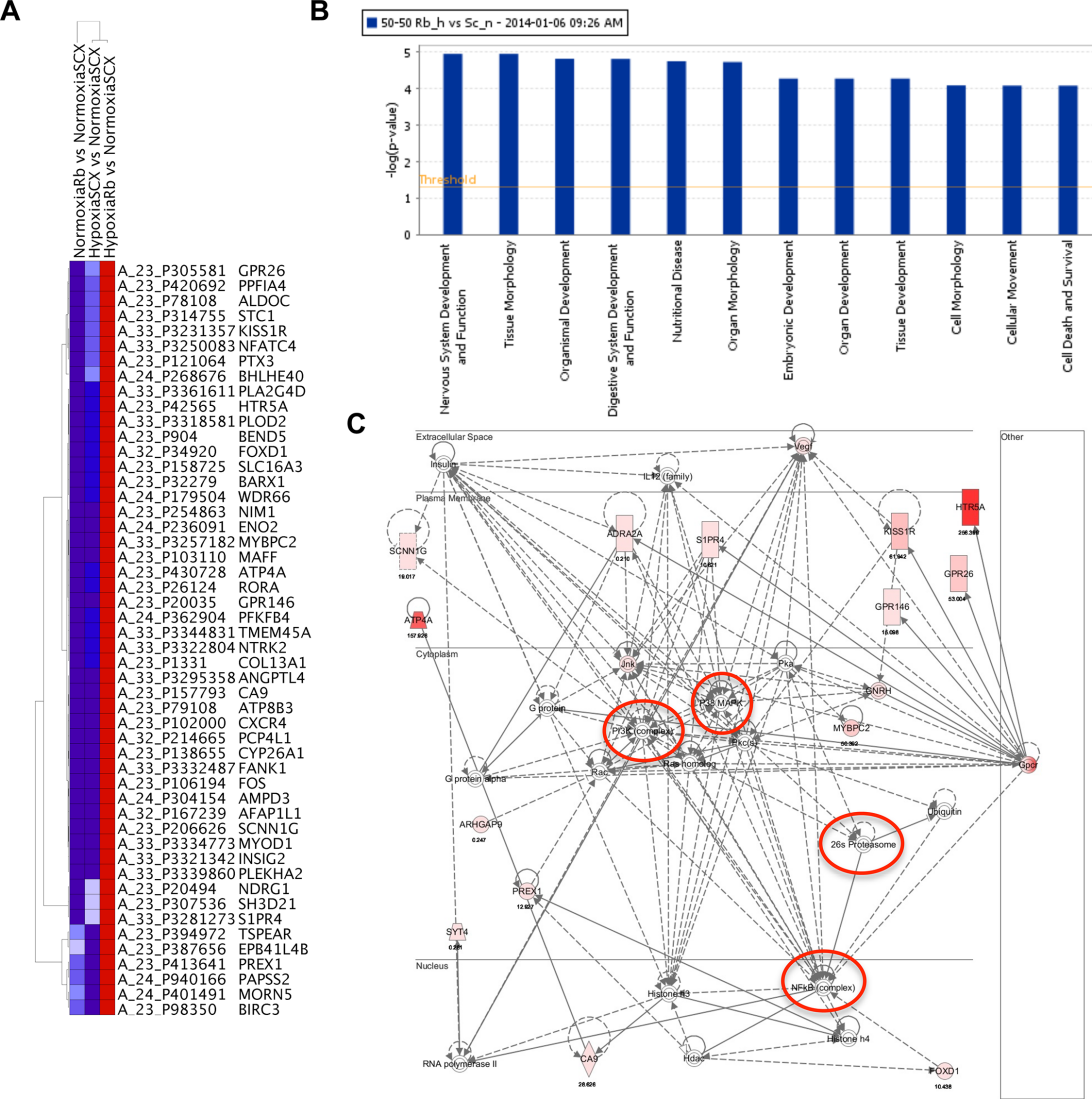
Rb-loss leads to transcriptional dysregulation of hypoxia-regulated genes involved in metastasis, angiogenesis and neuroendocrine differentiation (**A**) Heat map of the 50 genes displaying the largest increase in fold gene expression in response to hypoxia after knock-down of Rb. (**B**) The top systems and diseases identified by Ingenuity Pathway Assist (IPA) analysis. The top 12 systems and diseases associated with the 50 most up-regulated and 50 most down-regulated hypoxia sensitive genes after knock-down of Rb. The –log of the *p*-value is represented on the ordinate. (**C**) Nervous system development and function network identified by IPA analysis. Nodes identified are indicated with red circles. Up-regulated genes identified in our micro-array screen are filled with varying shades of pink and red with the most highly expressing genes shaded red and the lower expressing genes shaded pink.

Hierarchical clustering was performed using Ingenuity Pathway Assist (IPA) on the top 50 genes whose expression was most up-regulated with Rb-loss and hypoxia (Figure 4A). Interestingly, the top two diseases associated with the 50 genes are cancer (32 molecules) and neurological disease (17 molecules). Moreover, the top two cellular and molecular functions associated with these genes are cell death and survival (10 molecules) and cellular movement (17 molecules). Importantly, gene ontology analysis revealed that the top two associated transcription factors were HIF1α (*p*-value of overlap = 2.49E-15) and EPAS/HIF2α(*p*-value of overlap = 2.30E-14), thus strengthening our hypothesis that this phenomenon is mediated via a HIF-regulated mechanism and not an idiosyncratic effect of the short hairpin RNA. The summary of the IPA analysis can be found in Supplementary Data File 3. Taken together, this analysis indicates that the top 50 up-regulated genes support prostate cancer initiation or progression through up-regulation of pro-metastatic and neuroendocrine programs.

**Figure 5:**
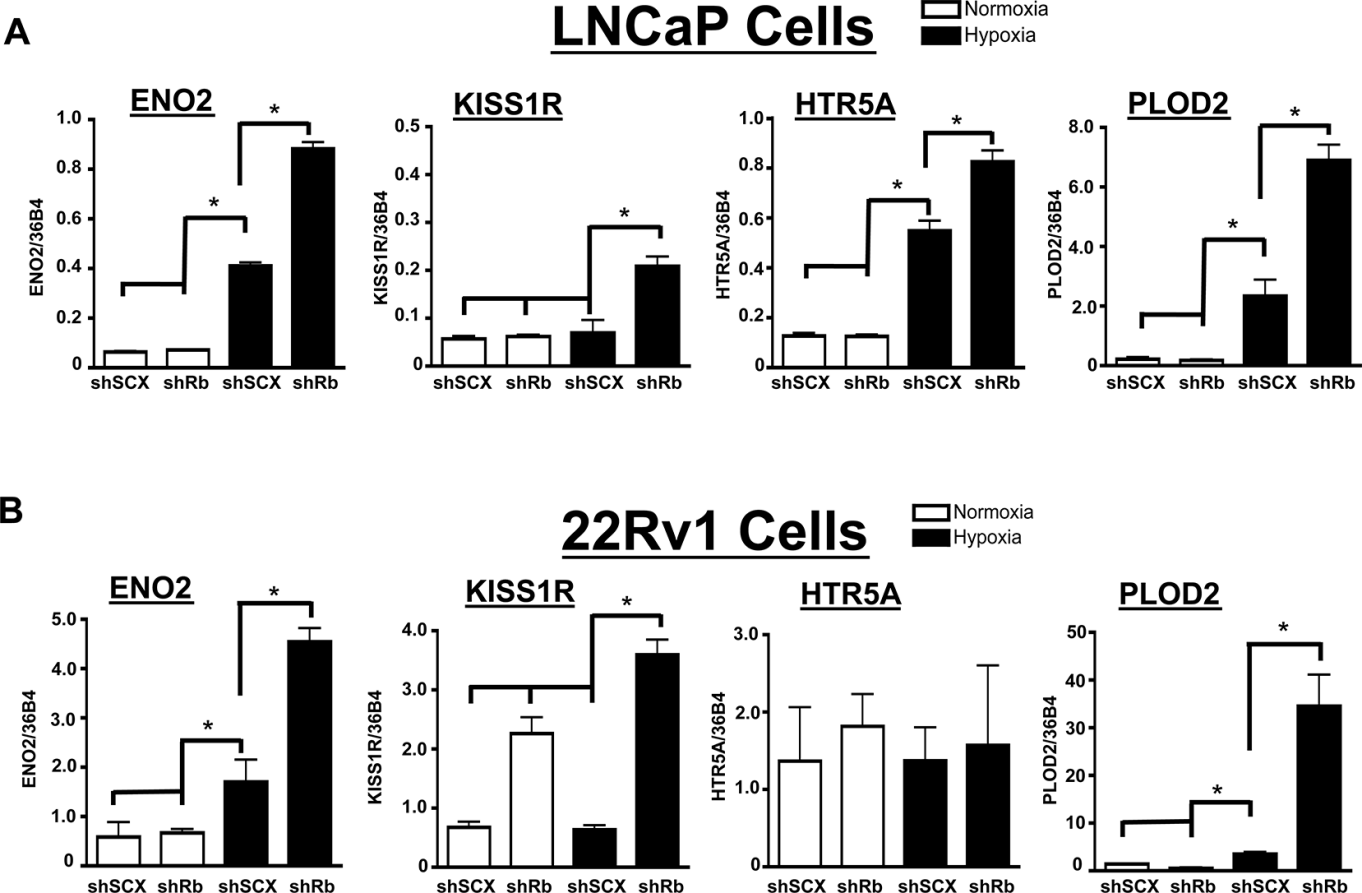
Confirmation of Rb-sensitive and hypoxia-inducible neuroendocrine targets identified through microarray analysis (**A**) LNCaP or (**B**) 22Rv1 cells infected with either a short-hairpin control RNA (shSCX) or a short-hairpin to Rb (shRb) were maintained under hypoxic (1% O_2_) or normoxic (20% O_2_) conditions for 24 h. Extracted RNA was subjected to qRT-PCR. Transcriptional responses of ENO2, KISS1R, HTR5A and PLOD2 are displayed and expression was normalized to the constitutively active 36B4 gene expression. Open bars represent normoxia (20% O_2_) and closed black bars represent hypoxia (1% O_2_). Error bars represent ± S.D. and statistical significance was determined using a one-way ANOVA (*p < 0.05).

Although the IPA analysis of the top 50 up-regulated genes provided valuable insight on hypoxia-inducible oncogenic targets, the true consequences of Rb-loss on hypoxia-regulated transcriptional programs were highlighted when the top 50 down-regulated targets were also included in the analysis. IPA analysis determined that the top physiological system development and function associated with these 100 genes are 1) nervous system development and function (21 molecules), 2) tissue morphology (24 molecules), 3) organismal development (32 molecules), 4) digestive system development (12 molecules) and 5) organ morphology (23 molecules). The top molecular functions are cell morphology (27 molecules) and cellular movement (24 molecules). For all physiological systems and molecular pathways involved, the –log *P* values are all greater than 4 and this suggests a highly significant relationship between the identified genes and associated pathways (Figure 4B). Finally, the top two associated network functions are (1) Cellular Movement, Hematological System Development and Function, Immune Cell Trafficking (Supplementary Data File 1, Figure S1) and (2) Neurological Disease, Psychology Disorders, Cardiovascular Disease (Figure 4C). The complete IPA analysis summary can be found in the Supplementary Data File 3 and all identified networks can be found in the Supplementary Data File 1 (Figures S1–S6). Analysis of the neurological disease network identified several key up-regulated targets such as HTR5A, KISS1R and GPR146. Moreover, the network analysis identified nodes in signaling cascades like PI3K, MAPK and NFκB as the ultimate downstream targets for disease progression. There were no significantly down-regulated genes identified in the neurological disease network however, the power of the analysis relies only on the known associations in the literature. We have confirmed the array data for the putative neuroendocrine markers ENO2, KISS1R, HTR5A and PLOD2 through qRT-PCR (Figure 5A). Likewise, the array confirmed PLOD2 and CXCR4 as bona fide targets of the Rb-HIF1 complex that we reported previously [12]. Importantly, knockdown of DP1 protein with siRNA did not significantly alter the hypoxia inducible accumulation of ENO2, KISS1R, HTR5A or PLOD2 in our shRb knockdown cells (Supplementary Data File 1, Figure S7). This strongly suggests that these genes are not E2F regulated.

**Figure 6:**
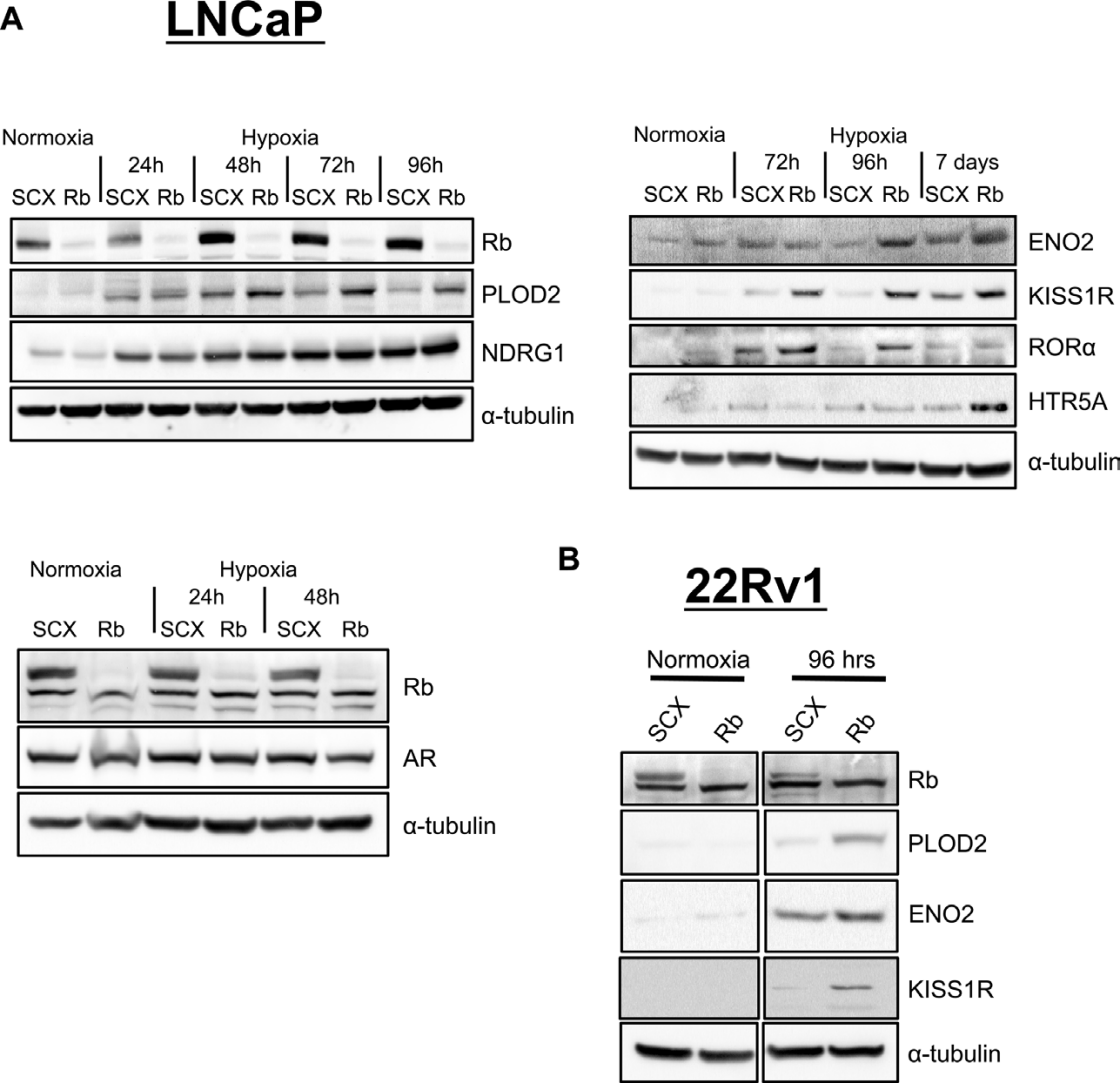
Loss of Rb results in increased expression of proteins involved in metastasis and neuroendocrine differentiation in prostate cancer cells in a hypoxia-dependent fashion (**A**) LNCaP and (**B**) 22Rv1 shRNA cells were exposed to normal O_2_ levels or 1% O_2_ for various times up to 7 days as indicated. Whole cell lysates were fractionated by SDS-PAGE and examined by immunoblotting using affinity purified antibodies to α–tubulin, Rb, AR, ENO2, KISS1R, RORα, PLOD2, NDRG1 and HTR5A.

LNCaP cells are classically defined as AR positive, hormone-responsive and metastatic prostate cancer cells [51]. Furthermore, LNCaP cells can transition to a neuroendocrine state through androgen deprived culturing methods [52]. Thus, we were interested in determining if the observed transcriptional responses could be recapitulated in another prostate cancer cell line that is already androgen-insensitive. We therefore retrovirally introduced the shSCX and shRb constructs into 22Rv1 prostate cancer cells and subjected transformed cells to normoxic or hypoxic conditions. The shSCX 22Rv1 cells expressed Rb protein while the shRb cells had significant Rb knockdown (Figure 6B). Notably, ENO2, KISS1R and PLOD2 transcriptional levels in the shRNA 22Rv1 cells mirrored the LNCaP shRNA transcription profiles and were exacerbated under hypoxic conditions with loss of Rb (Figure 5B). Interestingly, HTR5A did not respond in the same fashion but this may be due to a cell-type specific response. Nevertheless, this data suggests that Rb modulates hypoxia-regulated gene programs in prostate cancer independent of clinical stage or cell-type.

### Rb-loss results in a hypoxia-dependent increase in expression of proteins involved in metastasis and neuroendocrine differentiation in human prostate cancer cells

To further validate the array, we used the shRNA expressing LNCaP and 22Rv1 cells and immunoblotting to measure the level of protein expression for identified genes. The shRNA cell lines were exposed to normal O_2_ levels or 1% O_2_ for various times up to 7 days. Protein levels for the most highly expressing genes (HTR5A, KISS1R, PLOD2, ENO2, NDRG1 and RORα) imitated the transcriptional responses observed in the array (Figure 6A and 6B). In addition, shRb cells exposed to chronic hypoxia (2–7 days) exhibited a more exacerbated protein expression for the targets of interest than cells that were treated with acute hypoxia (24 hrs). This observation supports our hypothesis that the key effectors of cellular transformation and metastasis require both transcriptional and translational processes for exacerbated accumulation with Rb-loss and hypoxia. Additionally, these data support a role for the Rb-HIF1 complex in the maintenance of normal cell physiology and the loss of Rb leads to activation of hypoxia-regulated networks involved in cellular movement and transformation that drive prostate cancer cells to acquire metastatic and neuroendocrine phenotypes.

### KISS1R is linked to intracellular calcium mobilization in 22Rv1 cells

**Figure 7:**
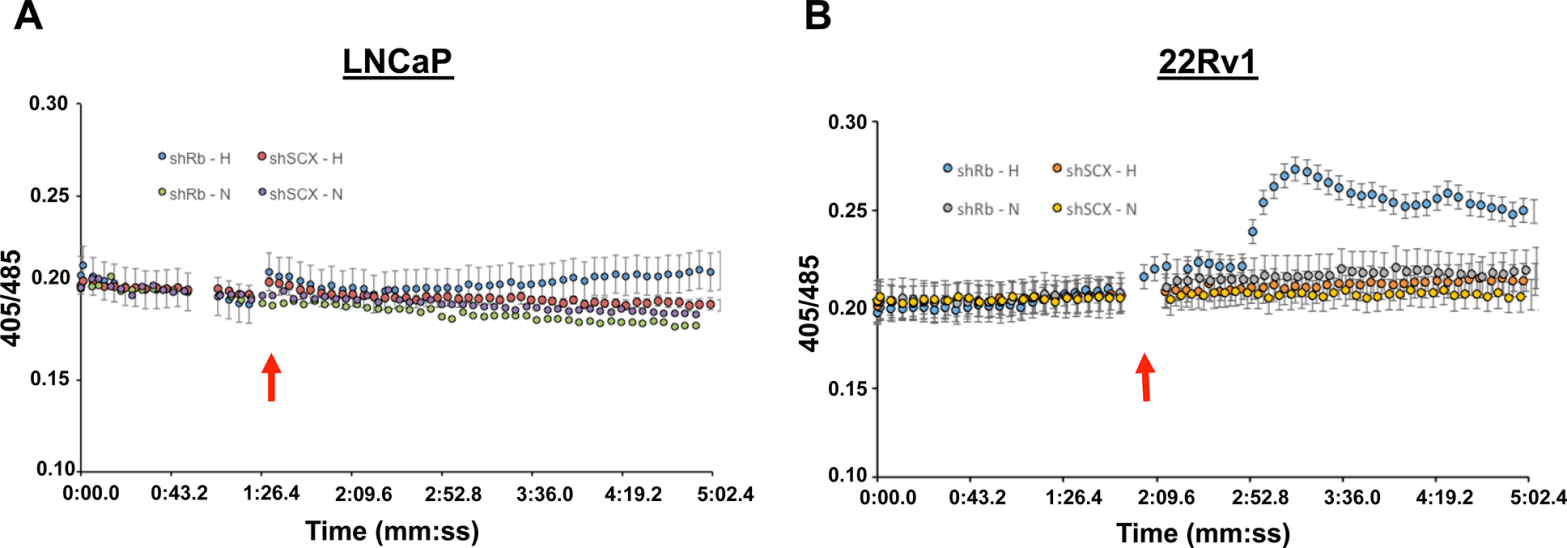
Kisspeptin-10 activates calcium signaling in 22Rv1 cells Application of 1 uM kisspeptin-10 peptide in (**A**) LNCaP or (**B**) 22rV1 cells lacking Rb (shRb) and subjected to 96 hours of hypoxia led to a significant increase in intracellular calcium levels compared to control scrambled (shSCX) hypoxia cells and to normoxia cells (both shRb and shSCX) in 22Rv1 cells only. After hypoxia or normoxia treatment, cells were loaded with 2 uM indo-1AM dye (30 min at 37°C) and then imaged on an inverted fluorescent microscope. Application of 1 uM kisspeptin-10 (indicated by red arrows) led to a significant increase intracellular calcium levels in 22Rv1 cells as indexed by ratiometric fluorescence measurements taken at 405 and 485 nm. A one-way ANOVA was performed followed by a post-hoc Tukey test. *p < 0.05, **p < 0.01 vs 1:20 time point, *n* = 5.

The significant increases in both mRNA and protein expression after Rb-loss and hypoxia for several of our identified array genes suggests that functional consequences specific to these genes may be present. Kisspeptin/KISS1R interactions and activation of the Gqα–p63RhoGEF signaling cascade has been identified as a driver of metastasis in breast cancer cells [53]. Additionally, activation of Gqα is canonically coupled to an increase in cellular calcium mobilization. Thus, to establish if KISS1R expression can influence sensitivity to kisspeptin in prostate cancer, we performed fluorescence based calcium mobilization assays in control (shSCX) and shRb LNCaP and 22Rv1 cells after normoxia or hypoxia treatments. Despite strong immunocytochemical staining of KISS1R in the cytoplasm and cell membrane in hypoxia treated shRb LNCaP cells (Supplementary Data File 1, Figure S8), 1 uM kisspeptin-10 failed to produce a detectable calcium mobilization response in LNCaP cells (Figure 7A). However, in shRb 22Rv1 cells subjected to 96 hours of hypoxia, application of 1 uM kisspeptin-10 peptide led to a significant increase in intracellular calcium levels compared to shSCX hypoxia cells and to shRb and shSCX normoxia cells (Figure 7B). Many possible explanations exist for why kisspeptin-10 failed to trigger intracellular calcium release in hypoxic shRb-LNCaP cells. Most GPCRs require proper folding and targeting to the membrane in order to function [54]. Indeed, many GPCRs exist in the membrane and are non-functional due to improper folding [55]. Another possible explanation is that LNCaP cells do not express the Gq subunit required for KISS1R coupling or that KISS1R is coupled to another signal transducer. Taken together, these data suggest that PCa cells that lose Rb in a hypoxic environment undergo dramatic molecular, biochemical, physiological and phenotypic changes. These adaptations enable them to better tolerate an oxygen-deprived environment and aid in the transformation to a more aggressive cancer phenotype.

## DISCUSSION

In healthy tissue, HIF proteins are tightly regulated and hypoxia via the HIF1 complex activates genetic programs regulating key physiological functions in a coordinated fashion [56]. However, chronic hypoxia in solid tumor microenvironments leads to activation of HIF1 transcriptional programs, microvascular hyperplasia and metastasis and this promotes tumor progression [57]. In addition, loss of Rb function or genetic ablation of *RB1* has been implicated in advanced stages of brain cancers [13, 58], lung cancer [59] and in 25–50% of late stage prostate cancer cases [15, 18]. As a result, we were interested in further investigating the physiological connection between Rb function and hypoxia-inducible gene expression, especially hypoxia-regulated transcriptional programs involved in cancer cell transformation. Previously, we demonstrated that Rb attenuates the HIF1-mediated physiological response to hypoxia and that it is an integral and indispensable part of the HIF1 transcriptional complex by virtue of a direct interaction with TRIP230 [3, 12]. The TRIP230 protein is a member of the coiled-coil co-activator family of proteins and was first isolated as a thyroid hormone-dependent co-activator of TR-mediated signaling [60]. In addition, Rb associates with TRIP230 through distinct interaction motifs and this association dampens TRIP230 co-activator potential [12, 60]. Subsequent studies have established a function for Rb independent from its role in E2F-mediated regulation of cell cycle by determining that only the hyper-phosphorylated form of Rb interacts with TRIP230 [12, 61]. Further characterization of TRIP230 revealed that it is also a part of the p160 co-activator complex [62] a bona fide transcriptional co-activator complex associated with ARNT during activated transcription [63]. Finally, TRIP230 interacts with ARNT as a co-activator in hypoxia signaling and HIF1-mediated transcriptional activity is abolished in cells depleted of TRIP230 [3, 64]. Thus, TRIP230 is a multifaceted co-activator protein that is required for the physiological response to hypoxia.

In this study, we examined the consequences of Rb-loss and hypoxia in LNCaP and 22Rv1 prostate cancer cells. Permanent Rb knockdown led to a concomitant dysregulation of hypoxia-inducible transcriptional activity and increased invasiveness in a hypoxia-dependent fashion (Figures 2 and 5). In addition, we used DNA microarray technology to identify novel gene targets regulated by the Rb-HIF1 complex and to determine how Rb-loss and hypoxia affect the genetic landscape in prostate cancer evolution. The micro-array analysis identified 383 up-regulated genes and 155 down-regulated genes sensitive to Rb-loss and hypoxia (Figure 3A). Interestingly, these genes highlight a function for Rb in the attenuation of hypoxia-regulated transcriptional programs that govern both metastasis and neuroendocrine differentiation (NED). Thus, loss of Rb expression or function in combination with hypoxia leads to up-regulation of bona fide and newly discovered neuroendocrine and metastatic markers at the protein level and highlights a hitherto unrecognized mode of cellular transformation in prostate cancer models. Clinically, loss of Rb implies either ablation of gene/protein or loss-of function. Loss-of-function of pRb while retaining protein immuno-reactivity is well documented in prostate cancer and other solid tumor types [65–67]. We mimicked loss of Rb using retroviral expression of short-hairpin RNAs to knock-down Rb in the LNCaP and 22Rv1 cells lines. Thus, attempts to correlate pRb levels with neuroendocrine markers of any other gene candidates culled from our experiments likely would be uninformative.

We recognize that a limitation to our analysis is that some bona fide hypoxia-sensitive and Rb-sensitive targets are shifted to the hypoxia-insensitive or Rb-insensitive classes due to the arbitrary 2-fold induction requirement. However, this analysis narrowed the focus to targets that are most sensitive to Rb-loss and hypoxia and elucidated four intriguing possibilities in our paradigm. Firstly, that many genes are directly hypoxia-inducible and contain hypoxia response elements (HREs) in their regulatory regions and that loss of Rb unmasks the full co-activation potential of TRIP230 during HIF1-mediated transcription. This permits exacerbated expression of pro-metastatic and neuroendocrine markers in solid tumor microenvironments that are not normally expressed or are expressed at low levels in oxygenated tissues or in cells expressing functional Rb. Secondly, there is the possibility that the TRIP230-Rb interaction exists with other transcription factors on the affected genes and these parallel pathways are also affected by Rb-loss. This likelihood should be the focus of future investigations. Thirdly, some of these genes may also be downstream effectors of primary HIF1/Rb target genes. However, we exposed cells to hypoxia for only 24 hours in an attempt to minimize noise from expression of downstream targets. Finally, Rb in the context of the HIF1α-ARNT-TRIP230 complex, may be involved in long-range genomic interactions and genes in close proximity to HIF1α-ARNT-TRIP230-regulated genes are significantly impacted by loss of Rb. Interestingly, the latter point is further supported by statistical analysis and chromosome maps for up- and down-regulated genes displaying base pair start and stop sites (Figure 3C). The analysis revealed that many of the Rb-sensitive hypoxia-regulated target genes are relatively close to each other on many chromosomes. This suggests that Rb may be important for maintaining proper chromatin structure. The ability for Rb to recruit chromatin modifying proteins to DNA-regulatory elements has been well established with confirmed interactions between SUV39H1 [22], HDACs [12, 23], DNMT1 [24] and SIN3a/b [12]. Moreover, 3-dimensional chromatin interactions such as chromatin looping, constitute a primary mechanism for regulating transcription in mammalian genomes and has been demonstrated for ERα-regulated transcriptional programs [26] and CTCF-mediated chromatin organization and transcriptional regulation [27]. It was previously determined that ~15% of HREs in the genome are in long-range relationship to genes (> 50 kb) and this suggests that a significant number of HIF1-regulated genes could be affected by long-range transcriptional activities [68]. Undoubtedly, more research is required to determine the exact mechanisms Rb employs to attenuate HIF1 transcription, however we speculate that the HIF1-TRIP230-Rb complex regulates some genetic programs through long-range chromatin interactions, such as chromatin looping, and Rb-loss leads to deregulation of these chromatin interactions.

One of the startling revelations of this research was the observation that certain clinical and prognostic markers of advanced stages of prostate cancer were manifested in hypoxic LNCaP and 22Rv1 cells after loss of Rb. The evolution of prostate cancer from initial diagnosis and treatment ultimately determines clinical outcome. Neuroendocrine prostate cancer (NEPC) is an androgen receptor-negative prostate cancer subtype that can occur sporadically but most commonly evolves from primary prostate adenocarcinoma [69]. In addition, NEPC has a poor clinical outcome and may represent ~25% of late stage prostate cancer [69, 70]. Recent molecular characterization of NEPC showed up-regulation of AURKA and MYCN expression and co-operative function to induce neuroendocrine differentiation in prostate cancer cells [71]. However, molecular determinants of NED remain understudied and poorly characterized. We have demonstrated that Rb-loss in conjunction with hypoxia leads to acquisition of a more invasive phenotype in LNCaP cells (Figure 2A) and expression of neuroendocrine markers in prostate cancer cells (Figure 6A and 6B). Microarray analysis using IPA identified a network of genes that may contribute to NED, namely HTR5A, KISS1R and ENO2. ENO2 (neuron-specific enolase, NSE) is a marker that is characteristically expressed in neuroendocrine prostate cancers and is used in clinical settings to determine prostate cancer progression [32]. Thus, the exacerbated expression of ENO2 in shRb cells exposed to hypoxia provides further support that Rb-loss in late stage prostate cancers permits transformation to a neuroendocrine state (Figure 6A and 6B).

A hallmark of NEPC is sensitivity to serotonin and other neuro-signaling molecules that support cell growth and proliferation [72]. The striking up-regulation of HTR5A mRNA and protein levels only in prostate cancer cells exposed to hypoxia and lacking Rb expression (Figures 4 and 5) leads to the possibility that NE cells may develop sensitivity to serotonin in this fashion. This is the first time HTR5A has been implicated in prostate cancer progression and development of HTR5A antagonists may prove to be a viable treatment option for men who develop neuroendocrine prostate cancer. Moreover, we identified exacerbated expression of KISS1R protein in both LNCaP-shRb and 22Rv1-shRb cells when exposed to hypoxia (Figure 5). The KISS1 molecule was originally identified as a metastasis inhibitor and potent activator of KISS1R (GPR54) [73]. However, the KISS1/KISS1R signaling cascade is also critical for controlling the gonadotropic axis by stimulating GnRH release from neurons in the hypothalamus [74]. Chronic exposure to KISS1 analogs desensitizes KISS1R and attenuates GnRH release leading to decreased plasma testosterone levels [75, 76]. Hence, the therapeutic potential of KISS1R agonists in treating men with prostate cancer has recently been investigated. Phase 1 trials for TAK-448 revealed that it is an inhibitor of the hypothalamic-pituitary-gonadal axis and that sustained exposure potently decreased testosterone levels and prostate-specific antigen through KISS1R desensitization [77]. However, therapeutic programs using KISS1 agonists to modulate KISS1R-expressing neurons likely activate KISS1/KISS1R signaling in individual cancer cells. KISS1R expression has been identified as a driver of metastasis in breast cancer cells [53]. Activation of Gqα–p63RhoGEF signaling cascade through autocrine KISS1/KISS1R signaling [53] and transactivation of EGFR through direct association with KISS1R [78] are thought to be the key pathways involved in KISS1R-mediated metastasis. Interestingly, our microarray analysis determined that shRb LNCaP cells exposed to hypoxia led to a 7.56-fold increase in EGFR expression (Supplementary Data File 1, Table S1). Additionally, our data suggests that KISS1R may be linked to Gq to initiate intracellular calcium mobilization in at least some compromised prostate cancer cells (Figure 7B). This may have implications for the therapeutic use of kisspeptin agonists and antagonists in the treatment of metastatic castration resistance prostate cancer (mCRPC), however the clinical significance and relationship between EGFR up-regulation and pro-metastatic KISS1R-signaling remains to be determined. Our observed increases in KISS1R expression in both LNCaP and 22Rv1 cells after Rb-loss and hypoxia were surprising as a previous report suggested that KISS1 inhibits metastasis and that both KISS1 and KISS1R protein levels appear to decrease with prostate cancer progression [79]. However, Wang and colleagues suggest that the anti-metastatic activity of KISS1 is likely elicited through pathways independent of KISS1R [79]. Furthermore, our data suggests that KISS1R expression only occurs in hypoxic tumor environments after Rb-loss and these parameters were not determined for the tissue specimens in the previous report.

The identification of PI3K, MAPK and NFκB through IPA analysis as central nodes of the Rb-dependent hypoxia signal in the neurological disease network leads to the intriguing possibilities of new drug targets for late stage prostate cancer (Figure 4C). The NFκB system mediates the inflammatory response and is associated with the invasive cancer cell phenotype [80]. Nuclear localization and activation of NFκB targets is also associated with metastasis and prostate cancer progression [81, 82]. Seventeen of the highest expressing genes in this network were identified in our screen as being hypoxia-inducible and sensitive to loss of Rb. Nine of these seventeen genes transduce signals to modulate NFκB activity. This suggests a role for Rb-loss and hypoxia in NFκB signaling and that activation of the NFκB pathway in prostate cancer may signal a progression to metastatic, castrate-resistant or neuroendocrine disease. Likewise, the PI3K pathway is crucial for LNCaP survival in androgen deprived environments and PI3K inhibition stabilizes p27^kip1^ expression [83]. Lastly, eight of the 17 genes in this network that were identified in our screen directly activate the ERK1/2 pathway, which was identified as a major node of this network and is a mediator of neoplastic cell invasion and transformation [84]. The over expression of several Rb-sensitive hypoxia-inducible factors promote invasion including CXCR4 [85], ANGPTL4 [33], PLOD2 [34], NDRG1 [35] and STC1 [36]. The identified nodes pose an avenue of further research to determine if prostate cancer cell transformation can be blocked through inhibitors of the MAPK signaling cascade or if targeting the individual up-regulated factors with chemotherapeutic agents can effectively combat neuroendocrine cancers.

Castration Resistant Prostate Cancer (CRPC) is a common progression after surgical resection or hormone deprivation therapy for initial prostate tumors. The CRPC stage is characteristically hormone refractory (androgen-insensitive) due to constitutively active AR activity or selection of cells that bypass requirements for AR-mediated growth and proliferation [86]. Furthermore, transition to metastatic CRPC is associated with a poor prognosis and treatment options become limited and rarely curative [86]. We determined that permanently ablated Rb expression in the 22Rv1 cell line leads to hypoxia-mediated increases in pro-metastatic genes (Figure 6B). This suggests that loss of Rb in CRPC contributes to the progression and lethality of the disease and that cellular transformation through the Rb-TRIP230-HIF1 complex is not just a function of hormone-sensitive or early stage prostate cancers. A previous study determined that Rb-loss in LNCaP cells is sufficient for progression to CRPC and that this was due to E2F-mediated up-regulation of androgen receptor (AR) signaling [17]. However, the oxygen status of these tumor xenografts is unknown. Nevertheless our array data revealed that AR transcriptional levels were not affected by hypoxia or Rb-loss nor were protein levels after 24 and 48 hours (Figure 6A). In addition, observations by several other groups support our working paradigm in prostate cancer. Firstly, HIF1 expression may be required for CRPC progression [87]. Second, previous *in vivo* experiments support a tumor suppressor role for E2F-binding deficient Rb in prostate cancer [88]. Sun and colleagues determined that the E2F/Rb interaction is critical for preventing tumor initiation but that Rb can use context-dependent mechanisms to restrain tumor progression outside of E2F mechanisms as Rb^654^ retains the ability to significantly delay progression to invasive and lethal prostate cancer [88]. This strongly supports our findings that progression to lethal and metastatic prostate cancer occurs independently of E2F-mechanisms [12]. Finally, the tumor suppressor activities of Rb in neoplastic tissues may indeed be attributed primarily to the attenuation of TRIP230 co-activator potential on HIF1-mediated transcriptional responses. Thus, we cannot discount the evidence that supports a role for Rb as a regulator of HIF1-mediated signaling via TRIP230 in prostate cancer progression.

In summary, during the course of activated transcription Rb attenuates the co-activation function of TRIP230 resulting in the appropriate magnitude of mRNA production mediated by the HIF1 complex. Loss of Rb results in the unmasking of the full co-activation potential of TRIP230 leading to exaggerated HIF1 transcriptional output and concomitant up-regulation in target protein expression. The result of this failure is the over-expression of specific hypoxia-regulated transcriptional programs mediating cell transformation and metastasis.

## MATERIALS AND METHODS

### Cell culture

LNCaP (ATCC) and 22Rv1 cells were maintained in RPMI-1640 Media (BioWhittaker, Lonza) with 10% fetal bovine serum (FBS; HyClone, Perbio, Thermo Fisher Scientific Inc.) supplemented with 100 units/mL potassium penicillin-100 μg/mL streptomycin sulphate (BioWhittaker, Lonza), and 4.5 g/L glucose and 4.5 g/L L-glutamine at 37°C, 20% O_2_, and 5% CO_2_. HEK293T cells (ATCC) were maintained in similar conditions as describe above but in Dulbecco's Modified Eagle's Medium (DMEM; BioWhittaker, Lonza) with 10% fetal bovine serum (FBS; HyClone, Perbio, Thermo Fisher Scientific Inc.).

### Quantitative real-time PCR

Real-time PCR (RT-PCR) experiments were performed as described previously. Breifly, LNCaP cells were incubated under hypoxic conditions (1% O_2_) for 24 h in a humidified CO_2_ incubator. The mRNA levels of VEGF, CXCR4, RB1, PLOD2, ENO2, HTR5A, KISS1R, NDRG1 and 36B4 were determined using quantitative real-time PCR. The primer pairs for VEGF, CXCR4, RB1, PLOD2 and 36B4 were described previously [5, 12]. The other primer pairs used were; ENO2: 5′-AG CCATCGACACGGCTGGCTAC-3′ and 5′-TGGACCA GGCAGCCCAATCATC-3′, KISS1R: 5′-CGTTCGGTG CAGTTTCGTTGTGAA-3′ and 5′- CTGGAATGATCCA GAAAGTCCTGTG -3′; NDRG1: 5′- CGCCAGGACA TTGTGAATGAC -3′ and 5′- TTTGAGTTGCACTCCAC CACG -3′; and; HTR5A: 5′- GGCGGACCGTGAACA CCAT-3′ and 5′-ACTCTCCGCTGTCATCTCTCTGG -3′; Total RNA was isolated using TRI reagent (Sigma, Cat. No. T9424–200ML) according to the manufacturer's protocol. Reverse transcription was performed using High Capacity cDNA Reverse Transcription Kit (Applied Biosystems, Part No.4368814) according to the manufacturer's protocol. A total of 2–4 μg of RNA was used in a 20 μL reaction amplified by cycling between 25°C for 5 min, 37°C for 120 min, and 85°C for 5 min (Veriti 96 Well Thermal Cycler, Applied Biosystems). From each experiment, a sample that was both infected with viral Rb-specific shRNA and pre-conditioned with hypoxia was used to generate a relative standard curve in which the sample was diluted 1:10 in five serial dilutions resulting in dilutions of 1:10, 1:100, 1:1,000, 1:10,000, and 1:100,000 whereas the samples were diluted 1:30; the analysis was done using Step One Plus System (Applied Biosystems).

### Immunoblotting

Protein analysis was performed by immunoblotting as described previously [89]. Briefly, LNCaP or 22Rv1 cells were incubated under hypoxic conditions (1% O_2_) for 24 h or up to 7 days as indicated. Cells were harvested and the protein concentration estimated by the Bradford assay. Equal amounts of proteins from the samples were resolved on a SDS-acrylamide gel then transferred to polyvinylidene fluoride (PVDF) membrane. Membranes were probed with primary antibodies and the detection was done using horseradish peroxidase conjugated anti-mouse or anti-rabbit IgG (GE Healthcare,) and ECL Prime detection kit (GE Healthcare).

### Antibodies

Anti-Rb (rabbit polyclonal, Santa Cruz Biotechnology Inc., SC-7905), anti-PLOD2 (mouse polyclonal, Abnova, H00005352-B01P), anti-KISS1R (rabbit polyclonal, Sigma, SAB2700212), anti-HTR5A (rabbit polyclonal, Sigma, SAB2101110), anti-NDRG1 (rabbit polyclonal, Santa Cruz Biotechnology Inc., SC-30040), anti-AR (rabbit polyclonal (PG-21), EMD Millipore, 06–680), anti-RORα (goat polyclonal, Santa Cruz Biotechnology Inc., SC-6062), anti-ENO2 (rabbit polyclonal, Cell Signaling, 9536), anti-α-tubulin (mouse monoclonal, Santa Cruz Biotechnology Inc., SC-8035), goat anti-rabbit IgG-HRP (Santa Cruz Biotechnology Inc., SC-2004), goat anti-mouse IgG-HRP (Santa Cruz Biotechnology Inc., SC-2005), donkey anti-goat IgG-HRP (Santa Cruz Biotechnology Inc., SC-2020).

### Short-hairpin RNA interference in prostate cancer cells

LNCaP or 22Rv1 cells were stably infected with short-hairpin RNAs (shRNA) according to the method described by Wang et al. [90]. The pQCXIPgfp vector was obtained from Dr. Oliver Hankinson (UC, Los Angeles). Oligonucleotides encoding short-hairpin RNAs directed to RB1 were annealed and cloned into the pQCXIPgfp vector 3-prime of the mouse U6 promoter. The RB1 forward and reverse primers were; RB1-CDS/13–14-F–TT TGGGATCTCAGCGATAGAAACTTCAAGAGAGTTT GTATCGCTGTGATCCTTTTT, and; RB1-CDS/13 -14-R -AATTAAAAAGGATCACAGCGATACAAAC TCTCTTGAAGTTTCTATCGCTGAGATCC.

Human embryonic kidney 293T cells were transfected with the shRNA vectors and the pCL10A1 packaging vector using Lipofectamine 2000 (Invitrogen) and maintained in DMEM supplemented with 10% FBS and 1% penicillin/streptomycin. Twenty-four h after transfection, media was replaced and viral supernatants were collected 24 h later. LNCaP and 22RV1cells were seeded into 6-well plates (2 × 10^5^ cells/well) and spin infections were performed using 2 mL of viral supernatant and centrifugation at 2500 rpm for 90 min at 30°C. Twenty-four hours post-infections media was supplemented with 3 μg/mL puromycin. Infection was monitored by immunoflourescence of GFP and knockdown was determined by immunoblotting.

### Matrigel invasion and cell proliferation assays

shRNA LNCaP cells were washed, trypsinized, and re-suspended in culture medium, and subjected to invasion assay using BD BioCoat Matrigel Invasion Chamber (BD Sciences, Cat. No. 354480) according to the manufacturer's protocol. Briefly, the suspended chambers were rehydrated in warm bicarbonate-based medium for 2 h. shRNA LNCaP cells were seeded into invasion chambers in DMEM without FBS at a density of 10, 000 cells/chamber. Chambers were placed in 24-well plates with chemo-attractant (complete medium containing 10% FBS) in the well. The plates were incubated in normoxic (20% O_2_) or hypoxic conditions (1% O_2_) at 37°C for 36 h. Before mounting the invasion membrane to microscope slides, the non-invading cells were removed by cotton swab and invading cells in the membrane were fixed with 100% methanol and stained with 1% toluidine blue. All the cells in the invasion membrane were counted using light microscopy at 10–40 × magnification.

shRNA LNCaP cells either expressing Rb (shSCX) or lacking Rb (shRb) were washed 2 times with PBS, trypsinized and seeded into 6-well plates at 10,000 cells/well. Twenty-four h after plating, cells were either maintained at normal oxygen tensions or treated with 1% O_2_. Cells were counted at; 0 (control), 12, 24, 36, 48 and 72 h following O_2_ treatments. Determinations were performed in triplicate and each sample was counted three times.

### Flow cytometry

Cell cycle status was determined by propidium iodide (PI) staining and flow cytometry. shRNA LNCaP cells were either treated with hypoxia or left at normoxic conditions for 36-hours and then harvested using trypsin. Biological triplicates of 5 × 10^5^ cells were fixed in 70% ethanol on ice for 15 minutes and then cells were centrifuged for 3 minutes at 1500 rpm to remove the ethanol and incubated in 0.5 mL of propidium iodide staining solution (50 μg/mL PI, 0.05% Triton X-100, 0.1 mg/mL RNase A, in PBS) for 40 min at 37°C. Following staining, cells were washed with PBS then run on a BD FACSCanto II flow cytometer (488 nm excitation, 617 emission, 375 volts, PI) where 20,000 events were collected. The percentage of cells in each stage of the cell cycle was determined using FlowJo analysis software based on the PI staining profile of FSC/SSC-gated population.

### Gene expression-array analysis

Gene expression microarray analysis was performed at the Laboratory for Advanced Genome Analysis (Vancouver Prostate Centre, Vancouver, Canada). Messenger RNA from LNCaP cells stably expressing either short-hairpin scrambled RNA (shSCX) or shRb was isolated using TRI reagent (Sigma) according to the manufacturer's protocol. Total RNA was quantified using a NanoDrop ND-1000 UV-VIS spectrophotometer to measure A260/280 and A260/230 ratios. We performed quality control checks of total RNA using an Agilent 2100 Bioanalyzer. One hundred ng of total RNA was converted to cRNA using T7 RNA polymerase in the presence of cyanine 3 (Cy3)-labeled CTP using an Agilent One-Color Microarray-Based Gene Expression Analysis Low Input Quick Amp Labeling v6.0 kit. Experiments were performed in triplicate and cRNAs were hybridized to Agilent GE Human Whole Genome 4 × 44Kv2 microarrays (Design ID 026652).

Arrays were scanned with an Agilent DNA Microarray Scanner at a 3 μm resolution and data was processed using Agilent Feature Extraction 10.10 software. Green processed signal was quantile normalized with Agilent GeneSpring 11.5.1. To find significantly regulated genes, fold changes between the RB1 shRNA and the scrambled shRNA control groups and *p*-values gained from *t*-test between the same groups were calculated with a Benjamini-Hochberg multiple testing correction. The *t*-tests were performed on log transformed normalized data and the variances were not assumed to be equal between sample groups. The data discussed in this publication have been deposited in NCBI's Gene Expression Omnibus [91] and are accessible through GEO Series accession number GSE78245 (http://www.ncbi.nlm.nih.gov/geo/query/acc.cgi?acc%20=%20GSE78245)

Up- and down-regulated genes with *P* values < 0.05 and fold difference ≥ 2.0 compared to the control or to the hypoxia scrambled control group were selected for further analysis. Heat maps were created using the Hierarchical clustering program from the Gene Pattern website (http://genepattern.broadinstitute.org). To map the genes to chromosomal locations, we used the BioMart program located at http://uswest.ensembl.org. The Ensemble Genes 70 and *Homo sapiens* genes (GRCh37.p10) were chosen as databases for analysis. Selected genes from the microarray analysis were mapped on chromosomes by filtering using the Agilent Human Gene Expression 4 × 44 K v2 Microarrays (Design ID 026652) probe's IDs.

### Calcium mobilization assay

shRNA LNCaP and shRNA 22Rv1 cells were either left at normoxia or treated with 1% O_2_ for 96 hours. After hypoxia or normoxia treatment, cells were loaded with 2 μM indo-1AM dye (30 min at 37°C) and then imaged on an inverted fluorescent microscope. Cells were then treated with Kisspeptin-10 peptide and intracellular calcium levels were indexed by ratiometric fluorescence measurements taken at 405 and 485 nm.

### *In silico* data and statistical analysis

DNA sequences for *ATP4A, PLA2G4D, NIM1K, CYP26A1, KISS1R, GPR26, MYBPC2, PCP4L1, AMPD3* and *SCNN1G* were located and defined using the UCSC Genome Browser on Human Dec. 2013 (GRCh38/hg38) Assembly. Annotated DNA sequences as well as 2 Kb upstream of indicated start sites and 2 Kb downstream of indicated stop sites were analyzed for HIF1α:ARNT binding sites using the JASPAR database (http://jaspar.genereg.net) [49, 50]. A 95% relative profile score threshold was used to screen for consensus sequence HIF1 binding sites. If DNA sequences were longer than 20,000 bp then sequential DNA segments were analyzed.

Statistical analyses were performed using GraphPad Prism 4.0. Values for transcriptional responses are presented as means ± standard deviation (S.D.). A *P* value < 0.05 was considered to be significant. Values for growth curves are presented as means ± standard error of the mean (S.E.M.). A *P* value < 0.05 was considered to be significant. For our gene clustering analysis, we used a Wilcoxon rank test in addition to a Student's *t*-test to determine if the distance between target genes was different from the distance between all genes on chromosomes 1 and 21. This was done once for the genes of interest and then for 65 randomly chosen genes from chromosome 1 and 21. For these genes the median distance was calculated. The random choosing was repeated 100 times to generate a distribution of random medians.

## SUPPLEMENTARY MATERIALS TABLE AND FIGURES


