# Supplementary material for: The retinoblastoma protein regulates hypoxia-inducible genetic programs, tumor cell invasiveness and neuroendocrine differentiation in prostate cancer cells
Source: Oncotarget. 2016 Mar 23;7(17):24284–302. doi: 10.18632/oncotarget.8301 (PMC5029701; doi:10.18632/oncotarget.8301)
Supplement: Supplementary file 1 [file oncotarget-07-24284-s001.pdf]

The retinoblastoma protein regulates hypoxia-inducible genetic programs, tumor cell invasiveness and neuroendocrine differentiation in prostate cancer cells

Supplementary Materials

Supplementary Data File 1

Supplementary Table S1: All up-regulated genes that are induced greater than 2-fold by a combination of loss of Rb and hypoxia when compared to all other treatments

		Fold Increase (vs shSCX-N)		
**Gene Name**	**Probe Name**	**shRb-N**	**shSCX-HYP**	**shRb-HYP ± S.D.**
LOC100128264	A_33_P3375279	2.59	61.55	581.19 ± 63.98
HTR5A	A_23_P42565	2.07	27.41	256.40 ± 202.60
PLOD2	A_33_P3318581	2.53	24.26	219.02 ± 25.25
SLC16A3	A_23_P158725	1.73	24.85	200.17 ± 48.02
ATP4A	A_23_P430728	2.46	12.53	157.93 ± 32.25
PLA2G4D	A_33_P3361611	1.15	10.05	97.72 ± 10.46
NIM1	A_23_P254863	1.32	5.73	91.57 ± 80.55
CYP26A1	A_23_P138655	1.15	2.87	68.51 ± 11.92
CXCR4	A_23_P102000	1.30	2.65	62.56 ± 16.56
KISS1R	A_33_P3231357	0.61	8.51	61.94 ± 13.15
ANGPTL4	A_33_P3295358	1.92	3.63	53.17 ± 13.27
GPR26	A_23_P305581	0.98	9.84	53.00 ± 14.08
MYBPC2	A_33_P3257182	1.18	3.74	50.39 ± 4.90
FOS	A_23_P106194	1.47	1.41	49.01 ± 18.72
PPFIA4	A_23_P420692	1.61	8.32	39.76 ± 5.66
CA9	A_23_P157793	1.18	2.09	28.63 ± 6.14
NFATC4	A_33_P3250083	1.52	4.70	21.97 ± 5.61
PFKFB4	A_24_P362904	1.53	2.94	21.82 ± 4.29
PCP4L1	A_32_P214665	1.97	2.51	21.02 ± 0.82
RORA	A_23_P26124	1.40	2.57	20.81 ± 5.43
AMPD3	A_24_P304154	0.91	0.89	19.43 ± 4.15
ALDOC	A_23_P78108	1.46	4.85	19.33 ± 3.21
ENO2	A_24_P236091	1.80	2.61	19.09 ± 2.99
SCNN1G	A_23_P206626	1.10	1.30	19.02 ± 6.66
STC1	A_23_P314755	1.10	4.42	19.00 ± 7.29
NDRG1	A_23_P20494	1.19	6.80	18.6 ± 1.61

TSPEAR	A_23_P394972	5.97	1.71	17.31 ± 7.26
TMEM45A	A_33_P3344831	1.46	2.61	17.07 ± 1.63
MAFF	A_23_P103110	1.23	2.02	16.52 ± 2.85
ATP8B3	A_23_P79108	1.92	2.36	15.52 ± 2.42
GPR146	A_23_P20035	1.35	2.14	15.1 ± 4.01
LOC728276	A_33_P3394362	1.93	1.29	14.94 ± 6.63
BARX1	A_23_P32279	1.50	3.12	14.9 ± 1.80
BEND5	A_23_P904	1.27	2.58	14.25 ± 1.96
NTRK2	A_33_P3322804	1.45	2.36	13.14 ± 3.40
LOC100132354	A_33_P3216282	1.20	1.55	12.94 ± 1.80
PREX1	A_23_P413641	3.01	1.54	12.93 ± 1.44
PAPSS2	A_24_P940166	3.87	2.51	12.81 ± 9.06
C4orf47	A_33_P3247175	1.01	3.09	12.58 ± 3.01
C2orf72	A_33_P3227676	1.18	2.82	12.29 ± 2.69
COL13A1	A_23_P1331	1.37	2.27	12.17 ± 2.00
AFAP1L1	A_32_P167239	1.65	1.58	11.72 ± 1.89
SH3D21	A_23_P307536	0.68	2.72	11.46 ± 1.20
BHLHE40	A_24_P268676	0.94	1.99	11.38 ± 2.29
C20orf46	A_23_P120504	1.66	1.81	11.16 ± 2.12
PLEKHA2	A_33_P3339860	2.02	1.82	10.98 ± 1.40
S1PR4	A_33_P3281273	1.09	4.34	10.62 ± 2.42
FOXD1	A_32_P34920	1.09	2.05	10.44 ± 0.70
LOC442132	A_33_P3406843	0.80	2.50	10.01 ± 5.01
LOC285965	A_33_P3432135	0.83	1.66	10.00 ± 4.16
LOC100291851	A_33_P3328410	1.03	1.80	9.98 ± 1.52
MYOD1	A_33_P3334773	1.03	1.13	9.69 ± 0.80
MORN5	A_24_P401491	3.43	1.81	9.61 ± 6.03
EPB41L4B	A_23_P387656	2.14	0.93	9.59 ± 1.28
PTX3	A_23_P121064	1.34	2.54	9.56 ± 2.18
WDR66	A_24_P179504	1.06	2.09	9.51 ± 2.11
INSIG2	A_33_P3321342	1.22	1.26	9.46 ± 1.53
BIRC3	A_23_P98350	2.90	1.43	9.43 ± 1.47
FLJ44715	A_33_P3671729	1.28	3.57	9.30 ± 0.90
RHOXF1	A_23_P85082	1.69	0.77	8.98 ± 3.28
ANKRD37	A_24_P237586	0.84	2.75	8.80 ± 1.97
BNIP3	A_33_P3419785	1.11	3.20	8.50 ± 1.48
LDLRAD1	A_24_P491397	2.62	1.01	8.45 ± 2.91
STON1	A_23_P302005	2.13	1.09	8.26 ± 1.07
CYB5R2	A_23_P2181	1.13	1.72	8.14 ± 0.89

C8orf22	A_32_P80741	0.71	0.84	8.02 ± 11.98
NEDD9	A_23_P344555	2.04	1.85	7.84 ± 3.40
P4HA1	A_33_P3214481	1.10	3.35	7.81 ± 0.87
PHGR1	A_33_P3323501	3.23	1.84	7.78 ± 0.66
LGALS8	A_33_P3349883	3.61	1.80	7.70 ± 1.76
KRTAP10–12	A_33_P3357651	1.53	1.21	7.60 ± 2.00
EGFR	A_23_P215790	0.80	1.96	7.57 ± 0.53
ITPKA	A_23_P65918	2.21	1.28	7.53 ± 0.30
CYS1	A_33_P3398156	1.01	1.11	7.44 ± 1.63
PFKP	A_33_P3413962	0.88	2.57	7.41 ± 0.83
PFKFB3	A_24_P261259	0.89	1.80	7.28 ± 0.82
HES7	A_33_P3368695	1.47	1.43	7.27 ± 1.28
LOC644192	A_24_P795371	0.96	1.43	7.20 ± 1.14
LOC154761	A_24_P918907	1.06	2.39	7.18 ± 2.09
C10orf11	A_23_P113034	1.66	1.37	7.09 ± 1.25
SYT17	A_23_P163697	1.34	2.14	7.06 ± 0.55
NPR3	A_23_P58676	0.99	2.18	6.99 ± 2.53
CXCL12	A_24_P412156	3.22	2.68	6.92 ± 3.48
TUBB2B	A_24_P314477	2.04	2.05	6.88 ± 1.19
GREM2	A_24_P40626	0.74	0.92	6.86 ± 1.47
BNIP3L	A_23_P134925	1.00	2.42	6.82 ± 0.82
LOX	A_23_P122216	1.11	1.92	6.79 ± 2.27
FLJ26484	A_33_P3741022	0.92	1.32	6.71 ± 5.13
NRXN2	A_24_P261470	1.87	1.65	6.65 ± 1.16
DLEC1	A_23_P18282	1.34	1.15	6.61 ± 1.76
VEGFB	A_23_P1594	1.59	2.33	6.54 ± 0.37
LOC100128988	A_32_P202125	1.23	1.06	6.53 ± 1.75
DHRS3	A_23_P33759	1.75	1.66	6.47 ± 0.57
TMEM45B	A_23_P1682	1.67	1.61	6.35 ± 0.66
TCP11L2	A_23_P419107	1.01	1.42	6.34 ± 1.20
CYBRD1	A_24_P345451	2.41	1.74	6.33 ± 0.44
LOC388242	A_32_P135336	1.67	1.29	6.33 ± 0.92
FLJ37644	A_24_P497464	1.83	1.18	6.29 ± 2.58
PTH1R	A_23_P167030	2.26	2.53	6.23 ± 2.31
FAM110C	A_32_P47643	1.92	1.39	6.20 ± 1.06
KDM3A	A_23_P395075	1.17	2.41	6.18 ± 1.78
PAQR5	A_33_P3368750	1.31	1.60	6.15 ± 1.19
SGCE	A_33_P3229617	2.95	1.78	6.05 ± 1.95
RASGEF1A	A_33_P3287760	1.87	1.58	6.03 ± 3.84

ARAP3	A_23_P167389	2.11	2.14	5.89 ± 0.85
CYP1B1	A_23_P209625	1.02	2.45	5.87 ± 1.54
LOC399715	A_33_P3291619	0.55	2.14	5.86 ± 1.33
GAD1	A_23_P374689	0.56	1.73	5.84 ± 3.48
ASGR1	A_23_P118722	1.92	1.75	5.83 ± 0.76
VNN2	A_23_P122724	1.55	1.41	5.77 ± 0.73
C15orf62	A_32_P183970	1.67	1.30	5.69 ± 1.64
PPP1R3B	A_24_P201064	1.04	1.53	5.65 ± 0.69
ODAM	A_23_P58228	1.59	2.16	5.62 ± 0.64
WISP2	A_23_P102611	1.23	1.89	5.60 ± 2.38
EPO	A_23_P145669	0.95	2.16	5.57 ± 2.53
SEC14L5	A_24_P254850	1.00	1.03	5.57 ± 1.48
DUSP5P	A_24_P367602	1.09	2.23	5.56 ± 0.84
STAB1	A_23_P32500	1.36	2.65	5.56 ± 1.37
ASCL2	A_32_P171061	1.14	2.55	5.55 ± 0.69
SH2D3C	A_33_P3293918	2.61	2.02	5.36 ± 2.26
PADI2	A_24_P187970	1.99	1.42	5.35 ± 0.44
CCDC147	A_33_P3257297	1.59	1.37	5.35 ± 1.40
ATP8A2	A_23_P258612	1.35	1.52	5.34 ± 2.58
GATA5	A_23_P371835	1.16	1.86	5.31 ± 1.58
ANKZF1	A_23_P119907	1.16	2.03	5.23 ± 0.93
SRRM3	A_23_P331700	2.12	1.73	5.22 ± 1.37
ZNF395	A_23_P146077	1.27	1.64	5.20 ± 0.85
P4HA2	A_23_P30363	1.01	1.77	5.08 ± 0.35
VIM	A_23_P161190	0.59	2.22	5.01 ± 0.30
MAP3K15	A_23_P308483	1.06	1.58	5.01 ± 0.84
TCAM1P	A_24_P68088	1.46	0.79	5.00 ± 3.75
C20orf195	A_33_P3405459	1.50	1.49	4.97 ± 0.42
KIAA2022	A_32_P169505	0.80	1.55	4.96 ± 1.17
STON1-GTF2A1L	A_23_P79488	0.71	0.93	4.91 ± 3.19
SH3PXD2A	A_23_P35456	1.59	1.41	4.91 ± 0.23
ADAM12	A_33_P3310929	1.00	1.32	4.87 ± 0.75
DNAI2	A_33_P3419239	1.16	1.00	4.87 ± 2.20
PGK1	A_23_P125829	0.98	2.00	4.85 ± 0.30
AKAP12	A_23_P111311	0.62	1.72	4.82 ± 1.44
FAM115C	A_24_P237912	0.98	1.73	4.81 ± 1.03
SH3GL3	A_23_P48988	1.63	1.29	4.80 ± 0.46
TG	A_23_P32454	0.95	1.61	4.79 ± 0.67
ARC	A_23_P365738	0.80	1.98	4.77 ± 1.56

SCN4B	A_23_P303833	0.89	1.65	4.75 ± 0.68
DPYSL4	A_23_P331049	0.86	1.72	4.74 ± 0.82
GPR155	A_32_P132317	1.73	1.30	4.70 ± 0.40
FLJ31356	A_33_P3364062	1.02	1.26	4.68 ± 1.47
ZNF292	A_32_P113584	0.94	1.54	4.67 ± 0.50
EPAS1	A_23_P210210	1.49	1.06	4.65 ± 0.69
RAB33A	A_23_P147025	1.05	1.61	4.64 ± 0.77
PHF21B	A_24_P113572	1.52	1.40	4.62 ± 2.65
CDKN1C	A_23_P428129	1.32	1.65	4.61 ± 0.47
C11orf96	A_32_P74409	1.08	1.32	4.60 ± 0.77
OSBPL7	A_33_P3358740	0.82	2.02	4.60 ± 1.43
MMP24	A_33_P3398331	0.91	1.74	4.56 ± 0.59
CACNA1H	A_33_P3218960	0.72	1.20	4.55 ± 0.87
PDK1	A_24_P37441	0.98	1.90	4.52 ± 0.19
IGFBP6	A_23_P139912	1.43	1.07	4.52 ± 0.92
CCDC110	A_23_P383325	1.54	1.68	4.51 ± 0.56
LCN15	A_33_P3263938	1.36	1.26	4.50 ± 0.50
MAP7D2	A_24_P367645	0.64	1.86	4.44 ± 4.22
TFF3	A_23_P393099	2.17	1.42	4.43 ± 0.33
FUT11	A_23_P413788	0.92	2.10	4.43 ± 0.49
TCF7L1	A_23_P142872	1.50	0.76	4.38 ± 3.43
MUC6	A_33_P3314500	1.36	1.10	4.38 ± 1.10
PLK5	A_32_P50223	1.20	0.96	4.32 ± 0.70
GADD45B	A_24_P239606	1.12	1.52	4.30 ± 0.23
SPRY1	A_23_P144476	0.83	1.47	4.28 ± 0.45
EFNA3	A_24_P114032	1.81	1.67	4.27 ± 1.51
DSP	A_33_P3402565	1.21	1.53	4.23 ± 0.62
CIB2	A_33_P3308914	1.76	1.26	4.22 ± 0.07
SEMA4G	A_23_P127068	1.64	1.35	4.19 ± 0.51
LOC727869	A_32_P110016	1.09	1.57	4.17 ± 0.46
FAM167A	A_23_P334955	1.79	1.18	4.15 ± 1.31
RIMKLA	A_23_P349406	0.84	1.52	4.15 ± 0.41
CA8	A_23_P83838	2.03	1.01	4.14 ± 0.81
FAM13A	A_33_P3691916	1.33	1.34	4.13 ± 1.11
SLC1A1	A_23_P216468	1.10	1.75	4.13 ± 0.78
ZC3H6	A_24_P826348	1.21	1.17	4.10 ± 0.49
YPEL2	A_24_P787947	1.39	1.04	4.06 ± 0.55
RNF182	A_23_P399255	1.75	1.32	4.05 ± 0.46
UNC5CL	A_23_P428298	1.28	1.36	4.05 ± 1.03

LRAT	A_32_P113066	1.00	0.99	4.04 ± 0.56
ST3GAL6	A_23_P250800	1.05	0.74	4.01 ± 0.71
NCRNA00324	A_23_P362191	1.25	1.57	4.01 ± 1.09
HES1	A_23_P6596	1.73	1.14	4.01 ± 0.68
TGFB1	A_24_P79054	0.94	0.94	3.98 ± 1.41
S100A10	A_23_P137984	0.75	1.76	3.97 ± 0.43
HPCA	A_33_P3246133	1.53	0.89	3.97 ± 0.77
TMPRSS2	A_23_P29067	1.64	1.21	3.94 ± 0.31
RAB6B	A_24_P475349	1.58	1.32	3.90 ± 0.18
CLIP3	A_23_P50786	1.18	1.42	3.89 ± 1.03
GPA33	A_24_P319374	1.88	1.51	3.88 ± 1.01
SLC2A1	A_23_P571	0.85	1.81	3.88 ± 0.39
DUSP1	A_23_P110712	1.01	0.88	3.85 ± 1.10
ARMCX4	A_33_P3211864	1.21	1.27	3.85 ± 0.79
EFEMP2	A_33_P3367830	1.05	1.39	3.84 ± 0.62
GBE1	A_23_P121082	0.84	1.47	3.83 ± 0.41
ERO1L	A_23_P106145	0.97	1.30	3.83 ± 0.51
LDHC	A_23_P53039	0.95	1.57	3.82 ± 0.78
NCAM1	A_33_P3363799	1.41	1.16	3.81 ± 1.27
ITGB2	A_23_P329573	1.00	1.80	3.80 ± 0.61
C4orf3	A_23_P388433	0.92	1.67	3.79 ± 0.12
NHEDC1	A_23_P415611	1.37	0.84	3.78 ± 0.97
STBD1	A_23_P254079	1.20	1.30	3.77 ± 1.55
MVP	A_33_P3294608	1.29	0.89	3.77 ± 0.52
ZNF404	A_23_P90333	1.34	1.33	3.76 ± 0.35
PRPH	A_23_P13713	0.90	1.65	3.74 ± 0.23
CCND2	A_24_P278747	0.98	1.57	3.72 ± 1.32
VEGFA	A_24_P12401	0.94	1.86	3.72 ± 0.69
C1orf51	A_23_P410717	1.13	1.61	3.70 ± 0.98
PCP2	A_24_P399500	1.14	1.21	3.67 ± 0.18
B3GNT4	A_23_P76071	1.36	1.50	3.65 ± 0.38
ASB2	A_23_P205370	0.92	1.63	3.64 ± 0.10
GLDN	A_24_P49199	1.76	1.54	3.64 ± 1.15
NOTCH3	A_33_P3313055	1.66	1.37	3.64 ± 0.61
LOC100130691	A_24_P400172	1.46	1.01	3.63 ± 2.75
SPTB	A_32_P134968	1.41	1.19	3.61 ± 0.24
FZD7	A_23_P209449	1.47	1.18	3.60 ± 0.59
MXI1	A_33_P3383029	0.90	1.43	3.57 ± 0.67
LOC100240735	A_32_P170481	1.58	1.58	3.55 ± 1.67

RNF165	A_33_P3319680	0.71	1.33	3.55 ± 0.87
ARHGEF37	A_32_P222695	1.19	1.71	3.53 ± 0.23
BEST4	A_33_P3244808	1.28	1.19	3.51 ± 0.55
LRP1	A_23_P124837	1.24	1.32	3.51 ± 0.71
MLLT3	A_23_P216693	1.14	0.91	3.51 ± 0.35
CREG2	A_23_P394986	0.92	1.18	3.49 ± 0.59
RNASE4	A_23_P205531	1.44	1.70	3.49 ± 0.65
C5orf41	A_23_P404606	0.93	1.24	3.49 ± 0.58
PNMA2	A_24_P389415	1.59	1.32	3.48 ± 0.41
ZNF511	A_33_P3384885	1.08	1.38	3.47 ± 0.36
KIAA1984	A_33_P3293511	1.07	1.49	3.45 ± 1.18
ARID3A	A_33_P3272921	0.90	1.35	3.44 ± 0.50
KLHL3	A_23_P133543	1.49	1.70	3.44 ± 0.58
TTC6	A_33_P3226575	1.26	1.23	3.44 ± 0.29
FOSL2	A_23_P348121	1.09	1.10	3.44 ± 0.47
LOC284998	A_24_P498854	0.62	1.58	3.43 ± 0.53
VIPR2	A_32_P153071	1.50	1.64	3.42 ± 0.44
RLF	A_23_P126037	0.98	1.55	3.40 ± 0.29
NLGN1	A_33_P3214690	1.66	0.87	3.40 ± 1.02
VPS37D	A_33_P3293573	1.12	1.30	3.38 ± 0.24
SCNN1B	A_32_P83098	0.98	0.87	3.33 ± 0.66
C4orf6	A_23_P7048	0.67	1.18	3.32 ± 1.34
NOTUM	A_33_P3257125	1.12	1.13	3.31 ± 0.67
VSTM2A	A_33_P3347193	1.03	1.42	3.31 ± 0.38
LAMB2P1	A_33_P3413815	1.29	0.77	3.31 ± 0.38
RNF112	A_23_P107116	0.65	1.44	3.26 ± 0.31
GRID1	A_23_P32805	1.31	1.37	3.25 ± 0.76
LOC100129617	A_33_P3343516	1.15	1.35	3.25 ± 1.01
PNRC1	A_23_P145074	1.21	1.25	3.25 ± 0.17
GLYAT	A_23_P75749	1.06	1.06	3.24 ± 0.69
SLC35F3	A_23_P422212	0.98	1.27	3.24 ± 0.51
GLIS2	A_33_P3333224	1.35	1.01	3.23 ± 0.31
CCNG2	A_24_P12065	1.37	1.53	3.23 ± 0.86
ABTB1	A_33_P3297444	1.28	1.28	3.22 ± 0.16
IL10RA	A_23_P203173	1.06	1.55	3.22 ± 0.71
JAK3	A_24_P59667	1.25	1.16	3.21 ± 0.46
TBC1D8B	A_23_P325887	1.19	1.22	3.19 ± 0.42
TLE6	A_33_P3400147	1.24	1.23	3.18 ± 0.37
RASD1	A_23_P118392	1.03	1.19	3.18 ± 0.98

TNNT1	A_33_P3397865	1.18	1.42	3.17 ± 0.36
PPP2R5B	A_23_P35796	1.26	1.19	3.17 ± 0.41
BCL3	A_23_P4662	0.97	1.02	3.16 ± 0.40
CLK3	A_32_P20691	0.94	1.18	3.15 ± 0.21
ZNF713	A_33_P3409710	1.39	0.86	3.14 ± 1.55
SEC31B	A_23_P35564	1.34	1.35	3.14 ± 0.70
BRSK2	A_33_P3273480	1.26	1.24	3.13 ± 0.30
PRKAA2	A_32_P94160	1.27	1.25	3.12 ± 0.21
GATA2	A_33_P3550894	1.12	1.11	3.12 ± 0.51
MYO15A	A_23_P27229	0.91	1.32	3.11 ± 1.43
DUSP5	A_23_P150018	0.90	1.13	3.10 ± 1.13
CD300A	A_24_P159434	0.89	0.85	3.10 ± 0.77
CLRN3	A_23_P127107	1.09	0.92	3.09 ± 0.58
JUND	A_33_P3324909	1.36	1.03	3.08 ± 1.00
IGFL3	A_33_P3243454	1.09	1.47	3.07 ± 1.84
C7orf68	A_23_P20022	0.77	1.13	3.06 ± 0.11
HMOX1	A_23_P120883	0.82	0.99	3.04 ± 0.19
GUCY1A2	A_23_P350001	0.89	1.37	3.04 ± 1.39
ALOX5	A_23_P104464	1.42	1.26	3.03 ± 0.32
TGFBI	A_23_P156327	1.51	1.27	3.01 ± 0.55
CLEC3B	A_23_P69497	0.99	1.11	2.99 ± 0.14
TSC22D3	A_33_P3237634	0.91	1.28	2.98 ± 0.34
ZBTB20	A_23_P40866	1.02	0.87	2.97 ± 0.42
BLNK	A_33_P3363637	1.41	1.29	2.97 ± 0.19
FRY	A_33_P3223495	0.97	1.46	2.95 ± 0.43
PLTP	A_23_P5983	1.26	1.34	2.95 ± 0.23
COX8C	A_33_P3372069	0.87	0.96	2.93 ± 0.63
YPEL1	A_32_P88120	1.07	1.25	2.92 ± 0.39
CRYM	A_23_P77731	1.37	1.31	2.91 ± 0.26
SMCR6	A_33_P3877728	1.27	1.34	2.91 ± 0.51
RNF122	A_23_P134744	0.86	1.37	2.90 ± 0.24
HERC3	A_24_P51201	0.51	0.86	2.87 ± 0.68
CAMK1D	A_23_P124252	0.88	1.32	2.87 ± 0.46
VASN	A_23_P129695	1.14	1.35	2.84 ± 0.27
YEATS2	A_23_P69437	0.86	1.20	2.84 ± 0.08
MTL5	A_23_P161507	1.14	1.30	2.83 ± 0.43
ZSWIM5	A_23_P383118	0.99	0.93	2.82 ± 0.54
GAL3ST1	A_23_P120863	0.61	0.92	2.81 ± 0.98
CARHSP1	A_33_P3260307	1.30	1.19	2.81 ± 0.15

LOC284930	A_33_P3436935	0.93	1.21	2.79 ± 0.54
MST1	A_24_P148796	1.14	1.26	2.79 ± 0.29
KIAA1467	A_23_P394567	1.13	1.22	2.79 ± 0.42
C17orf39	A_33_P3381771	1.25	1.29	2.79 ± 0.29
LOC283070	A_33_P3464555	0.87	1.32	2.78 ± 0.44
IFITM10	A_33_P3358601	1.08	1.22	2.76 ± 0.74
KLHL24	A_24_P521994	0.82	1.26	2.76 ± 0.37
DDRGK1	A_33_P3299781	0.89	1.15	2.75 ± 1.00
BHLHA15	A_33_P3312730	1.10	1.22	2.73 ± 0.33
PNCK	A_23_P62377	0.82	1.16	2.71 ± 1.50
MICALL2	A_24_P303524	1.25	1.26	2.70 ± 0.10
HIVEP2	A_23_P214766	1.19	0.98	2.70 ± 0.36
LPIN3	A_33_P3393341	1.07	1.30	2.70 ± 0.24
RSBN1	A_33_P3888365	0.97	1.12	2.70 ± 0.34
C7orf63	A_33_P3615922	0.93	0.86	2.68 ± 0.32
WSB1	A_23_P4353	1.03	1.19	2.67 ± 0.48
PYGL	A_23_P48676	0.98	1.17	2.67 ± 0.35
ACLY	A_33_P3415042	1.19	1.21	2.66 ± 0.44
MICAL3	A_24_P366082	1.20	1.15	2.66 ± 0.54
CCDC114	A_33_P3261054	1.22	1.32	2.65 ± 0.42
DCDC2	A_33_P3212109	1.06	0.99	2.65 ± 0.87
EIF2C4	A_23_P23171	1.00	1.25	2.65 ± 0.39
SYNPO	A_33_P3397658	1.14	1.06	2.63 ± 0.64
HLA-A	A_23_P408353	1.23	1.09	2.62 ± 0.18
IRS1	A_24_P802145	1.28	0.99	2.61 ± 0.44
TNIP1	A_23_P30435	1.28	1.16	2.60 ± 0.35
CITED2	A_23_P214969	1.02	1.13	2.59 ± 0.24
IGSF21	A_32_P78101	0.72	1.28	2.58 ± 0.15
PLXND1	A_24_P376391	1.24	1.18	2.57 ± 0.17
LUC7L3	A_33_P3246997	1.16	1.13	2.55 ± 0.51
RIPK4	A_24_P125871	1.16	1.19	2.55 ± 0.23
ANKRD12	A_33_P3294524	1.00	1.18	2.54 ± 0.60
JUN	A_33_P3323298	0.95	1.05	2.54 ± 0.73
PTGER4	A_23_P148047	1.13	1.01	2.54 ± 0.20
DENND3	A_33_P3321130	0.99	1.06	2.52 ± 0.84
CDKN1B	A_24_P81841	1.20	1.06	2.52 ± 0.31
C8orf58	A_23_P310483	1.12	1.06	2.52 ± 0.31
CHST6	A_23_P106922	1.21	0.94	2.52 ± 0.37
NRG3	A_33_P3229417	1.12	0.62	2.51 ± 0.51

SAV1	A_24_P287473	1.05	1.16	2.50 ± 0.22
SNTB1	A_23_P95029	0.92	0.93	2.49 ± 0.57
MRC2	A_33_P3364741	0.97	1.23	2.49 ± 0.57
PPP1R13L	A_23_P119095	1.10	1.20	2.49 ± 0.30
GSN	A_33_P3423365	1.24	1.12	2.48 ± 0.37
YPEL5	A_33_P3281408	1.00	1.16	2.48 ± 0.37
PRTN3	A_23_P142345	1.04	0.87	2.48 ± 0.51
LOC100131346	A_33_P3289338	1.15	1.05	2.48 ± 0.20
RIT1	A_33_P3394075	0.68	0.90	2.46 ± 0.47
ARL4C	A_33_P3323722	0.90	1.06	2.45 ± 0.34
C16orf89	A_33_P3296862	0.96	1.20	2.45 ± 0.42
MKNK2	A_23_P142310	1.16	1.11	2.45 ± 0.22
PIAS2	A_33_P3349269	0.93	1.11	2.44 ± 0.23
STC2	A_23_P416395	0.90	1.19	2.44 ± 0.28
MYO1E	A_33_P3297978	1.09	1.07	2.44 ± 0.35
LOC100134285	A_33_P3240972	0.82	0.92	2.43 ± 0.96
EFCAB3	A_33_P3264042	0.86	1.15	2.43 ± 0.29
LOC283050	A_33_P3394140	0.71	1.07	2.40 ± 0.35
FLJ41455	A_33_P3520835	0.95	1.15	2.40 ± 0.41
CECR5-AS1	A_32_P131143	0.96	1.11	2.39 ± 0.36
FLJ32224	A_33_P3694746	1.04	0.93	2.38 ± 0.20
C17orf76	A_23_P368484	1.03	1.04	2.37 ± 0.34
MAPT	A_24_P224488	0.66	1.10	2.37 ± 0.53
KIAA1958	A_33_P3404744	1.04	0.92	2.35 ± 0.24
EVC2	A_33_P3319880	0.90	0.83	2.32 ± 1.43
RNF24	A_24_P333019	1.11	1.09	2.32 ± 0.58
KIAA1715	A_32_P31771	1.03	1.16	2.32 ± 0.16
BRWD3	A_32_P489130	1.06	1.11	2.32 ± 0.52
DMGDH	A_33_P3387716	0.95	0.81	2.31 ± 0.52
EPB41L4A	A_24_P257579	0.97	0.85	2.27 ± 0.43
WTIP	A_33_P3381948	0.99	1.04	2.26 ± 0.47
TNFRSF10D	A_33_P3326588	1.11	1.04	2.25 ± 0.21
GRIN3B	A_33_P3394213	0.98	0.91	2.24 ± 0.34
TNFRSF25	A_33_P3234530	0.85	0.76	2.22 ± 1.20
CASC2	A_33_P3213362	1.05	0.83	2.20 ± 0.32
GSDMB	A_33_P3221999	1.10	1.07	2.19 ± 0.17
BBC3	A_23_P382775	0.93	1.00	2.14 ± 0.14
AQPEP	A_33_P3251522	0.88	0.83	2.13 ± 1.22
CRHR1	A_33_P3320443	0.94	1.02	2.12 ± 0.18

FAM117B	A_32_P195401	0.92	0.99	2.11 ± 0.25
PAGE2B	A_33_P3420862	0.91	1.02	2.11 ± 0.26
PARD3	A_24_P35478	0.89	0.85	2.09 ± 0.62
CDKL3	A_23_P110643	0.94	0.99	2.09 ± 0.20
APOE	A_33_P3223592	0.98	0.84	2.05 ± 0.23
LOC100127885	A_33_P3278813	0.99	0.73	2.05 ± 1.12

Supplementary Table S2: All down-regulated genes that are attenuated greater than 2-fold by a combination of loss of Rb and hypoxia when compared to all other treatments

		Fold Decrease (vs shSCX-N)		
**Gene Name**	**Probe Name**	**shRb-N**	**shSCX-HYP**	**shRb-HYP ± S.D.**
SPIC	A_32_P148275	2.00	0.95	8.55 ± 2.91
DSC1	A_23_P38696	2.24	0.89	7.92 ± 1.67
KBTBD8	A_23_P431252	1.75	1.21	7.83 ± 2.00
UGT2B4	A_23_P386912	1.65	1.09	7.66 ± 3.60
PTPRQ	A_33_P3309471	2.39	1.00	7.21 ± 1.32
EOMES	A_24_P97374	1.50	1.03	6.84 ± 1.43
FLJ45684	A_33_P3288219	1.60	1.27	6.48 ± 1.82
CLEC2A	A_33_P3295348	1.05	0.94	6.11 ± 2.65
TARP	A_33_P3225625	2.68	0.87	6.11 ± 2.63
KLHL1	A_23_P2825	1.63	1.38	6.08 ± 0.96
TAF9B	A_24_P391431	1.31	1.27	5.92 ± 0.89
INSL5	A_23_P51479	1.11	1.15	5.77 ± 2.23
LRRTM4	A_24_P174294	2.61	0.99	5.63 ± 0.77
TBX15	A_24_P128442	2.55	1.42	5.46 ± 0.94
NAP1L3	A_23_P125717	1.17	1.26	5.37 ± 1.07
CNGB3	A_23_P216376	1.57	1.68	4.89 ± 1.81
POLR3G	A_33_P3396527	2.30	1.22	4.82 ± 0.46
KLB	A_23_P350617	1.71	0.86	4.65 ± 0.82
MARS2	A_23_P108492	1.04	1.27	4.32 ± 1.98
ASZ1	A_33_P3217258	1.72	1.14	4.19 ± 0.56
ZNF365	A_24_P226970	1.13	1.20	4.17 ± 0.78
GBX2	A_33_P3423969	1.15	1.31	4.16 ± 0.44
ARHGAP9	A_23_P64661	1.64	1.09	4.04 ± 0.67
DPF3	A_33_P3240946	1.59	1.46	4.02 ± 1.81
PKHD1L1	A_33_P3254136	0.78	0.85	3.89 ± 1.47
MPV17L2	A_23_P165130	1.19	1.31	3.87 ± 0.40

NCRNA00167	A_24_P359030	0.83	1.04	3.84 ± 1.67
FLJ40606	A_33_P3388636	1.27	1.14	3.82 ± 0.68
PSPC1	A_33_P3232173	1.65	1.05	3.73 ± 0.96
POP1	A_23_P341275	1.44	1.47	3.69 ± 0.37
CLEC7A	A_24_P235988	1.66	0.92	3.60 ± 0.85
HSPH1	A_33_P3348752	1.21	0.98	3.53 ± 0.35
CNDP1	A_23_P9869	0.75	0.50	3.51 ± 0.88
SCAF11	A_33_P3283196	1.29	1.07	3.49 ± 0.52
CSMD1	A_33_P3230249	1.45	1.50	3.47 ± 0.98
FGFBP3	A_24_P201381	1.59	1.05	3.47 ± 0.79
AREG	A_33_P3419190	1.00	1.09	3.43 ± 1.04
MSMB	A_24_P146683	1.09	1.18	3.39 ± 0.64
UFSP1	A_33_P3327500	1.31	1.09	3.38 ± 0.43
LRRN4CL	A_33_P3221129	1.00	1.23	3.35 ± 0.78
CHAC2	A_32_P194264	1.00	1.09	3.34 ± 0.67
AGPAT9	A_23_P69810	1.36	1.21	3.33 ± 1.00
SLC2A13	A_33_P3354514	1.52	0.93	3.32 ± 0.96
POLR1B	A_24_P942112	1.39	1.27	3.27 ± 0.39
PIGW	A_23_P379794	1.14	1.06	3.24 ± 0.57
SNAPC5	A_33_P3335746	0.95	1.12	3.19 ± 0.45
SFPQ	A_33_P3318292	1.00	1.08	3.17 ± 0.19
FZD8	A_23_P396858	1.40	1.03	3.16 ± 0.61
NUP98	A_23_P308032	1.18	1.10	3.15 ± 0.20
MAPK10	A_23_P45025	1.05	1.24	3.14 ± 1.08
YIF1B	A_23_P142239	0.93	1.41	3.13 ± 0.67
ZNF597	A_24_P378402	1.07	1.46	3.12 ± 0.97
PNLIPRP3	A_33_P3368193	0.98	0.87	3.04 ± 1.14
SLC30A1	A_23_P23815	1.29	1.43	3.03 ± 0.36
AP1S3	A_33_P3288995	0.98	1.28	3.03 ± 0.41
GPATCH4	A_33_P3211263	1.08	1.11	3.00 ± 0.19
SGOL1	A_23_P29723	0.98	1.03	2.94 ± 0.59
ING3	A_33_P3356711	1.20	1.45	2.94 ± 0.32
SLC43A2	A_24_P296508	1.41	1.15	2.93 ± 0.26
RRS1	A_23_P146187	1.24	1.31	2.88 ± 0.14
WT1	A_23_P116280	0.98	1.27	2.87 ± 0.48
FITM2	A_24_P203407	1.19	1.10	2.87 ± 0.13
OXNAD1	A_24_P927189	1.38	1.27	2.86 ± 0.57
CROT	A_33_P3355208	1.32	1.26	2.82 ± 0.30
TMEM177	A_23_P5339	0.89	1.12	2.80 ± 0.53
ELAC1	A_23_P15944	1.15	1.04	2.79 ± 0.30

HGD	A_23_P250164	1.36	0.91	2.79 ± 0.29
CD244	A_33_P3234292	1.05	1.14	2.78 ± 0.43
MSGN1	A_33_P3291379	1.07	0.86	2.78 ± 1.58
GLRX2	A_23_P160503	1.37	1.05	2.78 ± 0.29
CYCSP52	A_33_P3261132	1.23	1.27	2.76 ± 0.74
CHORDC1	A_33_P3502037	1.18	0.99	2.75 ± 0.35
STIM2	A_24_P180242	1.13	1.10	2.74 ± 0.95
GNRH2	A_24_P323072	1.09	1.08	2.74 ± 0.30
SLC5A6	A_24_P247732	1.13	1.24	2.74 ± 0.53
TM4SF18	A_24_P120251	1.30	1.06	2.72 ± 0.89
MAGOHB	A_24_P330112	0.92	1.12	2.71 ± 0.30
PGAM5	A_23_P319719	1.25	1.13	2.70 ± 0.30
KTI12	A_23_P103276	1.25	1.23	2.69 ± 0.15
FLJ13744	A_33_P3806965	1.09	1.08	2.68 ± 0.33
NOP2	A_23_P204364	1.04	1.19	2.68 ± 0.55
SURF6	A_24_P21447	1.19	1.25	2.66 ± 0.14
PNN	A_33_P3218694	1.29	1.00	2.64 ± 0.25
PYCRL	A_23_P383278	1.08	1.05	2.63 ± 0.36
PCYT2	A_33_P3394809	1.11	1.13	2.63 ± 0.30
ADAT1	A_23_P141100	0.91	1.03	2.62 ± 0.40
PNO1	A_24_P336853	1.12	1.10	2.62 ± 0.44
TRPM2	A_24_P27977	1.02	1.05	2.61 ± 0.26
SRSF7	A_24_P222911	1.20	0.91	2.61 ± 0.27
ARL4A	A_32_P806841	1.09	1.27	2.59 ± 0.17
RPS6KL1	A_33_P3334419	1.13	1.07	2.58 ± 0.27
NCDN	A_23_P97736	0.97	1.11	2.57 ± 0.32
UQCR10	A_24_P66001	1.25	1.15	2.57 ± 0.09
TIMM13	A_33_P3413845	1.24	1.18	2.56 ± 0.43
NDUFAF4	A_24_P171983	1.10	1.17	2.56 ± 0.21
SRPRB	A_23_P80773	1.15	1.10	2.56 ± 0.32
L1CAM	A_33_P3374443	1.15	0.82	2.56 ± 0.76
ADRB1	A_33_P3310189	1.15	1.21	2.54 ± 0.32
PDSS1	A_23_P161152	1.22	1.12	2.53 ± 0.34
AP4B1	A_23_P160729	1.03	1.07	2.52 ± 0.22
FUS	A_23_P106887	0.78	0.95	2.51 ± 0.43
FAM203A	A_23_P61268	1.06	1.16	2.51 ± 0.18
CLDN8	A_23_P427014	0.73	1.11	2.49 ± 0.67
JMJD4	A_33_P3306113	1.24	1.15	2.49 ± 0.28
NDUFC2	A_24_P364236	1.11	0.99	2.49 ± 0.11
HAS3	A_23_P393034	0.91	1.21	2.47 ± 0.25

MSTO1	A_23_P630	1.23	1.13	2.47 ± 0.35
TOE1	A_24_P19828	0.99	1.10	2.46 ± 0.26
TOR3A	A_24_P130962	1.01	1.02	2.45 ± 0.30
NOP56	A_23_P79927	0.96	1.01	2.44 ± 0.51
RRP9	A_23_P6802	1.13	1.18	2.43 ± 0.15
CDC25A	A_24_P397107	1.00	1.08	2.43 ± 0.44
TRAM1L1	A_23_P18518	1.02	1.06	2.42 ± 0.17
CLN6	A_23_P117797	1.01	1.05	2.41 ± 0.25
PLG	A_23_P30693	0.73	0.80	2.40 ± 0.43
MKI67IP	A_23_P50897	1.19	1.05	2.38 ± 0.25
ZBTB9	A_23_P8119	1.10	1.14	2.37 ± 0.45
SLC27A4	A_24_P257971	0.91	0.99	2.37 ± 0.67
MUM1	A_23_P208961	0.92	1.04	2.37 ± 0.23
NOL6	A_24_P21410	0.99	1.15	2.37 ± 0.37
NRADDP	A_33_P3386150	0.90	1.08	2.36 ± 0.46
SRSF3	A_33_P3232828	1.04	1.08	2.35 ± 0.07
RPP30	A_33_P3233666	1.12	1.01	2.35 ± 0.36
ABCA1	A_24_P235429	0.68	1.14	2.34 ± 0.25
SLC25A20	A_23_P72025	0.98	1.11	2.34 ± 0.23
CCNE2	A_33_P3217819	0.95	1.06	2.33 ± 0.42
DOLK	A_23_P10870	0.91	1.11	2.32 ± 0.18
FAM86FP	A_33_P3339336	1.15	1.15	2.32 ± 0.34
LSM10	A_24_P216681	1.07	1.15	2.31 ± 0.24
F2	A_23_P94879	0.95	0.88	2.30 ± 0.18
ALG1	A_24_P586523	1.12	1.02	2.30 ± 0.26
SRSF2	A_33_P3412945	0.90	1.00	2.28 ± 0.33
CHAC1	A_33_P3376965	0.99	0.86	2.27 ± 0.40
LRRC31	A_24_P240259	0.71	0.89	2.26 ± 0.34
ANKRD42	A_33_P3224135	0.89	1.01	2.25 ± 0.43
TBC1D30	A_32_P206050	0.92	0.99	2.25 ± 0.71
KBTBD10	A_23_P17190	1.12	0.91	2.25 ± 0.59
CLSPN	A_23_P126212	1.09	0.94	2.24 ± 0.64
CHMP6	A_23_P10156	1.06	0.98	2.23 ± 0.39
TPM3	A_32_P119197	1.04	0.92	2.21 ± 0.49
WDR77	A_23_P115149	1.03	1.05	2.19 ± 0.12
DCTPP1	A_23_P33613	0.92	1.04	2.18 ± 0.33
NCRNA00292	A_33_P3299811	0.92	1.07	2.17 ± 0.39
ALKBH8	A_33_P3318357	0.99	1.03	2.17 ± 0.21
IKZF3	A_23_P376060	0.63	0.79	2.16 ± 0.66
POLA2	A_23_P161615	0.94	0.94	2.14 ± 0.36

NPL	A_33_P3316456	0.83	0.98	2.14 ± 0.34
NPY1R	A_23_P69699	1.06	0.90	2.13 ± 0.27
RFC3	A_23_P14193	0.97	1.06	2.13 ± 0.46
WDR5B	A_33_P3259548	0.88	1.04	2.08 ± 0.28
ZNF552	A_23_P38830	0.99	1.03	2.08 ± 0.13
DSCC1	A_23_P252740	0.95	0.90	2.02 ± 0.21
FEN1	A_24_P84898	1.00	0.95	2.02 ± 0.28
FAM86B1	A_33_P3628675	0.98	0.99	2.01 ± 0.31
FLJ30679	A_23_P346327	0.82	0.96	2.00 ± 0.66

ATP4A

GRCh38:chr19:35549443-35564658:-1

7 putative sites were predicted with these settings (95%) in sequence named **chromosome:GRCh38:19:35549443:35564658:-1**

Model ID	Model name	Score	Relative score	Start	End	Strand	predicted site sequence
MA0259.1	ARNT::HIF1A	10.987	0.993674439370933	544	551	-1	GCACGTGC
MA0259.1	ARNT::HIF1A	10.987	0.993674439370933	544	551	1	GCACGTGC
MA0259.1	ARNT::HIF1A	10.321	0.973806031368894	8723	8730	-1	CTACGTGC
MA0259.1	ARNT::HIF1A	10.184	0.969718986479586	10051	10058	1	ACACGTGC
MA0259.1	ARNT::HIF1A	10.987	0.993674439370933	12380	12387	-1	GCACGTGC
MA0259.1	ARNT::HIF1A	10.987	0.993674439370933	12380	12387	1	GCACGTGC
MA0259.1	ARNT::HIF1A	9.817	0.958770479367351	13501	13508	-1	GGACGTGA

Comment: This type of analysis has a high sensitivity but abysmal selectivity. In other words: while true functional will be detected in most cases, most predictions will correspond to sites bound in vitro but with no function in vivo. A number of additional contraints of the analysis can improve the prediction; phylogenetic footprinting is the most common. We recommend using the ConSite service, which uses the JASPAR datasets.

The review Nat Rev Genet. 2004 Apr;5(4):276-87 gives a comprehensive overview of transcription binding site prediction

PLA2G4D

GRCh38:chr15:42063009-42082008:-1

3 putative sites were predicted with these settings (95%) in sequence named **PLA2G4D_GRCh38:42063009:42082008:-1**

Model ID	Model name	Score	Relative score	Start	End	Strand	predicted site sequence
MA0259.1	ARNT::HIF1A	9.694	0.955101088700307	11044	11051	1	GTACGTGA
MA0259.1	ARNT::HIF1A	9.739	0.956443548700445	16508	16515	-1	GCACGTGG
MA0259.1	ARNT::HIF1A	10.232	0.971150943813066	16508	16515	1	CCACGTGC

Comment: This type of analysis has a high sensitivity but abysmal selectivity. In other words: while true functional will be detected in most cases, most predictions will correspond to sites bound in vitro but with no function in vivo. A number of additional contraints of the analysis can improve the prediction; phylogenetic footprinting is the most common. We recommend using the ConSite service, which uses the JASPAR datasets.

The review Nat Rev Genet. 2004 Apr;5(4):276-87 gives a comprehensive overview of transcription binding site prediction

GRCh38:chr15:42082009-42098554:-1

7 putative sites were predicted with these settings (95%) in sequence named PLA2G4D_GRCh38:15:42082009-42098554:-1							
Model ID	Model name	Score	Relative score	Start	End	Strand	predicted site sequence
MA0259.1	ARNT::HIF1A	9.817	0.958770479367351	3899	3906	1	GGACGTGA
MA0259.1	ARNT::HIF1A	10.232	0.971150943813066	6727	6734	-1	CCACGTGC
MA0259.1	ARNT::HIF1A	9.739	0.956443548700445	6727	6734	1	GCACGTGG
MA0259.1	ARNT::HIF1A	10.184	0.969718986479586	8863	8870	1	ACACGTGC
MA0259.1	ARNT::HIF1A	9.657	0.95399728825575	11047	11054	-1	GAACGTGC
MA0259.1	ARNT::HIF1A	10.184	0.969718986479586	13637	13644	-1	ACACGTGC
MA0259.1	ARNT::HIF1A	9.951	0.962768026923317	14182	14189	1	GGACGTGG

Comment: This type of analysis has a high sensitivity but abysmal selectivity. In other words: while true functional will be detected in most cases, most predictions will correspond to sites bound in vitro but with no function in vivo. A number of additional contraints of the analysis can improve the prediction; phylogenetic footprinting is the most common. We recommend using the ConSite service, which uses the JASPAR datasets.

The review Nat Rev Genet. 2004 Apr;5(4):276-87 gives a comprehensive overview of transcription binding site prediction

NIM1K

hg38:chr5:43190068-43209000:+1

3 putative sites were predicted with these settings (95%) in sequence named NIM1K_hg38:chr5:43190068-43209000:+1							
Model ID	Model name	Score	Relative score	Start	End	Strand	predicted site sequence
MA0259.1	ARNT::HIF1A	10.987	0.993674439370933	8988	8995	-1	GCACGTGC
MA0259.1	ARNT::HIF1A	10.987	0.993674439370933	8988	8995	1	GCACGTGC
MA0259.1	ARNT::HIF1A	11.075	0.996299694482314	14160	14167	-1	GTACGTGC

Comment: This type of analysis has a high sensitivity but abysmal selectivity. In other words: while true functional will be detected in most cases, most predictions will correspond to sites bound in vitro but with no function in vivo. A number of additional contraints of the analysis can improve the prediction; phylogenetic footprinting is the most common. We recommend using the ConSite service, which uses the JASPAR datasets.

The review Nat Rev Genet. 2004 Apr;5(4):276-87 gives a comprehensive overview of transcription binding site prediction

hg38:chr5:43,209,01-43,228,000:+1

0 putative sites were predicted with these settings (95%) in sequence named NIM1K_hg38:chr5:43209001:43228000:+1							
Model ID	Model name	Score	Relative score	Start	End	Strand	predicted site sequence

Comment: This type of analysis has a high sensitivity but abysmal selectivity. In other words: while true functional will be detected in most cases, most predictions will correspond to sites bound in vitro but with no function in vivo. A number of additional contraints of the analysis can improve the prediction; phylogenetic footprinting is the most common. We recommend using the ConSite service, which uses the JASPAR datasets.

The review Nat Rev Genet. 2004 Apr;5(4):276-87 gives a comprehensive overview of transcription binding site prediction

hg38:chr5:43228001-43247000:+1

3 putative sites were predicted with these settings (95%) in sequence named **NIM1K_hg38:chr5:43228001-43247000:+1**

Model ID	Model name	Score	Relative score	Start	End	Strand	predicted site sequence
MA0259.1	ARNT::HIF1A	10.321	0.973806031368894	2882	2889	1	CTACGTGC
MA0259.1	ARNT::HIF1A	9.828	0.959098636256273	3940	3947	-1	GTACGTGG
MA0259.1	ARNT::HIF1A	9.951	0.962768026923317	17317	17324	1	GGACGTGG

Comment: This type of analysis has a high sensitivity but abysmal selectivity. In other words: while true functional will be detected in most cases, most predictions will correspond to sites bound in vitro but with no function in vivo. A number of additional contraints of the analysis can improve the prediction; phylogenetic footprinting is the most common. We recommend using the ConSite service, which uses the JASPAR datasets.

The review Nat Rev Genet. 2004 Apr;5(4):276-87 gives a comprehensive overview of transcription binding site prediction

hg38:chr5:43247001-43266000:+1

0 putative sites were predicted with these settings (95%) in sequence named **NIM1K_hg38:chr5:43247001-43266000:+1**

Model ID	Model name	Score	Relative score	Start	End	Strand	predicted site sequence

Comment: This type of analysis has a high sensitivity but abysmal selectivity. In other words: while true functional will be detected in most cases, most predictions will correspond to sites bound in vitro but with no function in vivo. A number of additional contraints of the analysis can improve the prediction; phylogenetic footprinting is the most common. We recommend using the ConSite service, which uses the JASPAR datasets.

The review Nat Rev Genet. 2004 Apr;5(4):276-87 gives a comprehensive overview of transcription binding site prediction

hg38:chr5:43266001-43282850:+1

2 putative sites were predicted with these settings (95%) in sequence named **NIM1K_hg38:chr5:43266001-43282850:+1**

Model ID	Model name	Score	Relative score	Start	End	Strand	predicted site sequence
MA0259.1	ARNT::HIF1A	9.590	0.951998514477767	10481	10488	-1	GGACGTGT
MA0259.1	ARNT::HIF1A	10.184	0.969718986479586	14294	14301	-1	ACACGTGC

Comment: This type of analysis has a high sensitivity but abysmal selectivity. In other words: while true functional will be detected in most cases, most predictions will correspond to sites bound in vitro but with no function in vivo. A number of additional contraints of the analysis can improve the prediction; phylogenetic footprinting is the most common. We recommend using the ConSite service, which uses the JASPAR datasets.

The review Nat Rev Genet. 2004 Apr;5(4):276-87 gives a comprehensive overview of transcription binding site prediction

CYP26A1

hg38:chr10:93071890-93079890:+1

3 putative sites were predicted with these settings (95%) in sequence named **CYP26A1:hg38:chr10:93071890-93079890:+1**

Model ID	Model name	Score	Relative score	Start	End	Strand	predicted site sequence
MA0259.1	ARNT::HIF1A	9.817	0.958770479367351	73	80	-1	GGACGTGA
MA0259.1	ARNT::HIF1A	10.321	0.973806031368894	2928	2935	1	CTACGTGC
MA0259.1	ARNT::HIF1A	10.444	0.977475422035937	3153	3160	1	CGACGTGC

Comment: This type of analysis has a high sensitivity but abysmal selectivity. In other words: while true functional will be detected in most cases, most predictions will correspond to sites bound in vitro but with no function in vivo. A number of additional contraints of the analysis can improve the prediction; phylogenetic footprinting is the most common. We recommend using the ConSite service, which uses the JASPAR datasets.

The review Nat Rev Genet. 2004 Apr;5(4):276-87 gives a comprehensive overview of transcription binding site prediction

KISS1R

hg38:chr19:915342-923015:+1

11 putative sites were predicted with these settings (95%) in sequence named KISS1R_hg38:chr19:915342-923015:+1

Model ID	Model name	Score	Relative score	Start	End	Strand	predicted site sequence
MA0259.1	ARNT::HIF1A	9.817	0.958770479367351	3219	3226	1	GGACGTGA
MA0259.1	ARNT::HIF1A	9.828	0.959098636256273	3456	3463	1	GTACGTGG
MA0259.1	ARNT::HIF1A	10.232	0.971150943813066	3508	3515	-1	CCACGTGC
MA0259.1	ARNT::HIF1A	9.739	0.956443548700445	3508	3515	1	GCACGTGG
MA0259.1	ARNT::HIF1A	10.232	0.971150943813066	3607	3614	-1	CCACGTGC
MA0259.1	ARNT::HIF1A	9.739	0.956443548700445	3607	3614	1	GCACGTGG
MA0259.1	ARNT::HIF1A	9.694	0.955101088700307	4202	4209	1	GTACGTGA
MA0259.1	ARNT::HIF1A	9.542	0.950566557144287	4240	4247	-1	GGGCGTGC
MA0259.1	ARNT::HIF1A	9.590	0.951998514477767	4321	4328	-1	GGACGTGT
MA0259.1	ARNT::HIF1A	10.232	0.971150943813066	4476	4483	-1	CCACGTGC
MA0259.1	ARNT::HIF1A	9.739	0.956443548700445	4476	4483	1	GCACGTGG

Comment: This type of analysis has a high sensitivity but abysmal selectivity. In other words: while true functional will be detected in most cases, most predictions will correspond to sites bound in vitro but with no function in vivo. A number of additional contraints of the analysis can improve the prediction; phylogenetic footprinting is the most common. We recommend using the ConSite service, which uses the JASPAR datasets.

The review Nat Rev Genet. 2004 Apr;5(4):276-87 gives a comprehensive overview of transcription binding site prediction

GPR26

hg38:chr10:123664355-123683000:+1

5 putative sites were predicted with these settings (95%) in sequence named GPR26_hg38:chr10:123664355-123683000:+1

Model ID	Model name	Score	Relative score	Start	End	Strand	predicted site sequence
MA0259.1	ARNT::HIF1A	9.951	0.962768026923317	411	418	-1	GGACGTGG
MA0259.1	ARNT::HIF1A	9.605	0.952446001144479	2196	2203	-1	GCACGTGA
MA0259.1	ARNT::HIF1A	11.075	0.996299694482314	2622	2629	-1	GTACGTGC
MA0259.1	ARNT::HIF1A	10.184	0.969718986479586	3960	3967	-1	ACACGTGC
MA0259.1	ARNT::HIF1A	10.184	0.969718986479586	6350	6357	-1	ACACGTGC

Comment: This type of analysis has a high sensitivity but abysmal selectivity. In other words: while true functional will be detected in most cases, most predictions will correspond to sites bound in vitro but with no function in vivo. A number of additional contraints of the analysis can improve the prediction; phylogenetic footprinting is the most common. We recommend using the ConSite service, which uses the JASPAR datasets.

The review Nat Rev Genet. 2004 Apr;5(4):276-87 gives a comprehensive overview of transcription binding site prediction

hg38:chr10:123683001-123696607:+1

2 putative sites were predicted with these settings (95%) in sequence named **GPR26_hg38:chr10:123683001-123696607:+1**

Model ID	Model name	Score	Relative score	Start	End	Strand	predicted site sequence
MA0259.1	ARNT::HIF1A	10.184	0.969718986479586	1330	1337	1	ACACGTGC
MA0259.1	ARNT::HIF1A	9.951	0.962768026923317	2025	2032	-1	GGACGTGG

Comment: This type of analysis has a high sensitivity but abysmal selectivity. In other words: while true functional will be detected in most cases, most predictions will correspond to sites bound in vitro but with no function in vivo. A number of additional contraints of the analysis can improve the prediction; phylogenetic footprinting is the most common. We recommend using the ConSite service, which uses the JASPAR datasets.

The review Nat Rev Genet. 2004 Apr;5(4):276-87 gives a comprehensive overview of transcription binding site prediction

MYBPC2

hg38:chr19:50430903-50450000:+1

3 putative sites were predicted with these settings (95%) in sequence named **MYBPC2_hg38:chr19:50430903-50450000:+1**

Model ID	Model name	Score	Relative score	Start	End	Strand	predicted site sequence
MA0259.1	ARNT::HIF1A	9.951	0.962768026923317	5621	5628	1	GGACGTGG
MA0259.1	ARNT::HIF1A	10.232	0.971150943813066	18793	18800	-1	CCACGTGC
MA0259.1	ARNT::HIF1A	9.739	0.956443548700445	18793	18800	1	GCACGTGG

Comment: This type of analysis has a high sensitivity but abysmal selectivity. In other words: while true functional will be detected in most cases, most predictions will correspond to sites bound in vitro but with no function in vivo. A number of additional contraints of the analysis can improve the prediction; phylogenetic footprinting is the most common. We recommend using the ConSite service, which uses the JASPAR datasets.

The review Nat Rev Genet. 2004 Apr;5(4):276-87 gives a comprehensive overview of transcription binding site prediction

hg38:chr19:50450001-50468321:+1

6 putative sites were predicted with these settings (95%) in sequence named **MYBPC2_hg38:chr19:50450001-50468321:+1**

Model ID	Model name	Score	Relative score	Start	End	Strand	predicted site sequence
MA0259.1	ARNT::HIF1A	9.951	0.962768026923317	4148	4155	1	GGACGTGG
MA0259.1	ARNT::HIF1A	10.987	0.993674439370933	9173	9180	-1	GCACGTGC
MA0259.1	ARNT::HIF1A	10.987	0.993674439370933	9173	9180	1	GCACGTGC
MA0259.1	ARNT::HIF1A	9.605	0.952446001144479	10330	10337	1	GCACGTGA
MA0259.1	ARNT::HIF1A	10.987	0.993674439370933	10843	10850	-1	GCACGTGC
MA0259.1	ARNT::HIF1A	10.987	0.993674439370933	10843	10850	1	GCACGTGC

Comment: This type of analysis has a high sensitivity but abysmal selectivity. In other words: while true functional will be detected in most cases, most predictions will correspond to sites bound in vitro but with no function in vivo. A number of additional contraints of the analysis can improve the prediction; phylogenetic footprinting is the most common. We recommend using the ConSite service, which uses the JASPAR datasets.

The review Nat Rev Genet. 2004 Apr;5(4):276-87 gives a comprehensive overview of transcription binding site prediction

PCP4L1

hg38:chr1:161256727-161273000:+1

3 putative sites were predicted with these settings (95%) in sequence named **PCP4L1_hg38:chr1:161256727-161273000:+1**

Model ID	Model name	Score	Relative score	Start	End	Strand	predicted site sequence
MA0259.1	ARNT::HIF1A	9.590	0.951998514477767	2771	2778	1	GGACGTGT
MA0259.1	ARNT::HIF1A	10.987	0.993674439370933	14921	14928	-1	GCACGTGC
MA0259.1	ARNT::HIF1A	10.987	0.993674439370933	14921	14928	1	GCACGTGC

Comment: This type of analysis has a high sensitivity but abysmal selectivity. In other words: while true functional will be detected in most cases, most predictions will correspond to sites bound in vitro but with no function in vivo. A number of additional contraints of the analysis can improve the prediction; phylogenetic footprinting is the most common. We recommend using the ConSite service, which uses the JASPAR datasets.

The review Nat Rev Genet. 2004 Apr;5(4):276-87 gives a comprehensive overview of transcription binding site prediction

hg38:chr1:161273001-161287450:+1

0 putative sites were predicted with these settings (95%) in sequence named **PCP4L1_hg38:chr1:161273001-161287450:+1**

Model ID	Model name	Score	Relative score	Start	End	Strand	predicted site sequence

Comment: This type of analysis has a high sensitivity but abysmal selectivity. In other words: while true functional will be detected in most cases, most predictions will correspond to sites bound in vitro but with no function in vivo. A number of additional contraints of the analysis can improve the prediction; phylogenetic footprinting is the most common. We recommend using the ConSite service, which uses the JASPAR datasets.

The review Nat Rev Genet. 2004 Apr;5(4):276-87 gives a comprehensive overview of transcription binding site prediction

AMPD3

hg38:chr11:10453320-10472000:1+

1 putative sites were predicted with these settings (95%) in sequence named **AMPD3_hg38:chr11:10453320-10472000:1+**

Model ID	Model name	Score	Relative score	Start	End	Strand	predicted site sequence
MA0259.1	ARNT::HIF1A	9.657	0.95399728825575	2583	2590	-1	GAACGTGC

Comment: This type of analysis has a high sensitivity but abysmal selectivity. In other words: while true functional will be detected in most cases, most predictions will correspond to sites bound in vitro but with no function in vivo. A number of additional contraints of the analysis can improve the prediction; phylogenetic footprinting is the most common. We recommend using the ConSite service, which uses the JASPAR datasets.

The review Nat Rev Genet. 2004 Apr;5(4):276-87 gives a comprehensive overview of transcription binding site prediction

hg38:chr11:10472001-10490500:+1

3 putative sites were predicted with these settings (95%) in sequence named **AMPD3_hg38:chr11:10472001-10490500:+1**

Model ID	Model name	Score	Relative score	Start	End	Strand	predicted site sequence
MA0259.1	ARNT::HIF1A	10.987	0.993674439370933	13390	13397	-1	GCACGTGC
MA0259.1	ARNT::HIF1A	10.987	0.993674439370933	13390	13397	1	GCACGTGC
MA0259.1	ARNT::HIF1A	9.605	0.952446001144479	16429	16436	1	GCACGTGA

Comment: This type of analysis has a high sensitivity but abysmal selectivity. In other words: while true functional will be detected in most cases, most predictions will correspond to sites bound in vitro but with no function in vivo. A number of additional contraints of the analysis can improve the prediction; phylogenetic footprinting is the most common. We recommend using the ConSite service, which uses the JASPAR datasets.

The review Nat Rev Genet. 2004 Apr;5(4):276-87 gives a comprehensive overview of transcription binding site prediction

hg38:chr11:10490501-10509579:+1

3 putative sites were predicted with these settings (95%) in sequence named **AMPD3_hg38:chr11:10490501-10509579:+1**

Model ID	Model name	Score	Relative score	Start	End	Strand	predicted site sequence
MA0259.1	ARNT::HIF1A	9.657	0.95399728825575	4343	4350	1	GAACGTGC
MA0259.1	ARNT::HIF1A	10.184	0.969718986479586	10160	10167	1	ACACGTGC
MA0259.1	ARNT::HIF1A	9.657	0.95399728825575	10984	10991	-1	GAACGTGC

Comment: This type of analysis has a high sensitivity but abysmal selectivity. In other words: while true functional will be detected in most cases, most predictions will correspond to sites bound in vitro but with no function in vivo. A number of additional contraints of the analysis can improve the prediction; phylogenetic footprinting is the most common. We recommend using the ConSite service, which uses the JASPAR datasets.

The review Nat Rev Genet. 2004 Apr;5(4):276-87 gives a comprehensive overview of transcription binding site prediction

SCNN1G

hg38:chr16:23180715-23199500:+1

1 putative sites were predicted with these settings (95%) in sequence named **SCNN1G_hg38:chr16:23180715-23199500:+1**

Model ID	Model name	Score	Relative score	Start	End	Strand	predicted site sequence
MA0259.1	ARNT::HIF1A	9.542	0.950566557144287	5513	5520	-1	GGGCGTGC

Comment: This type of analysis has a high sensitivity but abysmal selectivity. In other words: while true functional will be detected in most cases, most predictions will correspond to sites bound in vitro but with no function in vivo. A number of additional contraints of the analysis can improve the prediction; phylogenetic footprinting is the most common. We recommend using the ConSite service, which uses the JASPAR datasets.

The review Nat Rev Genet. 2004 Apr;5(4):276-87 gives a comprehensive overview of transcription binding site prediction

hg38:chr16:23199501-23218803:1+

5 putative sites were predicted with these settings (95%) in sequence named **SCNN1G_hg38:chr16:23199501-23218803:1+**

Model ID	Model name	Score	Relative score	Start	End	Strand	predicted site sequence
MA0259.1	ARNT::HIF1A	9.951	0.962768026923317	3823	3830	1	GGACGTGG
MA0259.1	ARNT::HIF1A	10.232	0.971150943813066	8501	8508	-1	CCACGTGC
MA0259.1	ARNT::HIF1A	9.739	0.956443548700445	8501	8508	1	GCACGTGG
MA0259.1	ARNT::HIF1A	9.951	0.962768026923317	9428	9435	-1	GGACGTGG
MA0259.1	ARNT::HIF1A	9.542	0.950566557144287	18379	18386	1	GGGCGTGC

Comment: This type of analysis has a high sensitivity but abysmal selectivity. In other words: while true functional will be detected in most cases, most predictions will correspond to sites bound in vitro but with no function in vivo. A number of additional contraints of the analysis can improve the prediction; phylogenetic footprinting is the most common. We recommend using the ConSite service, which uses the JASPAR datasets.

The review Nat Rev Genet. 2004 Apr;5(4):276-87 gives a comprehensive overview of transcription binding site prediction

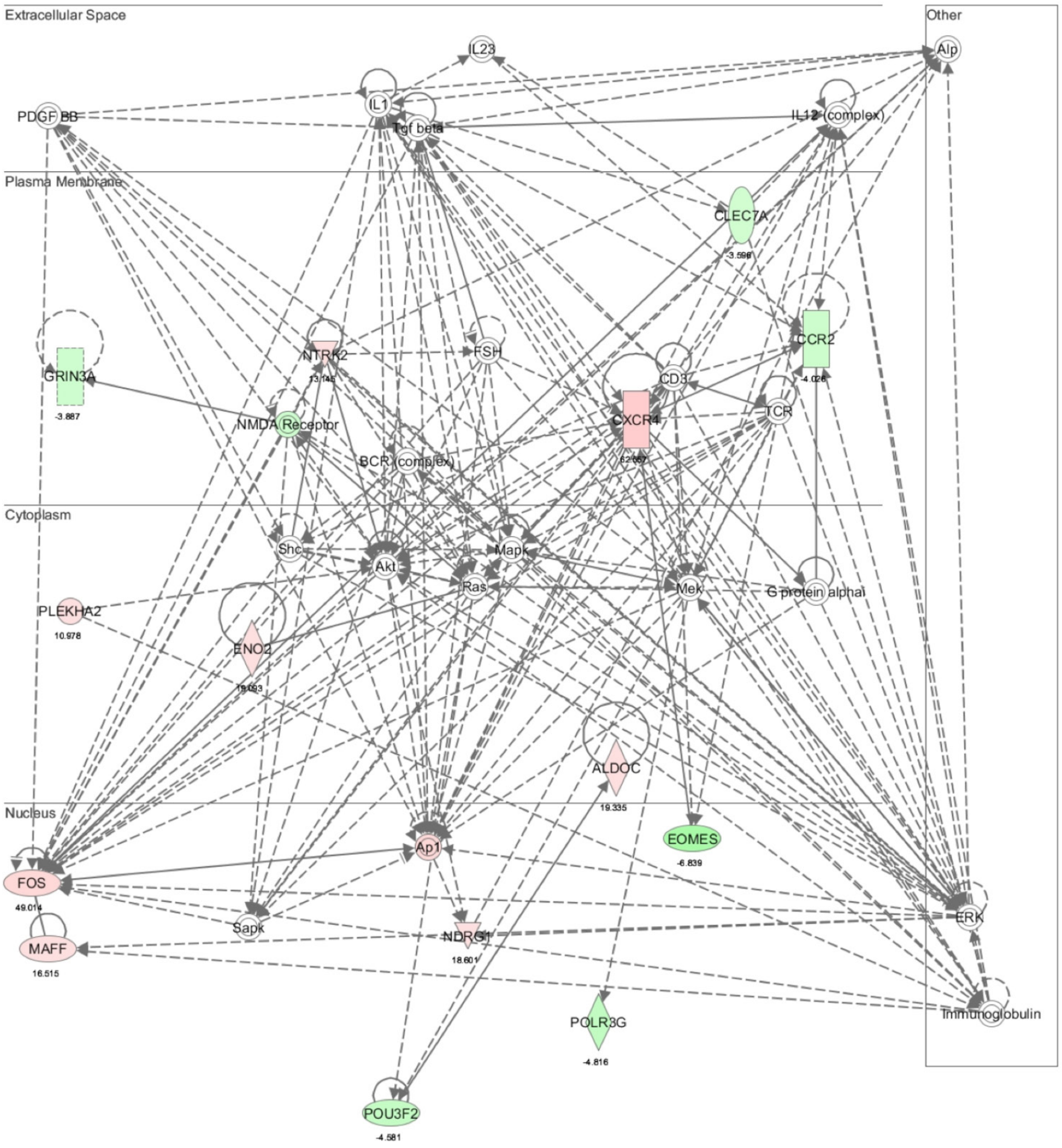

Supplementary Figure S1: Cellular movement, hematological system development and function, immune cell trafficking network. A network identified after Ingenuity Pathway Assist analysis of the top 50 up-regulated genes and top 50 down-regulated genes that are most sensitive to Rb-loss and hypoxia from the shRNA LNCaP microarray.

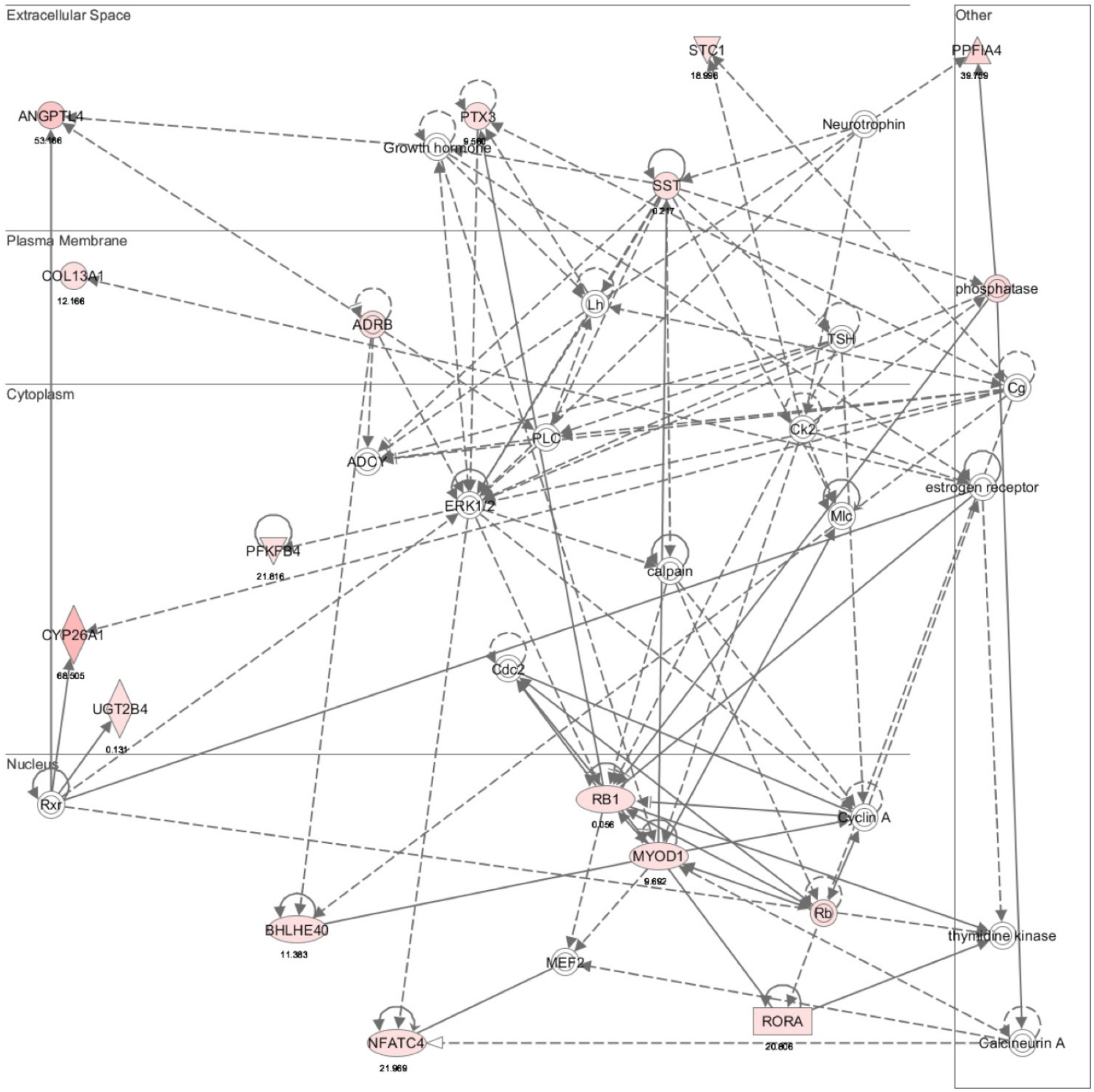

Supplementary Figure S2: Cardiovascular system organismal development, skeletal and muscular system network. A network identified after Ingenuity Pathway Assist analysis of the top 50 up-regulated genes and top 50 down-regulated genes that are most sensitive to Rb-loss and hypoxia from the shRNA LNCaP microarray.

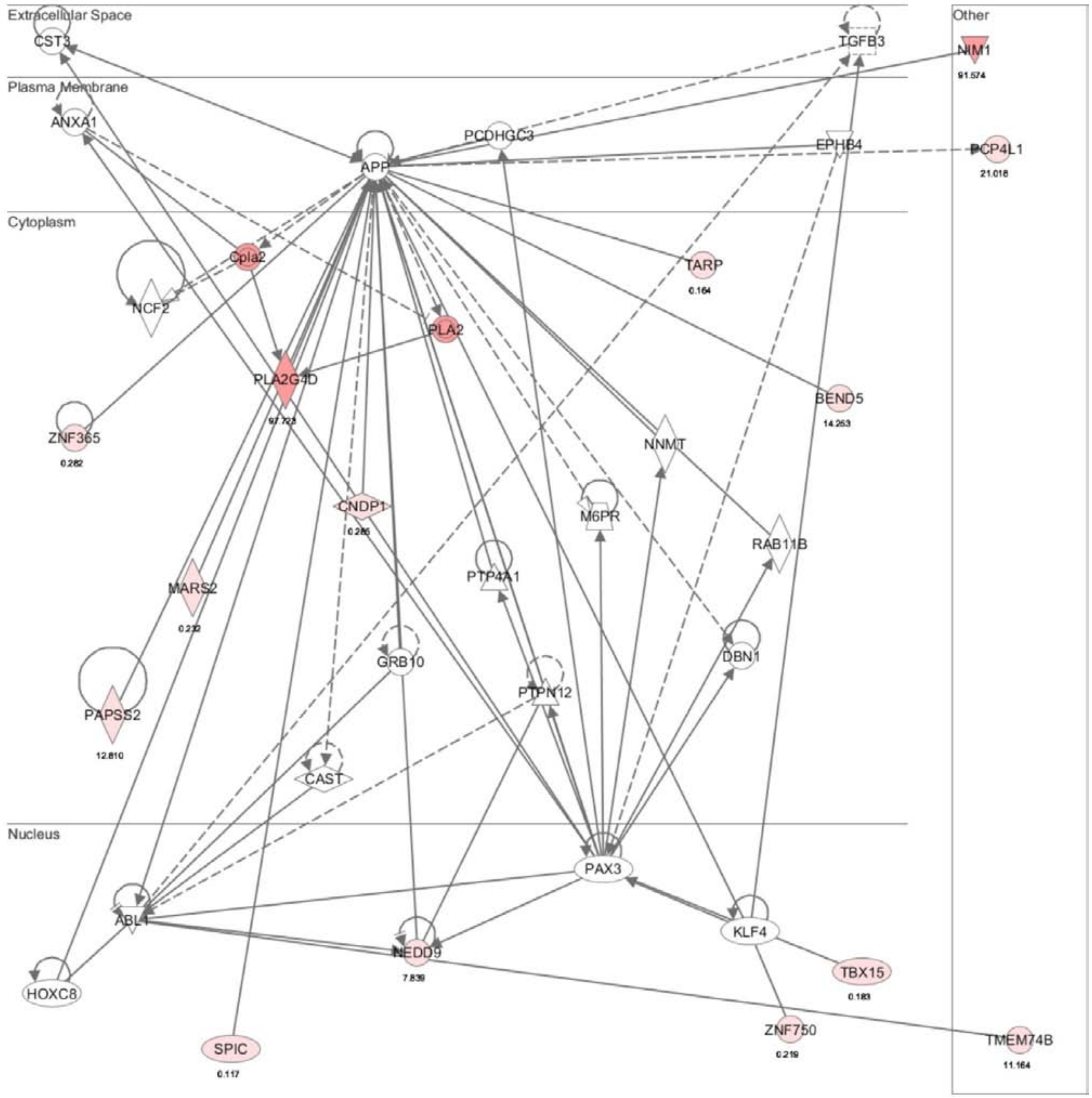

Supplementary Figure S3: Cardiovascular disease developmental disorder, hereditary disorder network. A network identified after Ingenuity Pathway Assist analysis of the top 50 up-regulated genes and top 50 down-regulated genes that are most sensitive to Rb-loss and hypoxia from the shRNA LNCaP microarray.

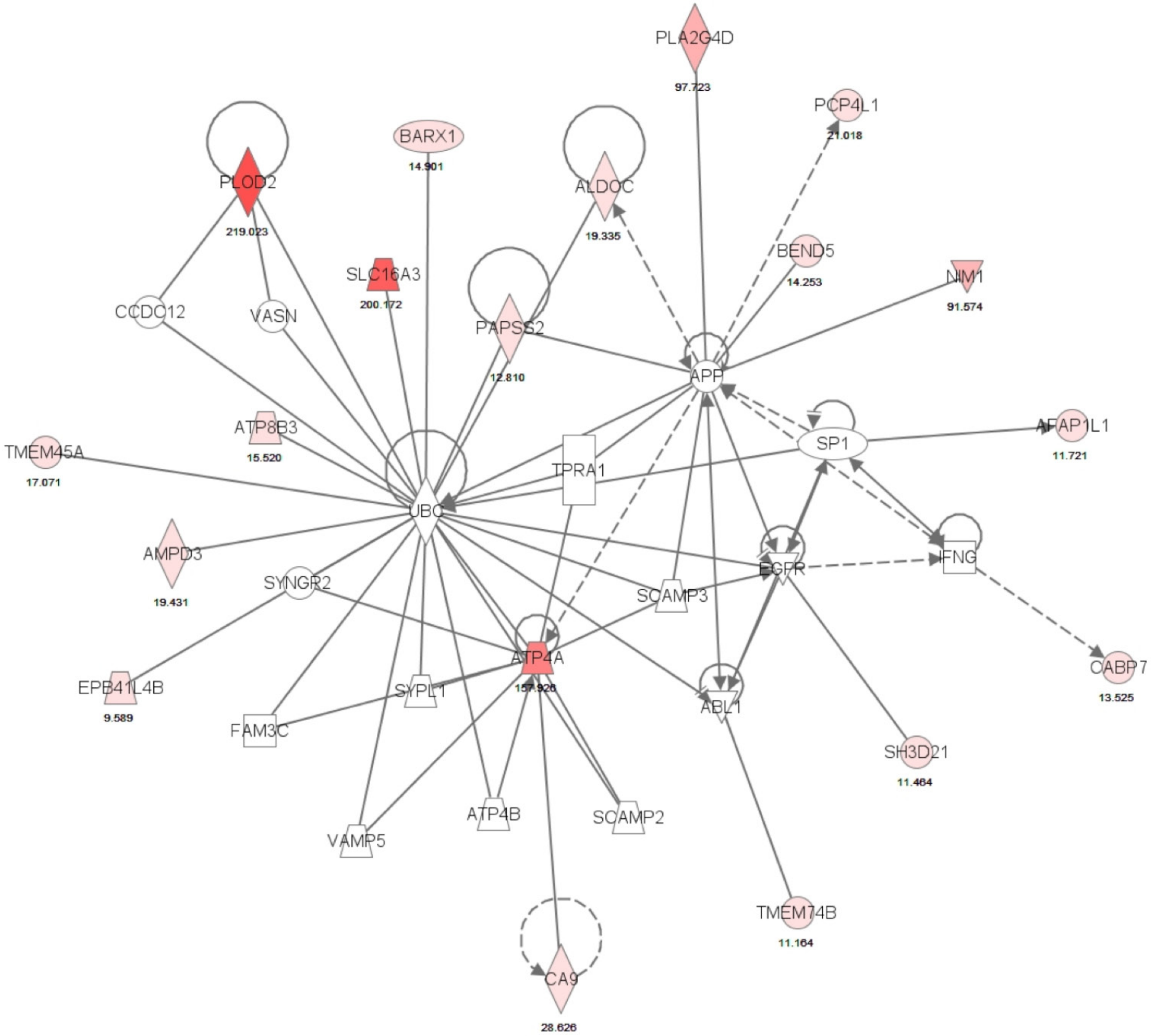

Supplementary Figure S4: Cardiac arteriopathy, cardiovascular disease, hematological disease network. A network identified after Ingenuity Pathway Assist analysis of the top 50 up-regulated genes and top 50 down-regulated genes that are most sensitive to Rb-loss and hypoxia from the shRNA LNCaP microarray.

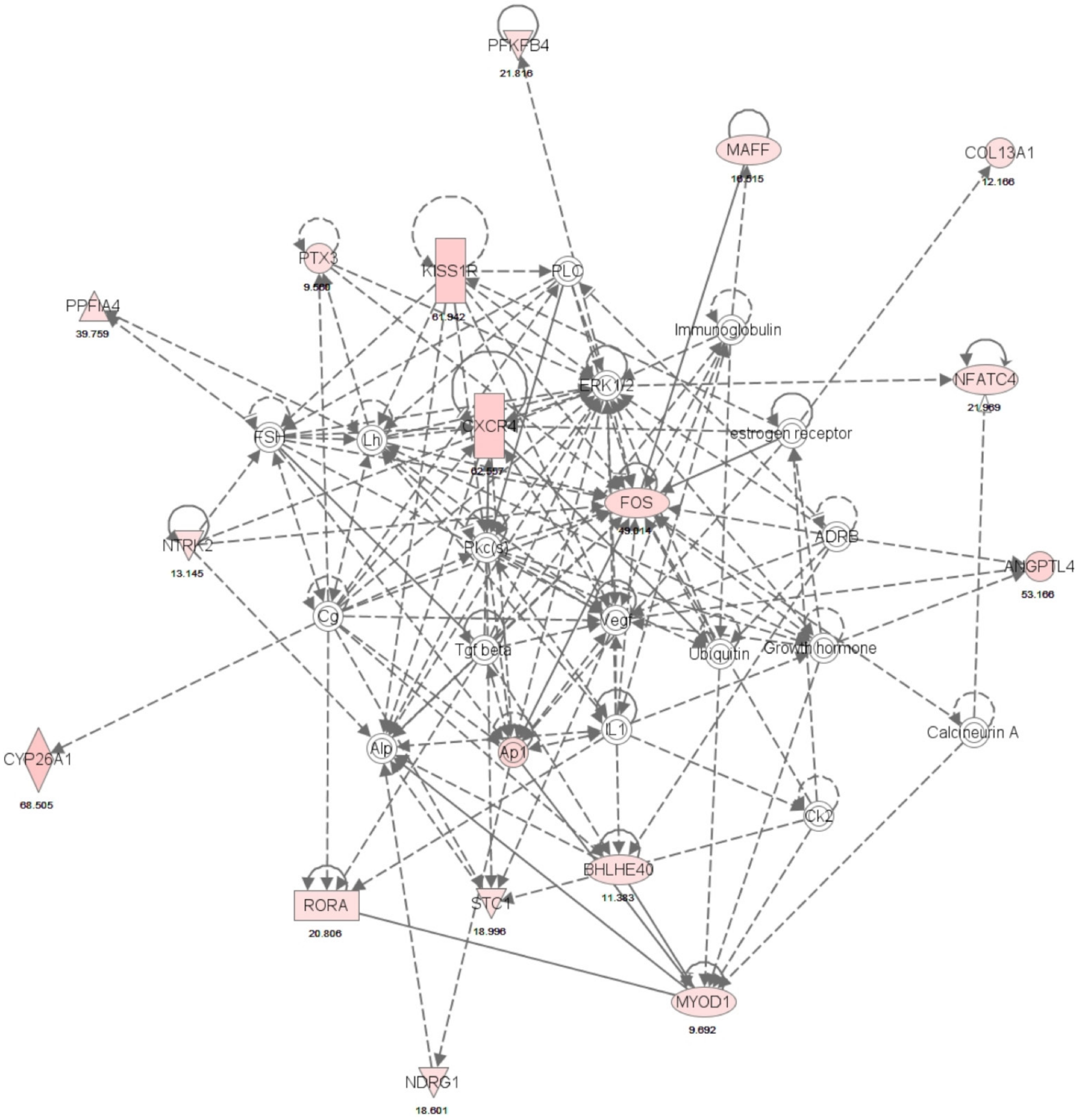

Supplementary Figure S5: Organ morphology, organismal development, reproductive system development and function network. A network identified after Ingenuity Pathway Assist analysis of the top 50 up-regulated genes and top 50 down-regulated genes that are most sensitive to Rb-loss and hypoxia from the shRNA LNCaP microarray.

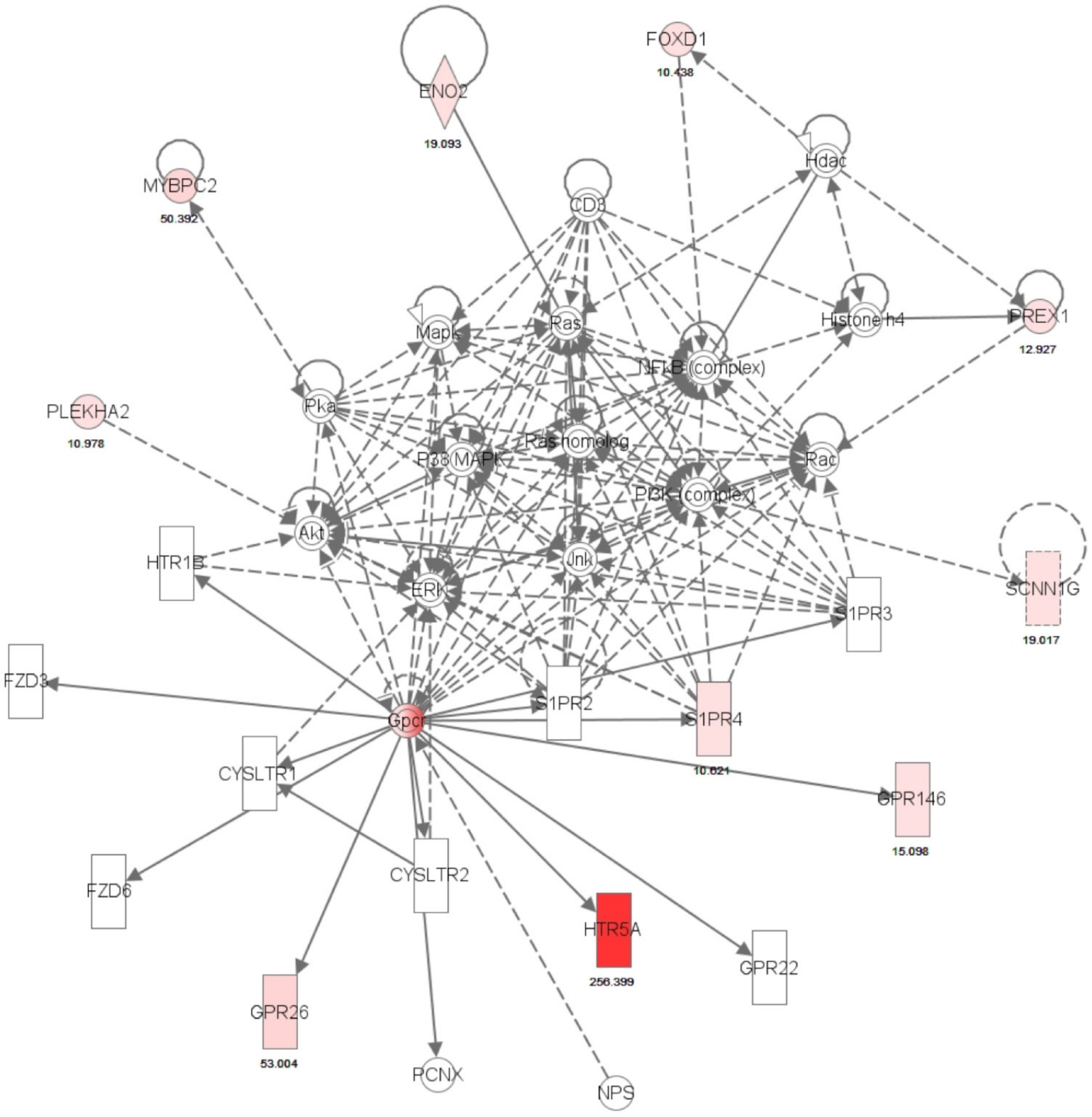

Supplementary Figure S6: Cellular development, cellular growth and proliferation, hematological system development and function network. A network identified after Ingenuity Pathway Assist analysis of the top 50 up-regulated genes and top 50 down-regulated genes that are most sensitive to Rb-loss and hypoxia from the shRNA LNCaP microarray.

MATERIALS AND METHODS

Transient transfections

LNCaP and shRb LNCaP cells were transfected with 10–15 nM of either scrambled (siSCX) siRNA or DP1 siRNAs (siDP1-i and siDP1-ii) using 0.3% (v/v) Lipofectamine RNAiMAX (Invitrogen Inc) according to manufacturer's protocol. The cells were allowed to incubate in transfection mix for 6 h at 37°C, 20% O_2, and 5% CO_2 after which the transfection mix was removed and replaced with complete media.

Slide preparation and hypoxia treatment

18 mm round slides were soaked in 1 N Nitric Acid overnight, then washed two- three times with mili-Q water and stored in 70% Ethanol. Before use, the slides were washed twice with sterile PBS and coated with polyethylenamine for 30 min at 37°C. LNCaP shSCX and shRb cells were seeded at 62,500 cells/slide on 18 mm round glass slides in a 12-well plate. One of the plates was placed into a hypoxia chamber set at 37°C, 1% O_2, and 5% CO_2, while the other plate was left at 37°C, 20% O_2, and 5% CO_2 for 96 h.

Immunostaining

Following 96 h of hypoxia or normoxia the slides were washed two times with ice cold PBS, and fixed with ice cold Methanol at –20°C for 10 min. The slides were then washed two times with ice cold PBS and blocked in 5% Normal Donkey Serum (Jackson ImmunoResearch Laboratories) and 0.3 M Glycine in PBS for 30 min at room temperature. Next, the slides were incubated with either PBS or KISS1R primary antibody (1/100 dilution; abcam; ab140839) overnight at 4°C. Following incubation, the slides were washed two times with ice cold PBS for 5 min at room temperature. The slides were then incubated with Alexa Flour® 680 donkey anti-rabbit IgG (1/1000, Life technologies; A10043) in PBS for 1 h at room temperature, then washed two times with ice cold PBS for 5 min. Slides were stained with Hoescht (1/10,000) in PBS for 1 min, followed by two washes of ice cold PBS. Lastly, the slides were mounted using FlourSave (Calbiochem), and imaged at 60×using water and the Metamorph software. *Number of Foci/cell was counted using the Metamorph software. (using a threshold of 450 and dot range of 10–120)*.

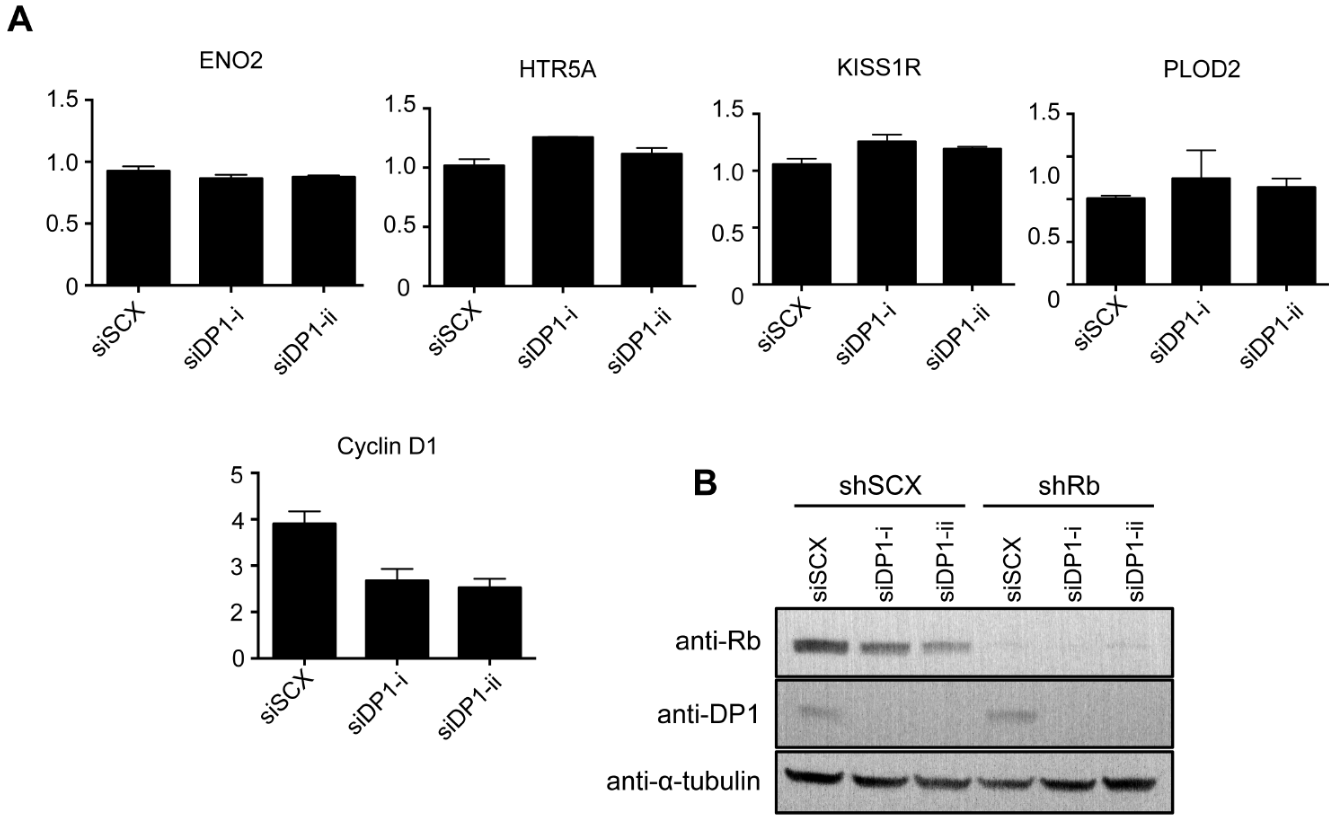

Supplementary Figure S7: siRNA-mediated suppression of DP1 expression in shRb LNCaP cells does not affect HIF1-regulated transcription of identified array genes. (**A**) shRb LNCaP cells were transfected with either scrambled siRNA (siSCX) or DPI siRNAs (siDP1-i and siDP1-ii). Twenty-four hours after transfection cells were treated with 1% O_2 for a further 24 h. Gene expression was determined by quantitative real-time PCR after isolation and reverse transcription of total RNA. Target gene expression was normalized to constitutively active 36B4 gene expression. Cyclin D1 expression was used as a positive control as it is directly regulated by E2F-DP1 transcription factors. Error bars represent ± S.D. (**B**) Immunoblot of Rb, DP1 and α-tubulin. shRNA LNCaP cells were transfected with either scrambled control (siSCX) or two siRNA directed to DP1. Forty-eight hours after transfection, whole cell lysates were collected, analyzed and fractionated by SDS-PAGE. Alpha-tubulin (α-tubulin) was used as a loading control.

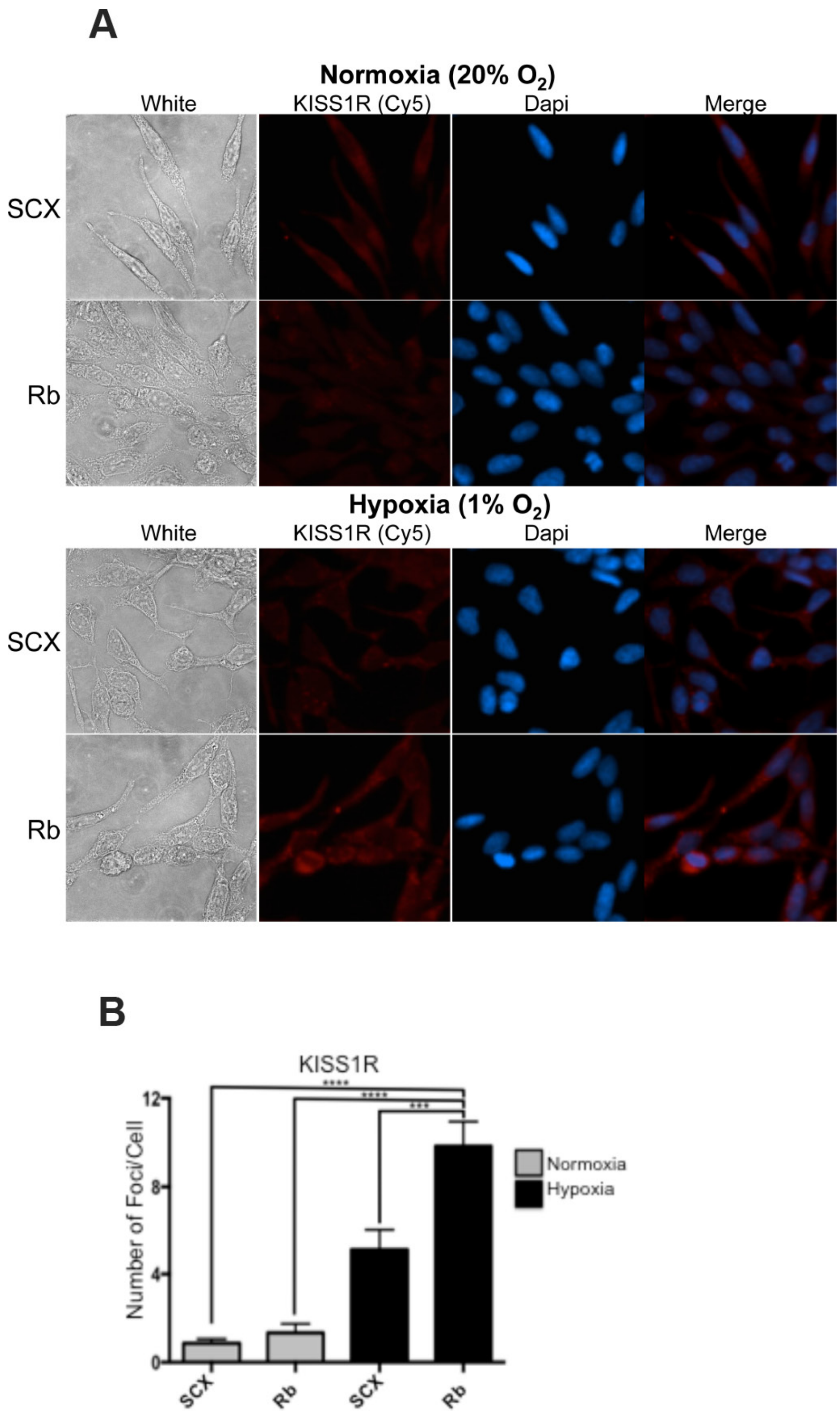

Supplementary Figure S8: Cytoplasmic and membrane KISS1R protein expression is increased in Rb-ablated LNCaP cells exposed to hypoxia. (**A**) Immunocytochemistry of shRNA LNCaP cells treated with either normoxia or hypoxia for 96 hours. Cells were stained with KISS1R primary antibody and Hoescht and then imaged on a fluorescent microscope at 60 × using water and the Metamorph software. (**B**) Number of Foci per cell was counted using the Metamorph software and a threshold of 450 and dot range of 10–120. Error bars represent ± S.D. ****p* < 0.05.

Supplementary Data File 2

Gene Name	Gene Start (bp)	Gene End (bp)	Chromosome
RBP7	10068313	10075879	1
DHRS3	12627939	12677737	1
PADI2	17393256	17445948	1
IGSF21	18434240	18704977	1
RHCE	25696961	25697020	1
MAN1C1	26110835	26110894	1
HPCA	33351595	33364042	1
ADC	33563692	33563751	1
EIF2C4	36320420	36320479	1
SH3D21	36771988	36790484	1
RLF	40627045	40706593	1
RIMKLA	42846466	42888249	1
SLC2A1	43391052	43424530	1
FLJ32224	43441758	43441817	1
PTPRF	44044897	44044956	1
BEST4	45249257	45253377	1
ZSWIM5	45482071	45771881	1
TESK2	45809834	45809893	1
PDZK1IP1	47650675	47650734	1
BEND5	49193195	49242641	1
LDLRAD1	54474412	54483856	1
PRKAA2	57110995	57181008	1
JUN	59246465	59249785	1
GFI1	92940780	92940838	1
FAM69A	93307755	93307814	1
NTNG1	108024337	108024396	1
KCND3	112319817	112319876	1
RSBN1	114304454	114355098	1
TSPAN2	115591008	115591067	1
CTSK	150768853	150768912	1
S100A10	151955391	151966866	1
EFNA3	155036224	155060014	1
HCN3	155259312	155259371	1
RIT1	155867599	155881195	1
PCP4L1	161228517	161255240	1
GPA33	167022073	167059868	1
ATP1B1	169101877	169101936	1
PPFIA4	202995626	203047868	1
RASSF5	206762349	206762408	1
FAM177B	222923479	222923538	1
DUSP5P1	228744885	228788150	1
SLC35F3	234040679	234460262	1
ERO1LB	236380487	236380546	1
LGALS8	236681300	236716281	1
GREM2	240652873	240775449	1
SFPQ	35641979	35658749	1
NCDN	36023074	36032875	1
CLSPN	36185819	36235568	1
LSM10	36856839	36863493	1
TOE1	45805342	45809647	1
KTI12	52497775	52499488	1
INSL5	67263424	67266939	1
WDR77	111982512	111991998	1
AP4B1	114437370	114447823	1
TBX15	119425669	119532179	1
TPM3	154127784	154167124	1
MSTO1	155579979	155718153	1
GPATCH4	156564279	156571288	1
CYCSP52	157098154	157098463	1
CD244	160799950	160832692	1
TOR3A	179050512	179067158	1
NPL	182758428	182799519	1
GLRX2	193065598	193075244	1
SLC30A1	211744910	211752084	1
NCRNA00292	213031201	213031260	1
JMJD4	227918126	227923112	1

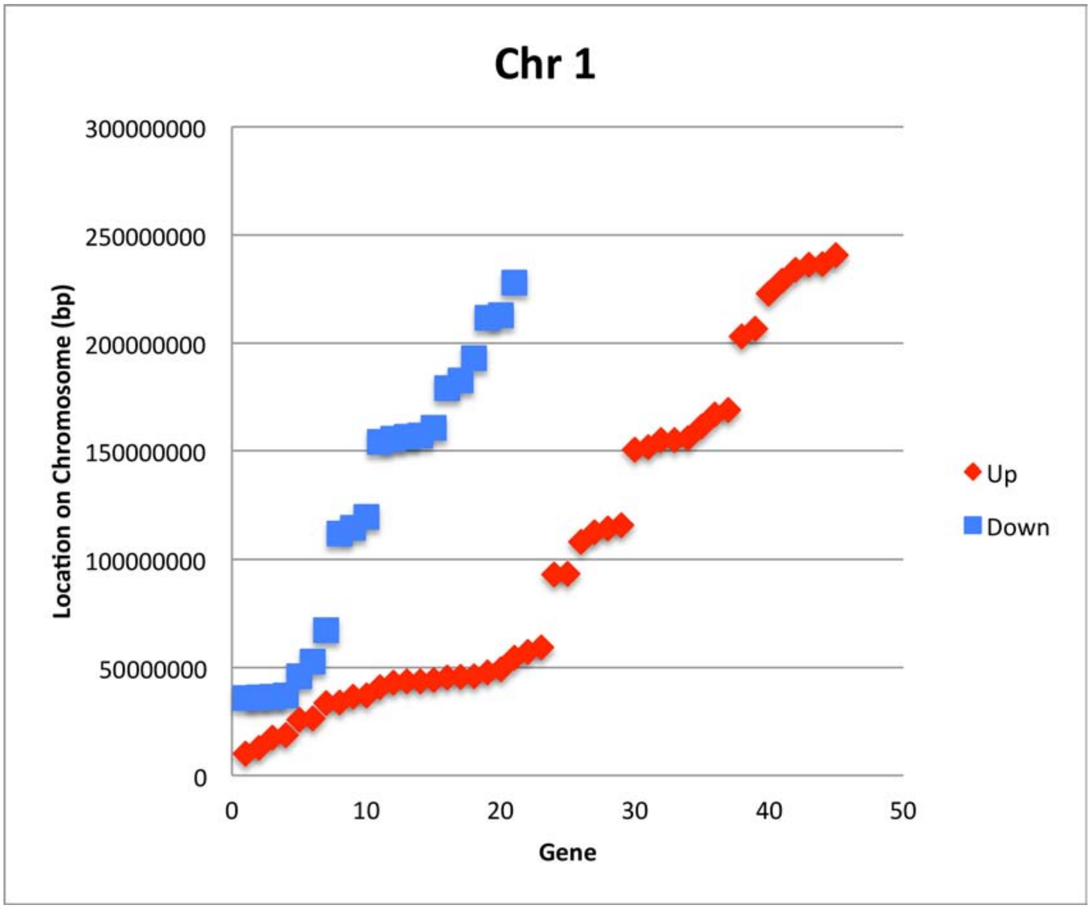
Chr 1
300000000
250000000
200000000
150000000
100000000
50000000
0
Location on Chromosome (bp)
0
10
20
30
40
50
Gene
Up
Down

Gene Name	Gene Start (bp)	Gene End (bp)	Chromosome	Gene Name	Gene Start (bp)	Gene End (bp)	Chromosome
FAM110C	38814	46870	2	MSGN1	17997763	17998368	2
CYS1	10196907	10221071	2	SLC5A6	27422455	27435826	2
SLC30A3	27477548	27477607	2	SRSF7	38970741	38978636	2
FLJ31356	28609783	28609842	2	CHAC2	53994929	54002333	2
FOSL2	28615315	28640179	2	PNO1	68384976	68403370	2
YPEL5	30369807	30383399	2	LRRTM4	76974845	77820445	2
CAPN14	31396201	31396260	2	POLR1B	113299492	113334635	2
CYP1B1	38294116	38337044	2	TMEM177	120436743	120444083	2
EPAS1	46520806	46613836	2	MKI67IP	122484856	122484915	2
STON1	48756522	48826025	2	KBTBD10	170382120	170382179	2
STON1-GTF2A	48757064	49003654	2	MARS2	198570087	198573113	2
KDM3A	86718371	86719192	2	AP1S3	224616403	224702744	2
NPAS2	101612199	101612258	2	GBX2	237073879	237077012	2
CREG2	101962013	102004057	2				
ZC3H6	113033171	113097640	2				
IL1RN	113891531	113891590	2				
INSIG2	118846028	118868573	2				
CXCR4	136871919	136875735	2				
CSRNP3	166536589	166536648	2				
GAD1	171669723	171717661	2				
CYBRD1	172378757	172414643	2				
PDK1	173420101	173489823	2				
GPR155	175296966	175351822	2				
KIAA1715	176788620	176867567	2				
FZD7	202899310	202903160	2				
FAM117B	203499901	203634480	2				
ANKZF1	220094479	220101391	2				
IRS1	227596033	227664475	2				
NGEF	233759328	233759387	2				
ARL4C	235401685	235405697	2				

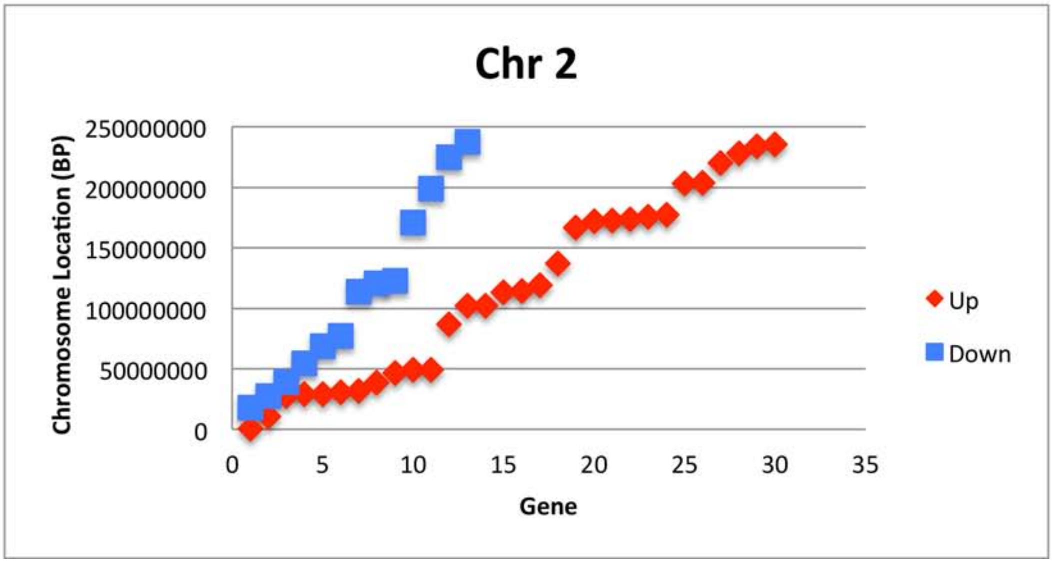

Gene Name	Gene Start (bp)	Gene End (bp)	Chromosome
BHLHE40	5020801	5027008	3
ZCWPW2	28566492	28566551	3
DLEC1	38080696	38165516	3
CLEC3B	45043040	45077563	3
PTH1R	46919236	46945287	3
PFKFB4	48555117	48599448	3
LAMB2P1	49190292	49190351	3
MST1	49721380	49726934	3
STAB1	52529354	52558511	3
GBE1	81538850	81811312	3
ST3GAL6	98451080	98540045	3
TMEM45A	100211463	100296288	3
ZBTB20	114056941	114866118	3
ABTB1	127391778	127399768	3
GATA2	128198270	128212028	3
PLXND1	129274018	129325661	3
RAB6B	133543083	133614680	3
PLOD2	145787227	145881440	3
PTX3	157154578	157161417	3
SCHIP1	159615048	159615107	3
BCHE	165490819	165490878	3
NLGN1	173114074	174004434	3
KLHL24	183353356	183402265	3
YEATS2	183415606	183530413	3
CHRD	184107493	184107552	3
BCL6	187439641	187439700	3
HES1	193853934	193856521	3

Gene Name	Gene Start (bp)	Gene End (bp)	Chromosome
OXNAD1	16306706	16391806	3
SGOL1	20202085	20227784	3
EOMES	27757440	27764206	3
NRADDP	47053032	47054636	3
CDC25A	48198636	48229892	3
SLC25A20	48894369	48936426	3
RRP9	51967446	51975957	3
KBTBD8	67048727	67061634	3
HGD	120347020	120401418	3
WDR5B	122130715	122134882	3
SRPRB	133502877	133544616	3
TM4SF18	149036285	149052201	3
LRRC31	169556967	169587718	3

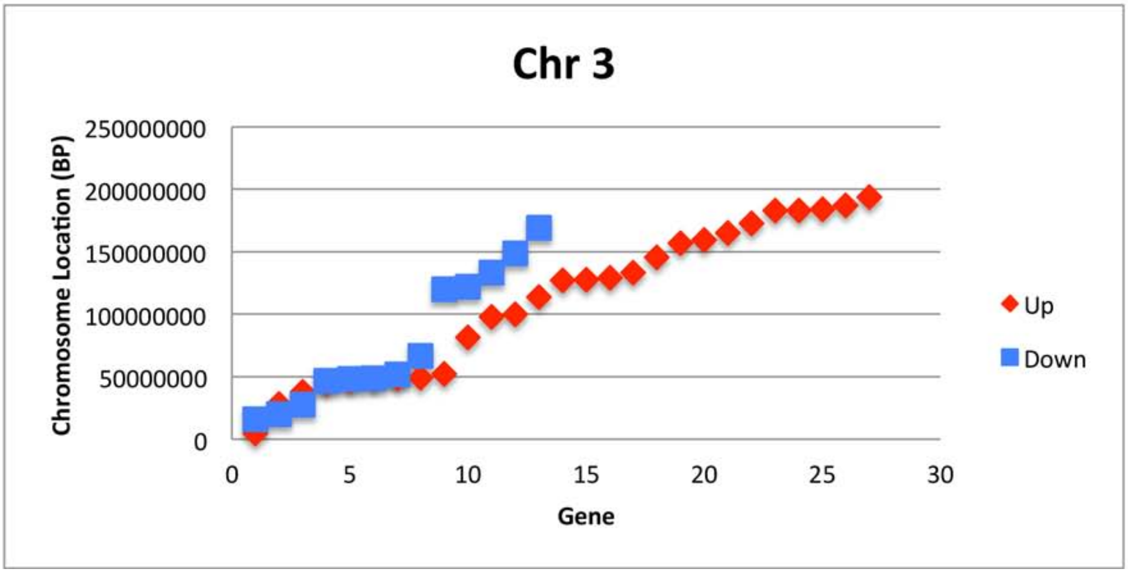

Gene Name	Gene Start (bp)	Gene End (bp)	Chromosome	Gene Name	Gene Start (bp)	Gene End (bp)	Chromosome
ODAM	71062213	71070293	4	STIM2	26859300	27027003	4
STBD1	77231410	77231469	4	KLB	39408473	39453156	4
CCNG2	78078304	78354542	4	UGT2B4	70345883	70391732	4
SCD5	83551061	83551120	4	AREG	75310851	75320726	4
HERC3	89442199	89629693	4	AGPAT9	84457067	84527028	4
FAM13A	89647106	90032549	4	MAPK10	86936276	87515284	4
NHEDC1	103822280	103822339	4	TRAM1L1	118004718	118006736	4
TET2	106163842	106163901	4	NPY1R	164245113	164265984	4
NPNT	106892414	106892473	4				
SPRY1	124317950	124324910	4				
GAB1	144390452	144390511	4				
TTC29	147724821	147741319	4				
LRAT	155548097	155674270	4				
SCRG1	174309459	174309518	4				
ANKRD37	186317175	186321782	4				
CCDC110	186366336	186392913	4				
PDLIM3	186423203	186423262	4				

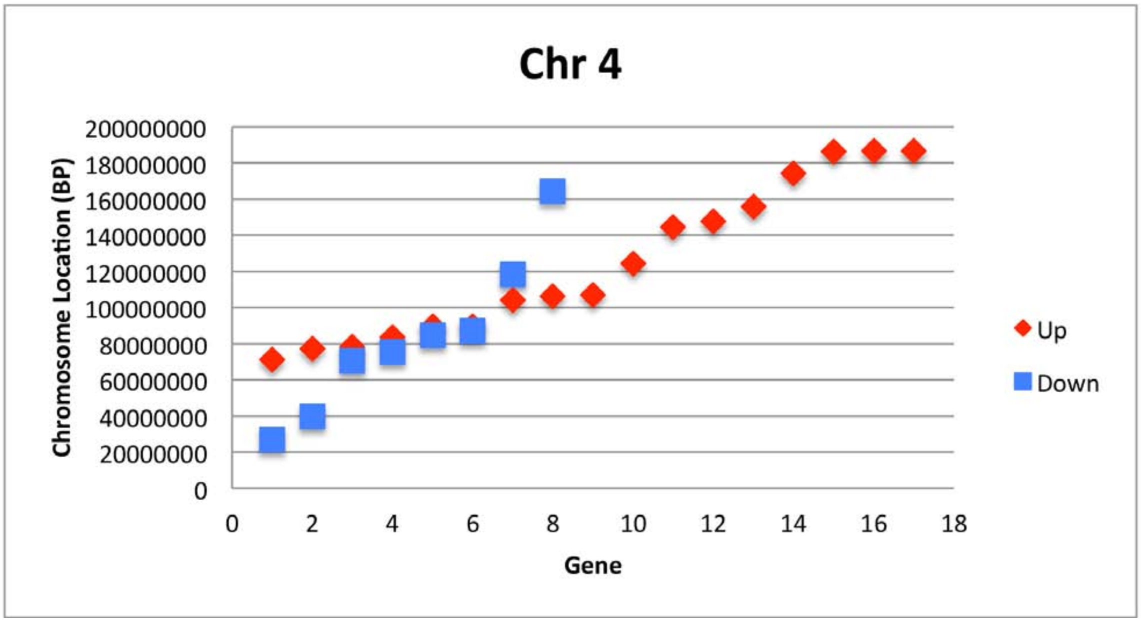

Gene Name	Gene Start (bp)	Gene End (bp)	Chromosome	Gene Name	Gene Start (bp)	Gene End (bp)	Chromosome
ODAM	71062213	71070293	4	STIM2	26859300	27027003	4
STBD1	77231410	77231469	4	KLB	39408473	39453156	4
CCNG2	78078304	78354542	4	UGT2B4	70345883	70391732	4
SCD5	83551061	83551120	4	AREG	75310851	75320726	4
HERC3	89442199	89629693	4	AGPAT9	84457067	84527028	4
FAM13A	89647106	90032549	4	MAPK10	86936276	87515284	4
NHEDC1	103822280	103822339	4	TRAM1L1	118004718	118006736	4
TET2	106163842	106163901	4	NPY1R	164245113	164265984	4
NPNT	106892414	106892473	4				
SPRY1	124317950	124324910	4				
GAB1	144390452	144390511	4				
TTC29	147724821	147741319	4				
LRAT	155548097	155674270	4				
SCRG1	174309459	174309518	4				
ANKRD37	186317175	186321782	4				
CCDC110	186366336	186392913	4				
PDLIM3	186423203	186423262	4				

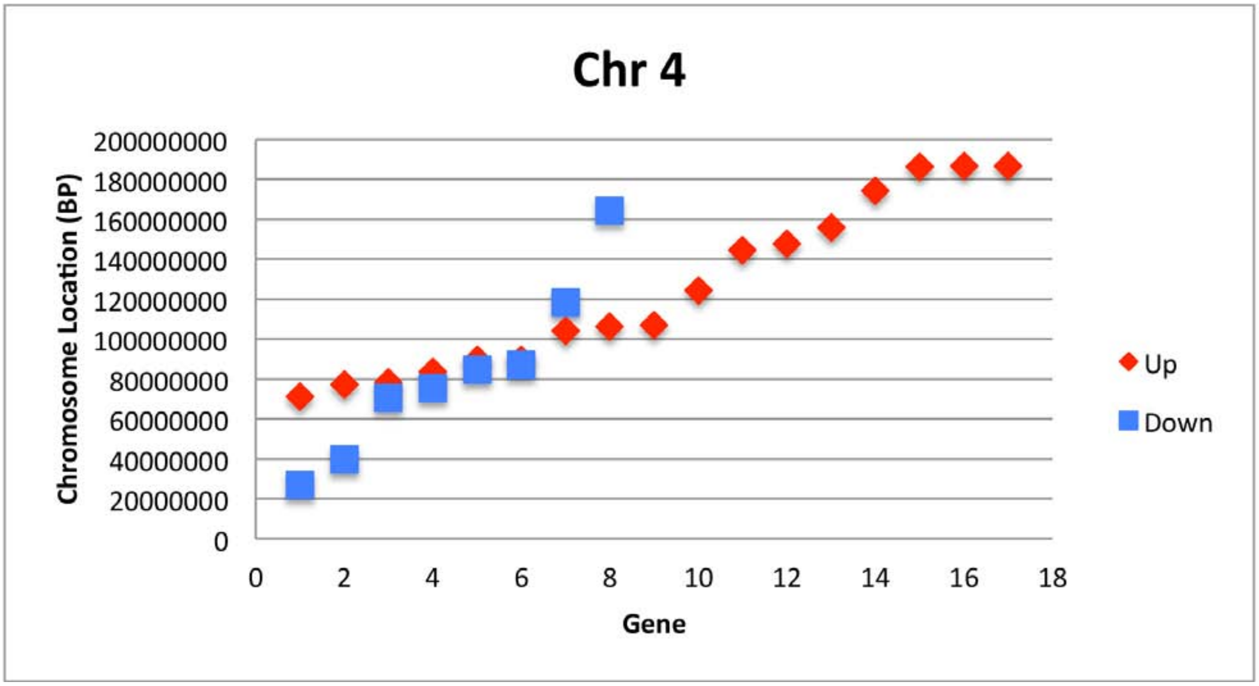

Gene Name	Gene Start (bp)	Gene End (bp)	Chromosome	Gene Name	Gene Start (bp)	Gene End (bp)	Chromosome
NPR3	32689176	32791819	5	POLR3G	89767565	89810370	5
C1QTNF3	34020604	34020663	5				
LIFR	38475942	38476001	5				
PTGER4	40679600	40693837	5				
NIM1	43280703	43280762	5				
PDE4D	58264927	58264986	5				
FOXD1	72740654	72744352	5				
DMGDH	78293438	78531861	5				
EPB41L4A	111478138	111755013	5				
LOX	121398890	121413980	5				
P4HA2	131527531	131631008	5				
CDKL3	133541305	133706738	5				
TGFBI	135364584	135399507	5				
KLHL3	136953189	137071779	5				
CXXC5	139062536	139062595	5				
ARAP3	141032968	141061788	5				
AFAP1L1	148651434	148721365	5				
ARHGEF37	148931510	149014531	5				
SYNPO	149980642	150038782	5				
TNIP1	150409506	150473138	5				
DUSP1	172195093	172198198	5				
STC2	172741716	172756506	5				
GFPT2	179727767	179727826	5				

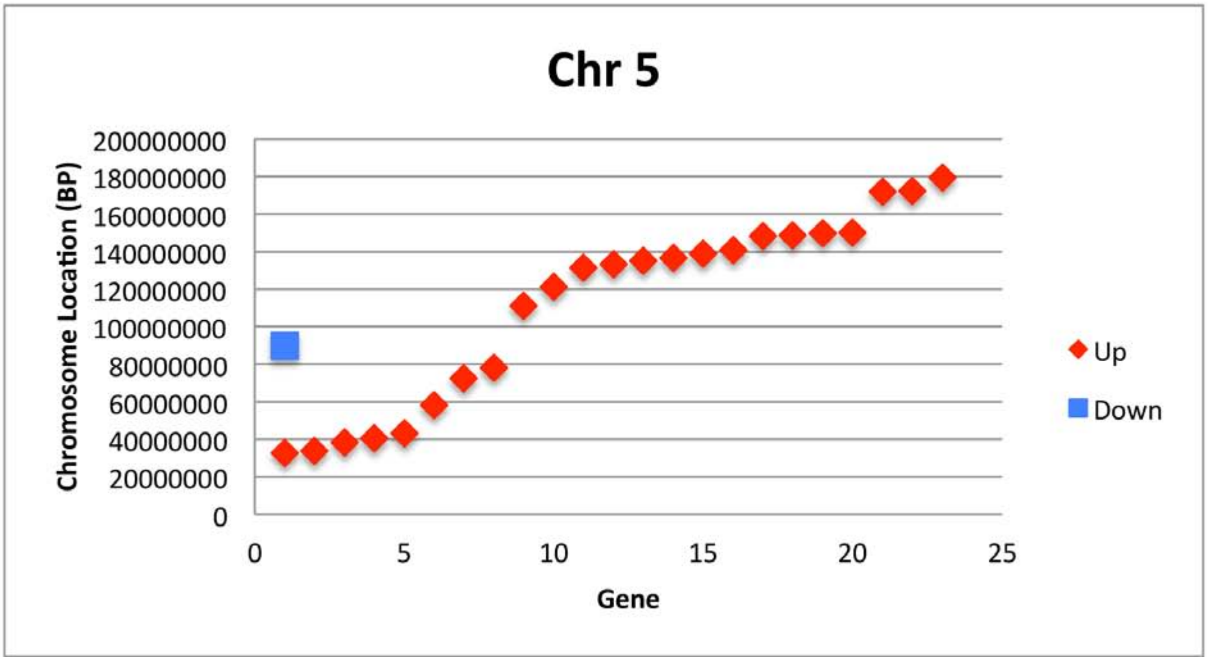

Gene Name	Gene Start (bp)	Gene End (bp)	Chromosome
TUBB2B	3224495	3231964	6
DSP	7541808	7586950	6
NEDD9	11183531	11382581	6
RNF182	13924677	13980533	6
MBOAT1	20101147	20101206	6
DCDC2	24171984	24358280	6
BTN3A2	26378257	26378316	6
HLA-F	29693280	29693338	6
HCG4	29759281	29759340	6
HLA-A	29909037	29913661	6
HLA-J	29977388	29977447	6
PSORS1C1	31107731	31107790	6
HLA-B	31321653	31321712	6
TNF	31545837	31545896	6
UNC5CL	40994772	41006928	6
VEGFA	43737921	43754224	6
ENPP4	46114082	46114141	6
ZNF292	87862551	87973914	6
PNRC1	89790470	89794879	6
RNF217	125404466	125404525	6
MOXD1	132617386	132617445	6
VNN2	133065009	133084598	6
TNFAIP3	138203679	138203738	6
CITED2	139693393	139695757	6
HIVEP2	143072604	143266338	6
AKAP12	151561134	151679692	6
SYTL3	159181680	159181739	6

Gene Name	Gene Start (bp)	Gene End (bp)	Chromosome
ZBTB9	33422356	33425325	6
SRSF3	36562145	36573377	6
FLJ13744	80017421	80017480	6
NDUFAF4	97337189	97345757	6
PLG	161123270	161174347	6

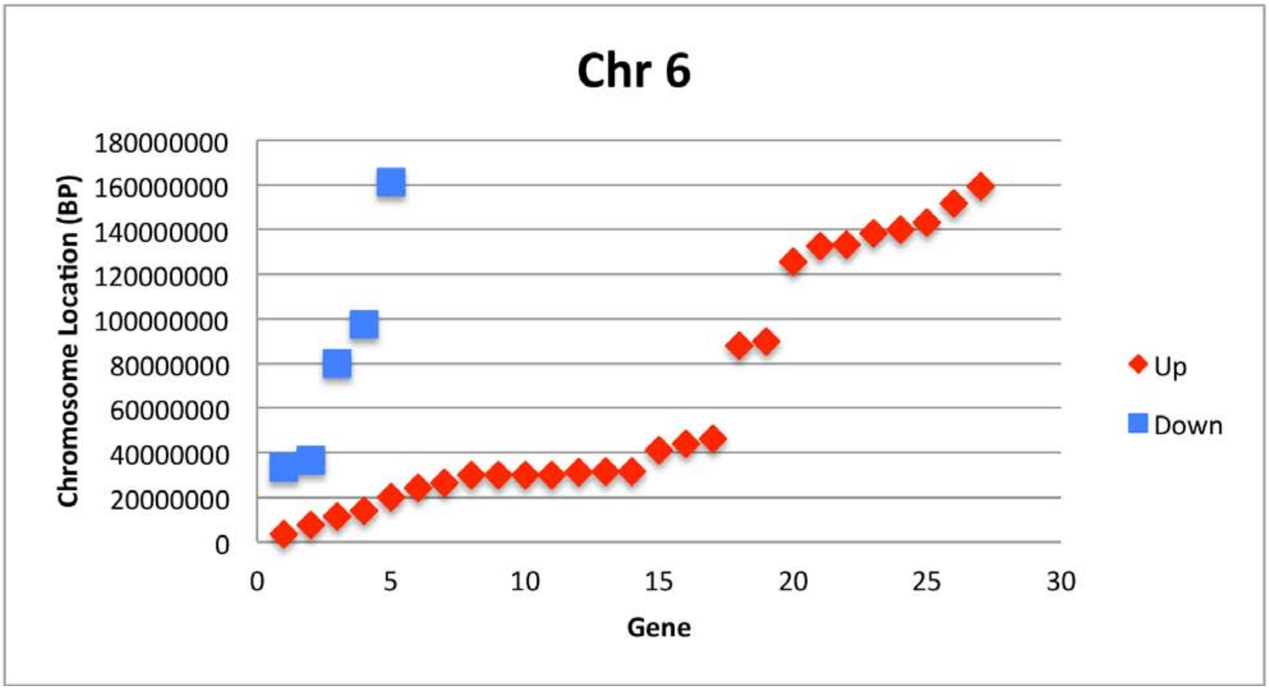

Gene Name	Gene Start (bp)	Gene End (bp)	Chromosome	Gene Name	Gene Start (bp)	Gene End (bp)	Chromosome
GPR146	1084212	1098897	7	ARL4A	12728454	12728514	7
MICALL2	1468101	1499138	7	TARP	38304978	38305037	7
JAZF1	27870377	27870436	7	CROT	86974997	87029111	7
VSTM2A	54610018	54638773	7	UFSP1	100486346	100487339	7
EGFR	55086714	55324313	7	ASZ1	117003276	117068177	7
ZNF713	55955169	56009918	7	ING3	120590803	120617270	7
VPS37D	73082155	73086442	7				
SRRM3	75831216	75916605	7				
SGCE	94214542	94285521	7				
BHLHA15	97840739	97842291	7				
PCOLCE	100204067	100204126	7				
EPO	100318423	100321323	7				
RASA4	102220258	102220317	7				
FAM115C	143318043	143427502	7				
WDR86	151078250	151078309	7				
HTR5A	154862034	154877459	7				
VIPR2	158820866	158937649	7				

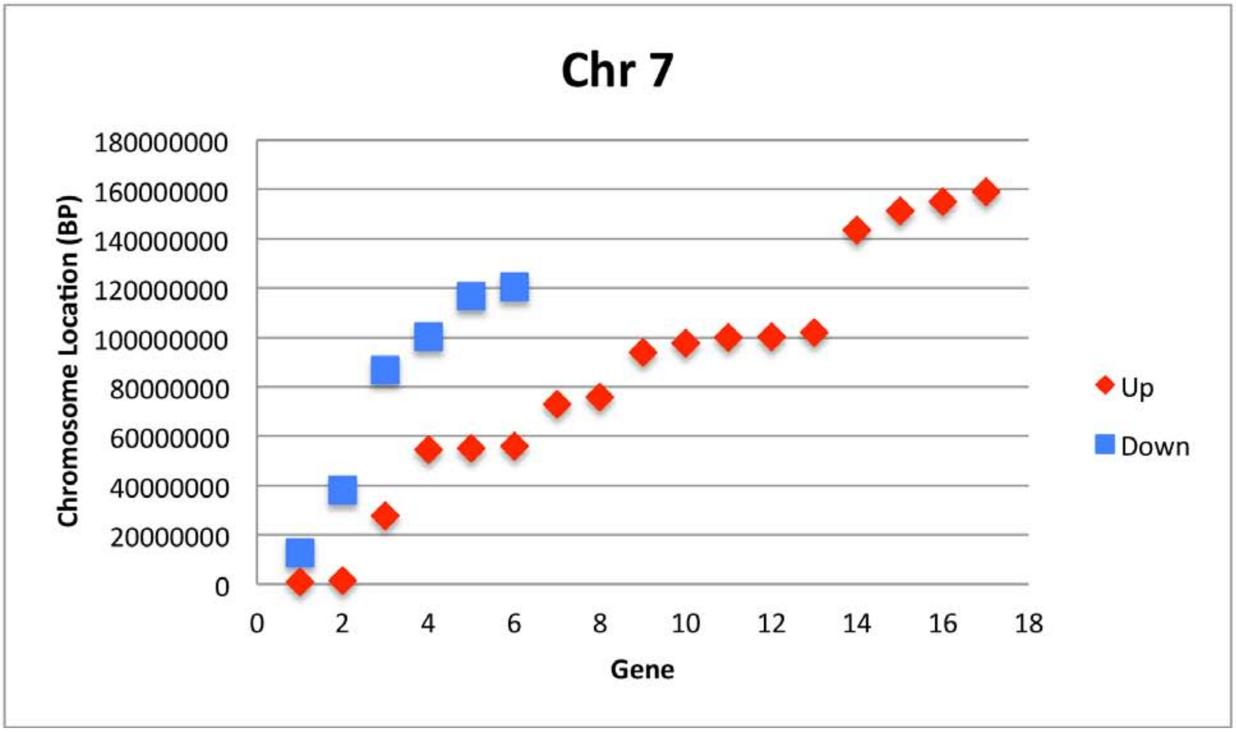

Gene Name	Gene Start (bp)	Gene End (bp)	Chromosome	Gene Name	Gene Start (bp)	Gene End (bp)	Chromosome
PPP1R3B	8993765	9009084	8	CSMD1	2792875	4852494	8
FAM167A	11278972	11332224	8	FAM86B1	12039605	12051642	8
TNFRSF10D	22993101	23021543	8	RRS1	67341263	67342966	8
STC1	23699428	23712320	8	CNGB3	87566205	87755903	8
BNIP3L	26240414	26363152	8	CCNE2	95891998	95908906	8
PNMA2	26362202	26371608	8	POP1	99129525	99172062	8
ZNF395	28203102	28260218	8	PKHD1L1	110543423	110543482	8
KIF13B	28924819	28924878	8	DSCC1	120847079	120847137	8
RNF122	33405273	33424643	8	PYCRL	144686083	144691943	8
PLEKHA2	38758753	38831428	8	FAM203A	145192672	145195746	8
PCMTD1	52730268	52730327	8				
CA8	61099906	61193971	8				
ASPH	62596599	62596658	8				
STAU2	74332864	74332923	8				
SNTB1	121547985	121825513	8				
FBXO32	124515388	124515447	8				
MTSS1	125563255	125563314	8				
TG	133879203	134147147	8				
NDRG1	134249414	134314265	8				
DENND3	142127377	142205907	8				
ARC	143692405	143696833	8				
LYNX1	143846092	143846151	8				

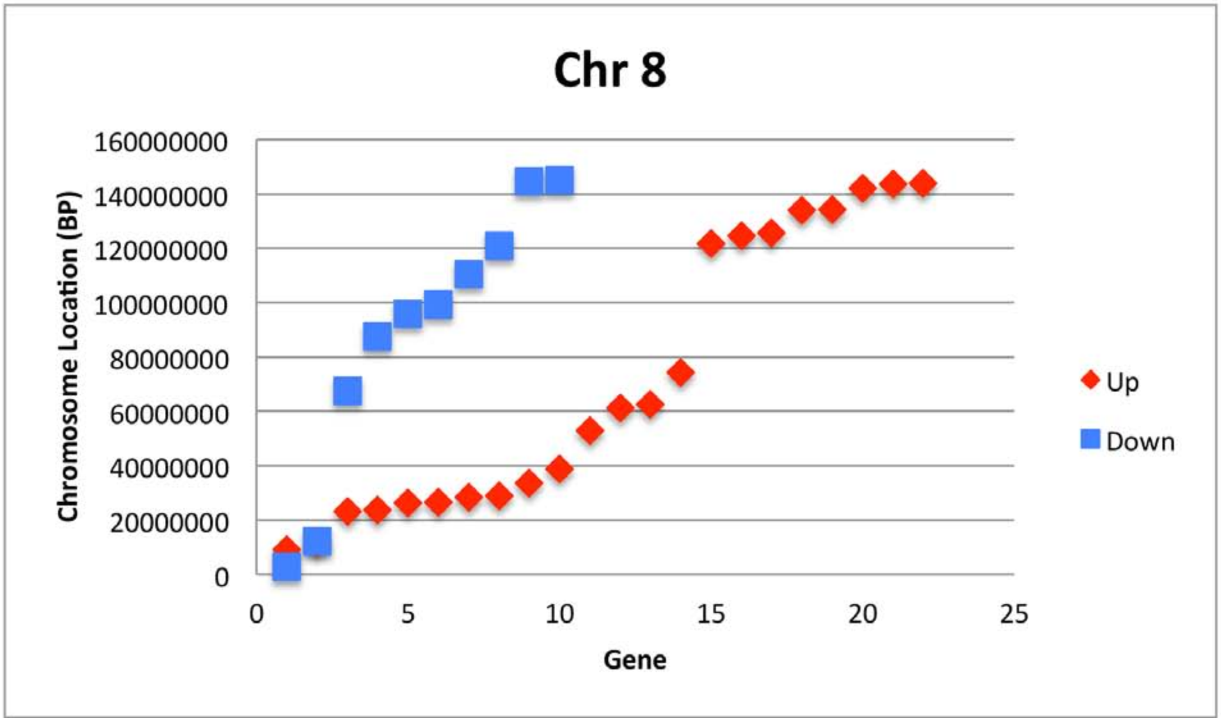

Gene Name	Gene Start (bp)	Gene End (bp)	Chromosome
KANK1	746006	746065	9
SLC1A1	4490444	4587469	9
MLLT3	20341663	20622542	9
CA9	35673853	35681156	9
FP588	35865315	35865374	9
FLJ41455	45447622	45447681	9
NTRK2	87283466	87638505	9
BARX1	96713905	96717654	9
EPB41L4B	111934255	112083244	9
KIAA1958	115249127	115431677	9
COL27A1	117073738	117073797	9
TNFSF15	117552239	117552298	9
GSN	123970072	124095121	9
MORN5	124922190	124962367	9
RALGPS1	129985021	129985080	9
SH2D3C	130500596	130541020	9
LCN15	139654086	139660707	9
KIAA1984	139701458	139701517	9
ENTPD8	140328816	140328875	9

Gene Name	Gene Start (bp)	Gene End (bp)	Chromosome
NOL6	33461439	33473928	9
ABCA1	107543283	107690518	9
SLC27A4	131102925	131123749	9
DOLK	131707809	131709898	9
SURF6	136197552	136203235	9

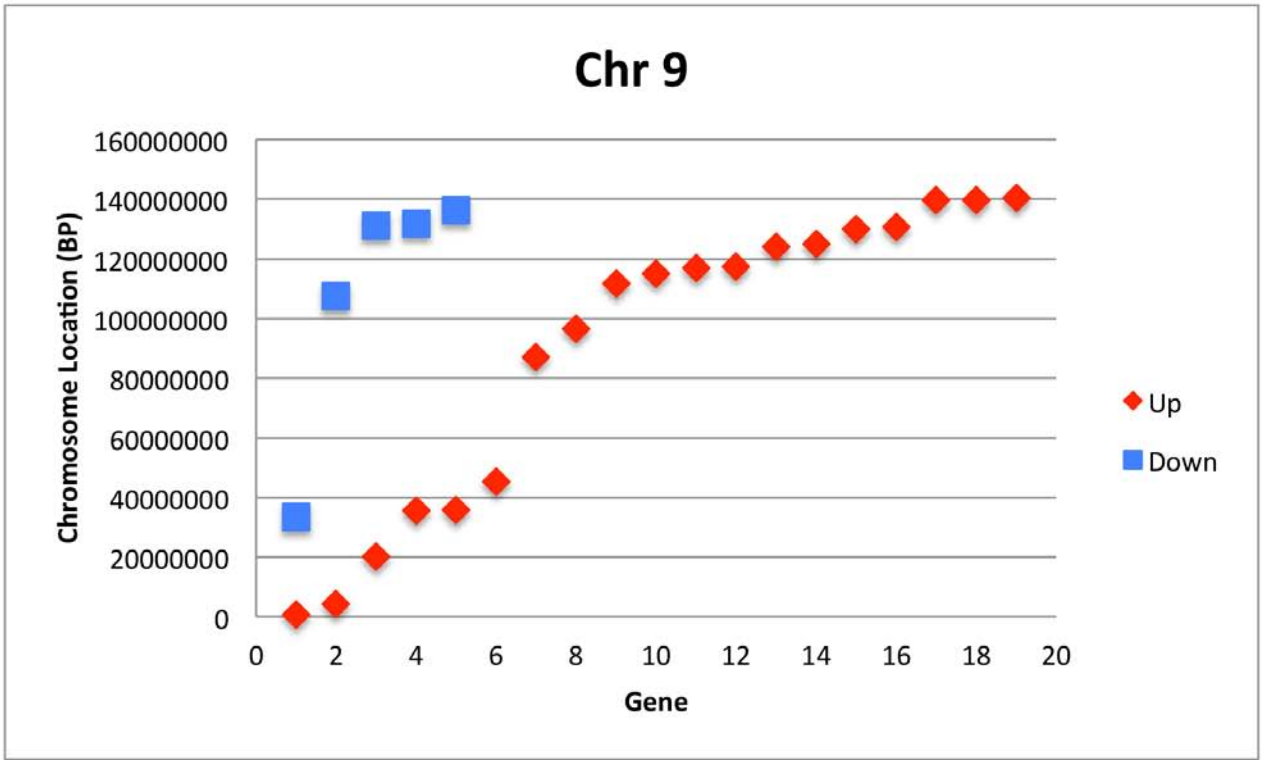

Gene Name	Gene Start (bp)	Gene End (bp)	Chromosome	Gene Name	Gene Start (bp)	Gene End (bp)	Chromosome
PFKP	3108525	3179904	10	CCDC147	106113522	106214848	10
AKR1C3	5147789	5147848	10	MXI1	111967363	112047123	10
PFKFB3	6186881	6277495	10	DUSP5	112257596	112271302	10
CAMK1D	12391481	12877545	10	CASC2	119805790	119969663	10
VIM	17270258	17279592	10	GPR26	125425871	125454123	10
MAP3K8	30749980	30750039	10	FANK1	127585108	127698161	10
PARD3	34398488	35104253	10	ADAM12	127700950	128077024	10
RASGEF1A	43689983	43762367	10	CLRN3	129676105	129691211	10
CXCL12	44793038	44881941	10	BNIP3	133781578	133795435	10
ALOX5	45869661	45941561	10	DPYSL4	134000404	134019280	10
CTNNA3	67680257	67680316	10	ZNF511	135121979	135166033	10
TET1	70332072	70332131	10	PDSS1	26986588	27035727	10
COL13A1	71561644	71724031	10	FZD8	35927177	35930362	10
P4HA1	74766975	74856732	10	MSMB	51549498	51562517	10
FLJ44715	75529183	75529242	10	ZNF365	64133951	64431771	10
FUT11	75532049	75540009	10	RPP30	92631473	92668312	10
NRG3	83635070	84746935	10	FGFBP3	93666346	93669240	10
GRID1	87359312	88126250	10	ADRB1	115803806	115806667	10
PAPSS2	89419370	89507462	10	PNLIPRP3	118187379	118237469	10
CYP26A1	94833232	94837647	10				
BLNK	97951458	98031344	10				
SEC31B	102246399	102289628	10				
SEMA4G	102729275	102745628	10				
SH3PXD2A	105348285	105615301	10				

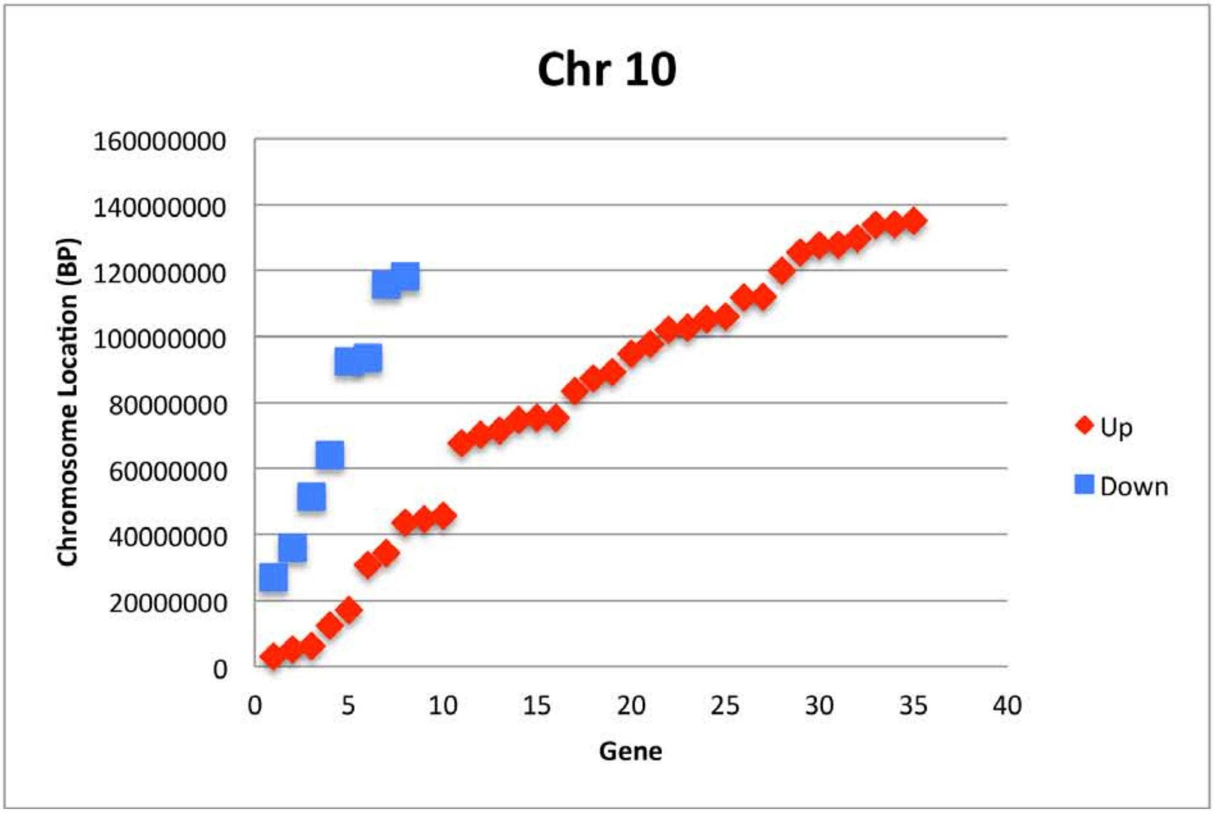

Gene Name	Gene Start (bp)	Gene End (bp)	Chromosome	Gene Name	Gene Start (bp)	Gene End (bp)	Chromosome
MUC6	1016826	1016885	11	NUP98	3692313	3819022	11
BRSK2	1411129	1483919	11	WT1	32409321	32457176	11
IFITM10	1753640	1771821	11	F2	46760913	46760973	11
ASCL2	2289725	2292182	11	FEN1	61564029	61564087	11
CDKN1C	2904443	2907111	11	LRRN4CL	62453874	62457371	11
CYB5R2	7686331	7698453	11	POLA2	65029233	65073060	11
AMPD3	10329860	10529126	11	NDUFC2	77779350	77791265	11
MYOD1	17741115	17743678	11	ANKRD42	82904781	82971736	11
LDHC	18433854	18473605	11	CHORDC1	89933582	89933613	11
PHF21A	45950945	45951004	11	ALKBH8	107373452	107436472	11
LRP4	46878549	46878608	11	NCRNA0016'	129874964	129875023	11
GLYAT	58407899	58499447	11				
VEGFB	64002010	64006259	11				
NRXN2	64373646	64490660	11				
RASGRP2	64508420	64508476	11				
PPP2R5B	64685025	64701945	11				
EHBP1L1	65357976	65358035	11				
EFEMP2	65633912	65641063	11				
MTL5	68474908	68519032	11				
BIRC3	102188215	102210134	11				
GUCY1A2	106544738	106889250	11				
NCAM1	112831997	113149158	11				
IL10RA	117857063	117872196	11				
SCN4B	118004092	118023603	11				
TMEM45B	129685714	129729898	11				

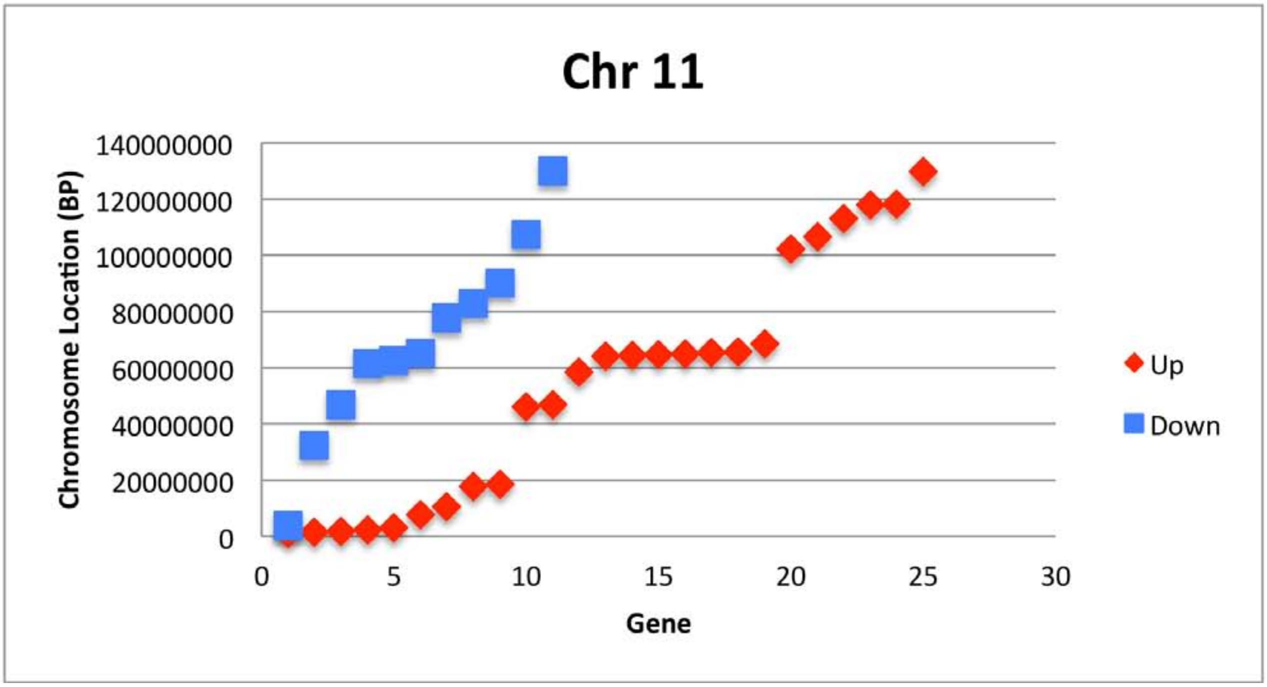

Gene Name	Gene Start (bp)	Gene End (bp)	Chromosome	Gene Name	Gene Start (bp)	Gene End (bp)	Chromosome
CCND2	4382938	4414516	12	NOP2	6666029	6677857	12
NTF3	5604039	5604098	12	FAM86FP	8383645	8383704	12
ENO2	7022909	7032861	12	CLEC2A	10051272	10084980	12
RBP5	7280873	7280932	12	CLEC7A	10269366	10269408	12
CDKN1B	12867992	12875305	12	MAGOHB	10757309	10757368	12
APOLD1	12944179	12944238	12	SLC2A13	40148823	40499891	12
KIAA1467	13197218	13295455	12	SCAF11	46312914	46385903	12
PRPH	49687035	49692465	12	ARHGAP9	57866038	57882597	12
C1QL4	49726260	49726319	12	TBC1D30	65174589	65274812	12
IGFBP6	53491220	53496129	12	PTPRQ	81073739	81073798	12
LRP1	57522276	57607134	12	SPIC	101869199	101880775	12
METTL21B	58175640	58175699	12	PGAM5	133287405	133299228	12
TCP11L2	106695707	106740793	12				
SIRT4	120750879	120750938	12				
WDR66	122355768	122441833	12				
B3GNT4	122688090	122693499	12				

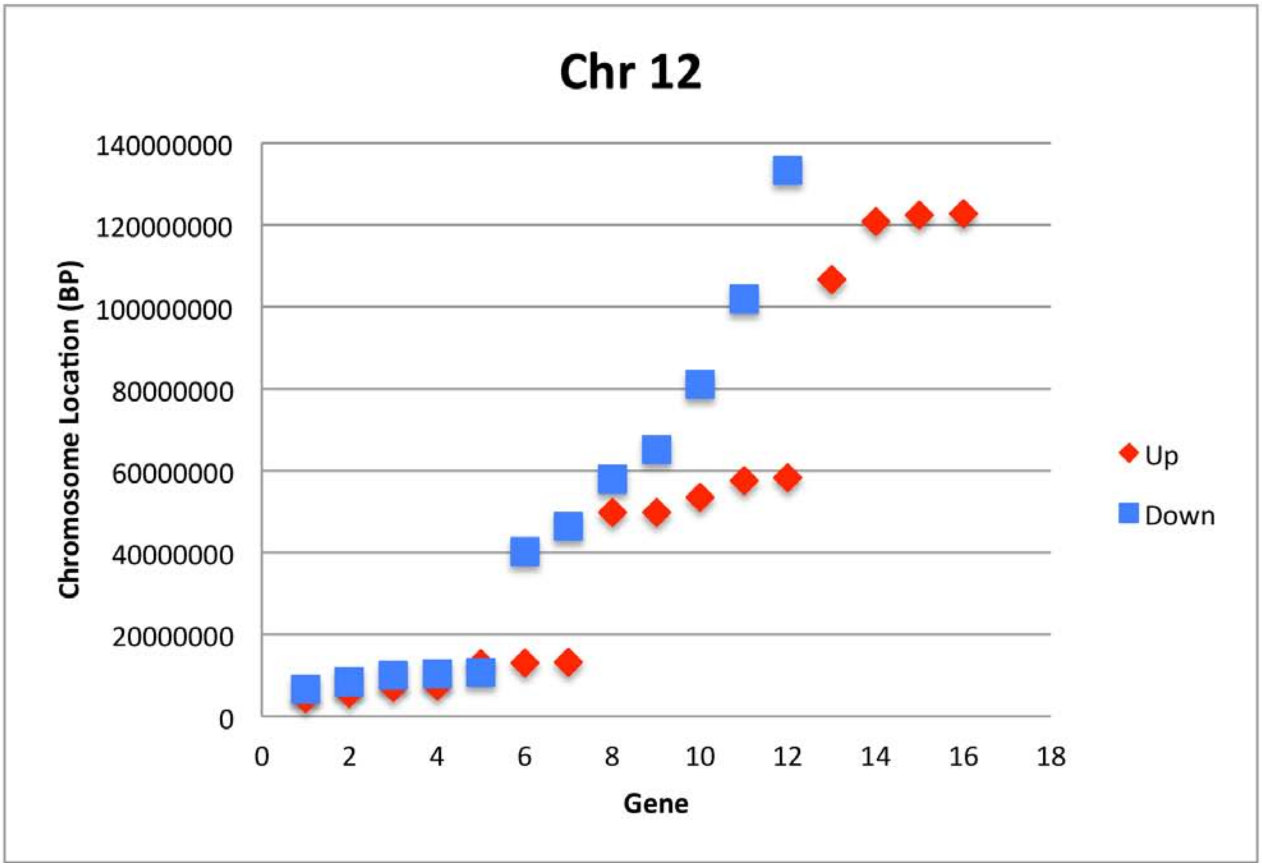

Gene Name	Gene Start (bp)	Gene End (bp)	Chromosome	Gene Name	Gene Start (bp)	Gene End (bp)	Chromosome
ATP8A2	25946209	26599989	13	PSPC1	20248896	20357142	13
FRY	32605437	32870794	13	HSPH1	31710762	31736525	13
TRIM13	50587010	50587069	13	RFC3	34392186	34540695	13
KCNRG	50594618	50594677	13	KLHL1	70274726	70682591	13
MZT1	73282634	73282693	13				

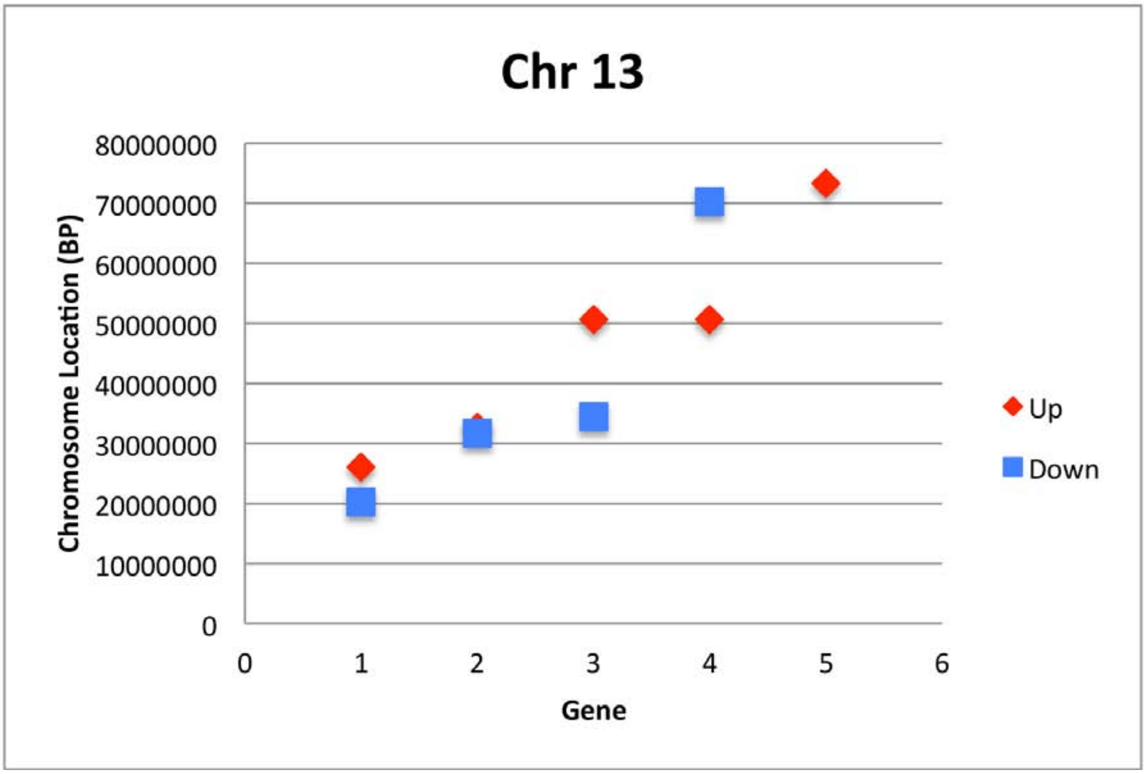

Gene Name	Gene Start (bp)	Gene End (bp)	Chromosome	Gene Name	Gene Start (bp)	Gene End (bp)	Chromosome
RNASE4	21152259	21168761	14	PNN	39644387	39652422	14
NFATC4	24834879	24848810	14	DPF3	73086004	73360809	14
TTC6	38065052	38510647	14	RPS6KL1	75370657	75390099	14
SAV1	51098776	51135049	14				
PYGL	51324609	51411454	14				
ERO1L	53106634	53162618	14				
SPTB	65213002	65346601	14				
FOS	75745477	75748933	14				
COX8C	93813537	93814702	14				
ASB2	94400499	94443137	14				
PPP4R4	94745813	94745872	14				
AK7	96953277	96953336	14				

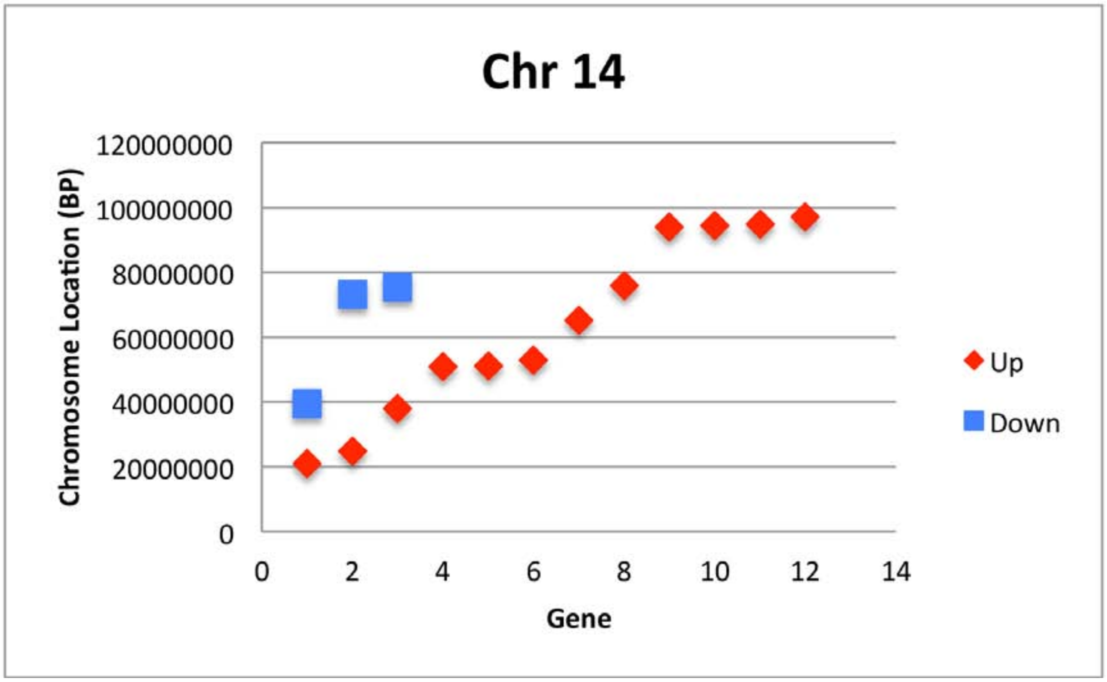

Gene Name	Gene Start (bp)	Gene End (bp)	Chromosome	Gene Name	Gene Start (bp)	Gene End (bp)	Chromosome
PHGR1	40643234	40648635	15	CHAC1	41245160	41248710	15
ITPKA	41785591	41795747	15	SNAPC5	66782473	66790151	15
PLA2G4D	42359207	42386752	15	CLN6	68499330	68549549	15
SHF	45460006	45460065	15				
GLDN	51633826	51700210	15				
MYO1E	59427113	59665099	15				
RORA	60780483	61521518	15				
PAQR5	69591286	69700119	15				
CLK3	74922387	74922446	15				
SCAPER	76640850	76640909	15				
CIB2	78396948	78423886	15				
SH3GL3	84115980	84287495	15				

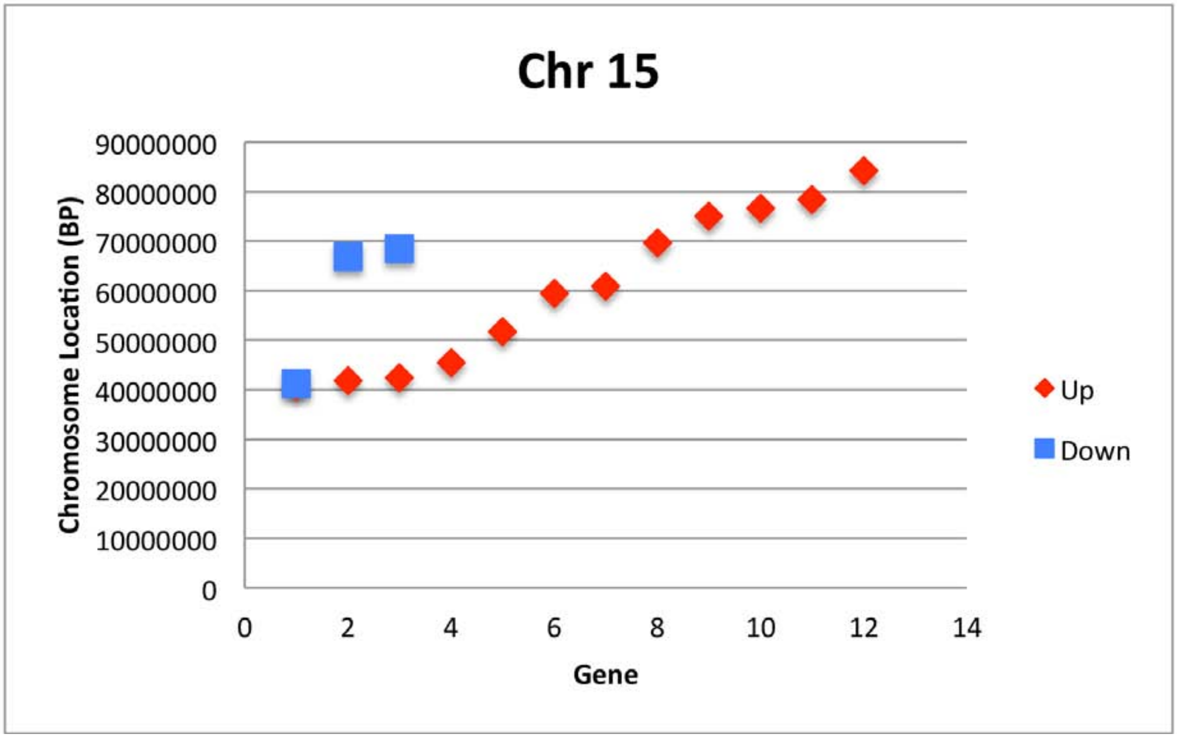

Gene Name	Gene Start (bp)	Gene End (bp)	Chromosome	Gene Name	Gene Start (bp)	Gene End (bp)	Chromosome
CACNA1H	1203241	1271771	16	ZNF597	3486104	3493542	16
RAB26	2204081	2204140	16	ALG1	5083703	5137380	16
PRSS22	2902781	2902840	16	DCTPP1	30434940	30441396	16
GLIS2	4364762	4389598	16	FUS	31191431	31203127	16
VASN	4421849	4433529	16	HAS3	69139467	69152622	16
SEC14L5	5008318	5069159	16	ADAT1	75630879	75657198	16
CARHSP1	8946799	8962866	16	FLJ30679	86589793	86589852	16
ABCC6P1	18609500	18609559	16				
SYT17	19179293	19279652	16				
TMEM159	21191196	21191255	16				
CRYM	21250195	21314404	16				
SCNN1G	23227547	23227605	16				
SCNN1B	23289552	23392620	16				
MVP	29831715	29859355	16				
YPEL3	30104047	30104106	16				
ABCC11	48201053	48201112	16				
CHST6	75507022	75507046	16				
SNAI3	88744422	88744481	16				

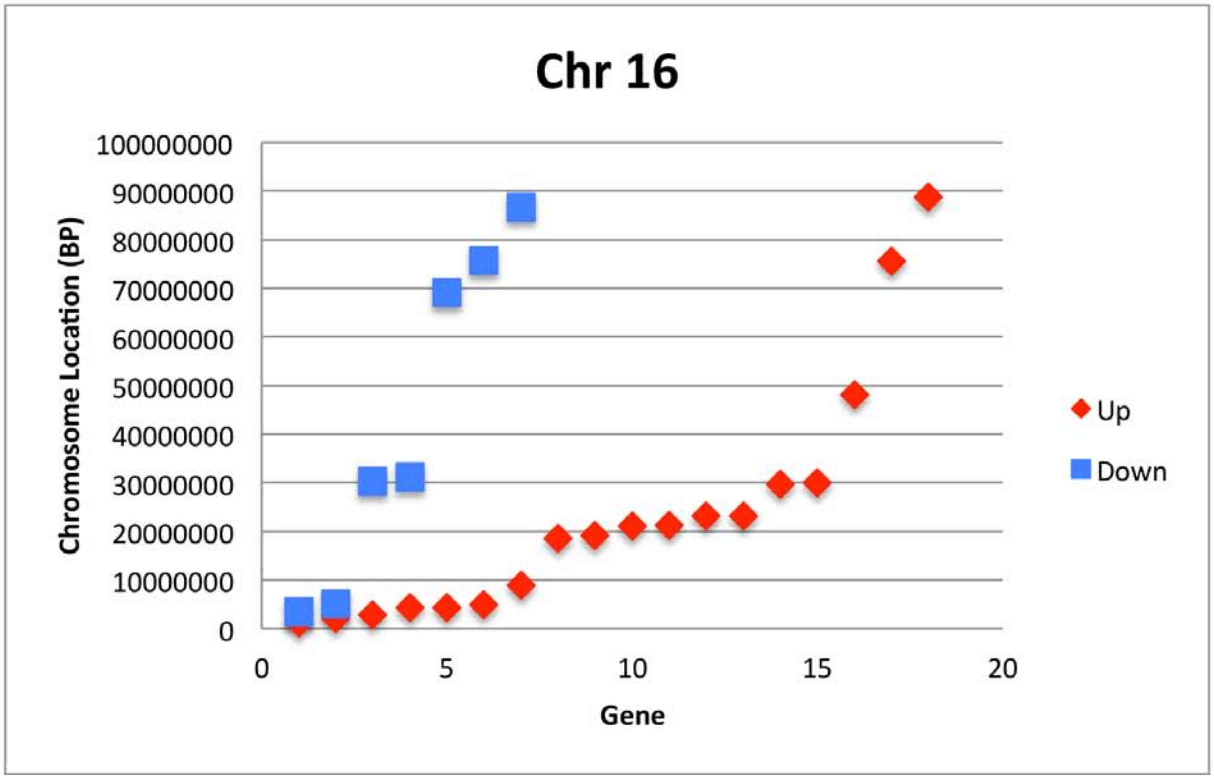

Gene Name	Gene Start (bp)	Gene End (bp)	Chromosome	Gene Name	Gene Start (bp)	Gene End (bp)	Chromosome
PITPNM3	6354585	6354644	17	SLC43A2	1472561	1532180	17
ASGR1	7076750	7082883	17	PIGW	34890847	34895159	17
HES7	8023908	8027410	17	IKZF3	37921198	38020441	17
NCRNA00324	8124429	8124488	17	SRSF2	74730197	74733456	17
NTN1	9147220	9147279	17	CHMP6	78965398	78983317	17
RASD1	17397751	17399709	17	PCYT2	79858841	79869340	17
SMCR6	17729617	17729676	17				
MYO15A	18012020	18083116	17				
RNF112	19314438	19320589	17				
WSB1	25621102	25640657	17				
ALDOC	26900133	26904282	17				
GSDMB	38060850	38060909	17				
ACLY	40023161	40086795	17				
ARHGAP27	43506719	43506778	17				
CRHR1	43699267	43913194	17				
MAPT	43971748	44105700	17				
OSBPL7	45884738	45899200	17				
LUC7L3	48796905	48833574	17				
YPEL2	57409050	57479090	17				
EFCAB3	60493612	60493671	17				
MRC2	60704762	60770958	17				
TCAM1P	61926652	61941739	17				
ABCA5	67243530	67243589	17				
FLJ37644	70067195	70067254	17				
FLJ26484	70594182	70594241	17				
DNAI2	72270386	72311023	17				
CD300A	72462555	72480933	17				
ITGB4	73753824	73753883	17				
NOTUM	79910388	79919716	17				
SLC16A3	80186273	80219005	17				

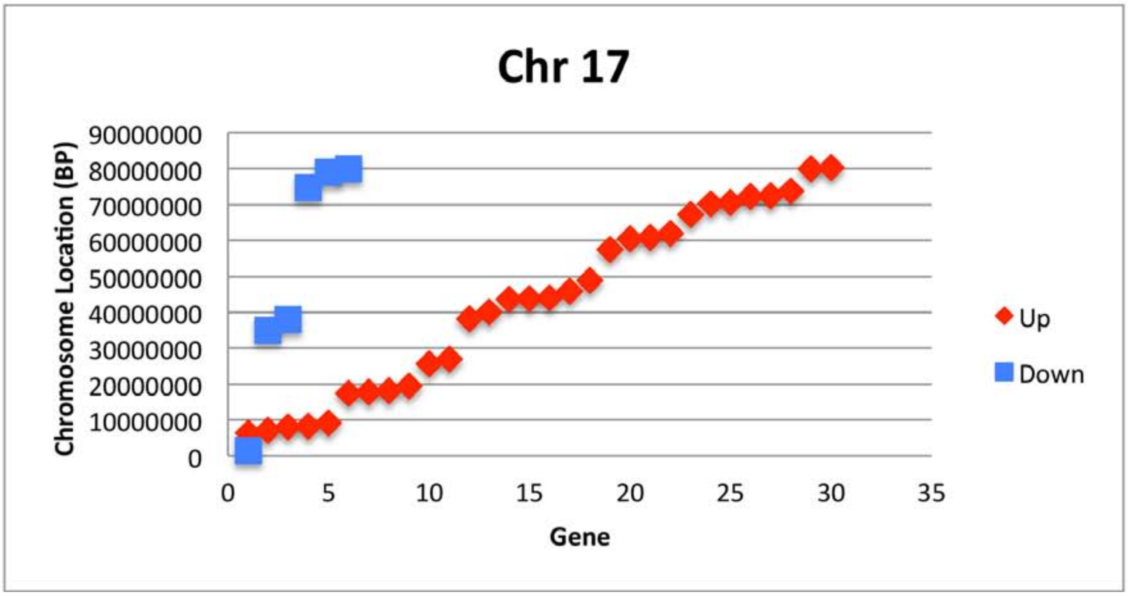

Gene Name	Gene Start (bp)	Gene End (bp)	Chromosome	Gene Name	Gene Start (bp)	Gene End (bp)	Chromosome
MYOM1	3067166	3067225	18	DSC1	28709199	28742819	18
ANKRD12	9136226	9285983	18	ELAC1	48494361	48514491	18
RNF165	43906772	44043103	18	CNDP1	72201675	72254448	18
PIAS2	44388353	44500123	18				

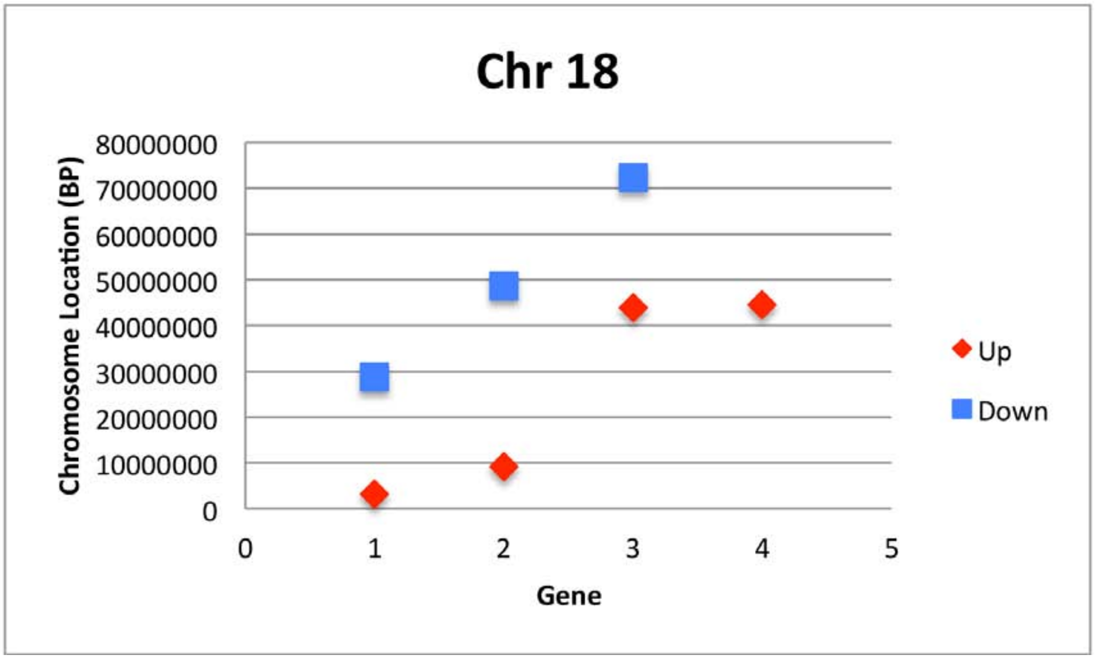

Gene Name	Gene Start (bp)	Gene End (bp)	Chromosome	Gene Name	Gene Start (bp)	Gene End (bp)	Chromosome
PALM	748269	748328	19	ATP4A	36040945	36054560	19
PRTN3	840963	848175	19	CLIP3	36505562	36524245	19
KISS1R	917287	921015	19	KCNK6	38818817	38818876	19
ARID3A	925781	975939	19	TGFB1	41807492	41859816	19
GRIN3B	1000418	1009731	19	ZNF404	44376515	44405537	19
PLK5	1524073	1535455	19	BCL3	45250962	45263301	19
ATP8B3	1782074	1812275	19	APOE	45409011	45412650	19
MKNK2	2037470	2051243	19	RELB	45541385	45541444	19
DOT1L	2232170	2232229	19	PPP1R13L	45882892	45909607	19
GADD45B	2476120	2478257	19	BBC3	47724081	47736023	19
TLE6	2977444	2995177	19	CCDC114	48799714	48825151	19
S1PR4	3172344	3180329	19	EMP3	48832675	48832734	19
CD70	6585867	6585926	19	RRAS	50138733	50138792	19
INSR	7112351	7112410	19	MYBPC2	50936160	50969578	19
PCP2	7696497	7698634	19	TNNT1	55644162	55660722	19
ANGPTL4	8428173	8439257	19	BRSK1	55823620	55823679	19
ZSWIM4	13942978	13943037	19	IL11	55875788	55875847	19
NOTCH3	15270444	15311792	19	FLJ45684	644371	644430	19
JAK3	17935589	17958880	19	MUM1	1285890	1378430	19
RAB3A	18307612	18307671	19	TIMM13	2425622	2427892	19
JUND	18390563	18392432	19	MPV17L2	18303992	18307758	19
PGPEP1	18480282	18480341	19	YIF1B	38794254	38794313	19
GDF15	18499890	18499949	19	ZNF552	58315209	58326281	19
WTIP	34971874	34997258	19				
HPN	35556490	35556549	19				
DMKN	35988135	35988194	19				

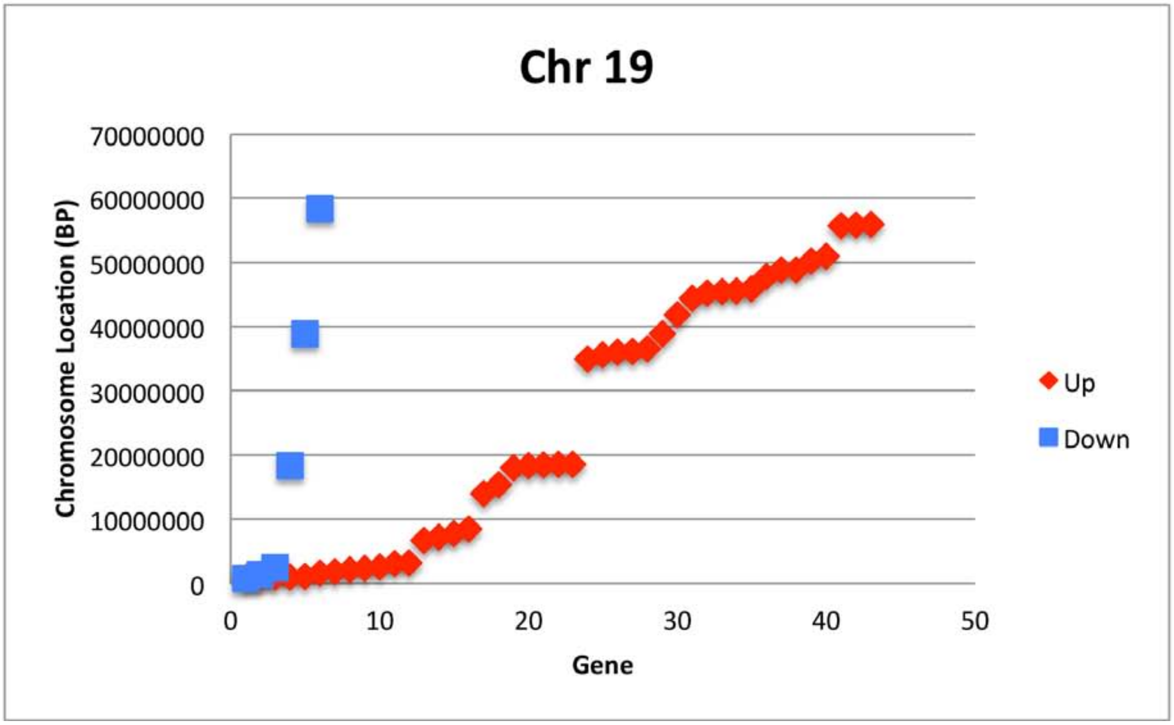

Gene Name	Gene Start (bp)	Gene End (bp)	Chromosome	Gene Name	Gene Start (bp)	Gene End (bp)	Chromosome
DDRGK1	3170996	3185331	20	NOP56	2632791	2639039	20
RNF24	3907956	3996229	20	GNRH2	3024268	3026393	20
MACROD2	14066315	14066374	20	FITM2	42931478	42939809	20
MMP24	33814457	33864801	20	FLJ40606	44562739	44562798	20
LPIN3	39969560	39989222	20				
WISP2	43343485	43357150	20				
PLTP	44527399	44540794	20				
PREX1	47240790	47444420	20				
GATA5	61038553	61051026	20				

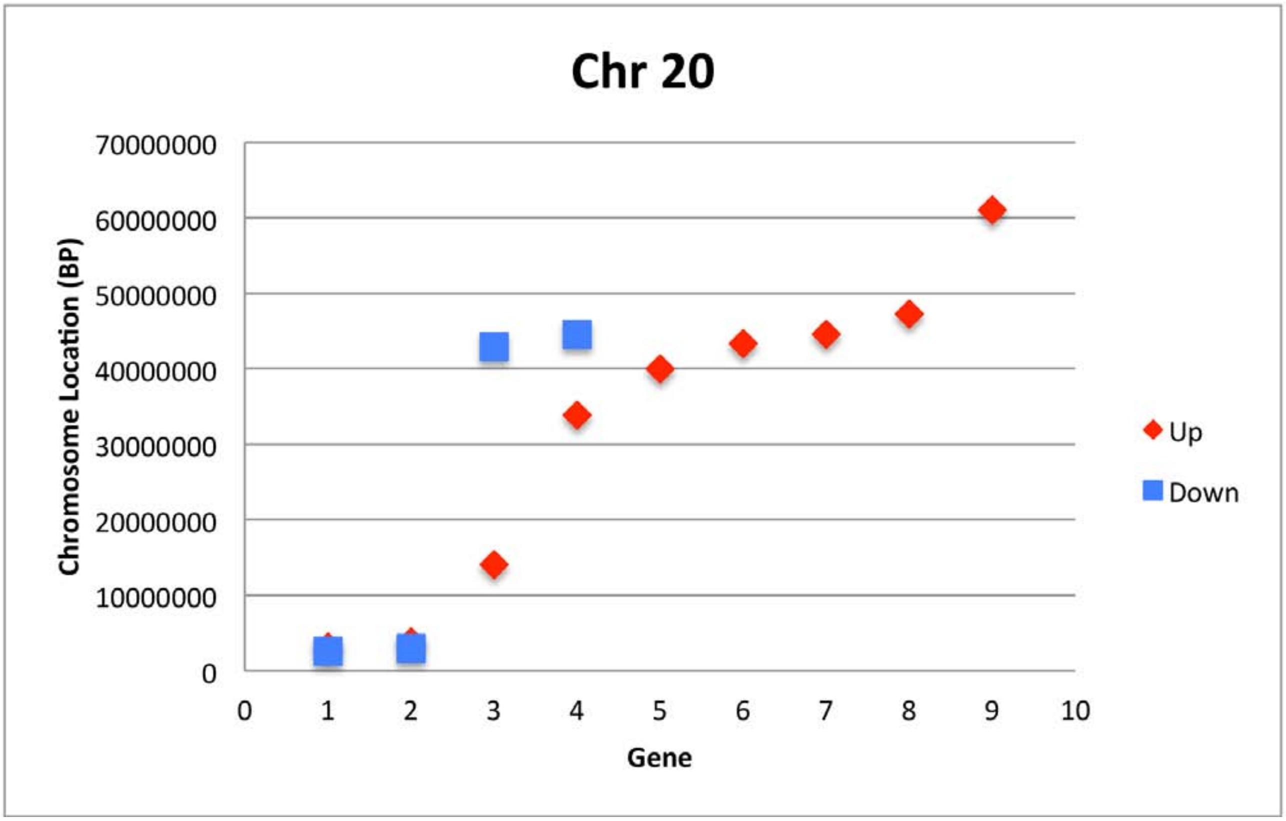

Gene Name	Gene Start (bp)	Gene End (bp)	Chromosome	Gene Name	Gene Start (bp)	Gene End (bp)	Chromosome
NCAM2	22915446	22915505	21	CLDN8	31586324	31588391	21
TMPRSS2	42836478	42903043	21	TRPM2	45770046	45862964	21
RIPK4	43159529	43187266	21				
TFF3	43731777	43735761	21				
TSPEAR	45917775	46131495	21				
KRTAP10-12	46117087	46117959	21				
ITGB2	46305868	46351904	21				

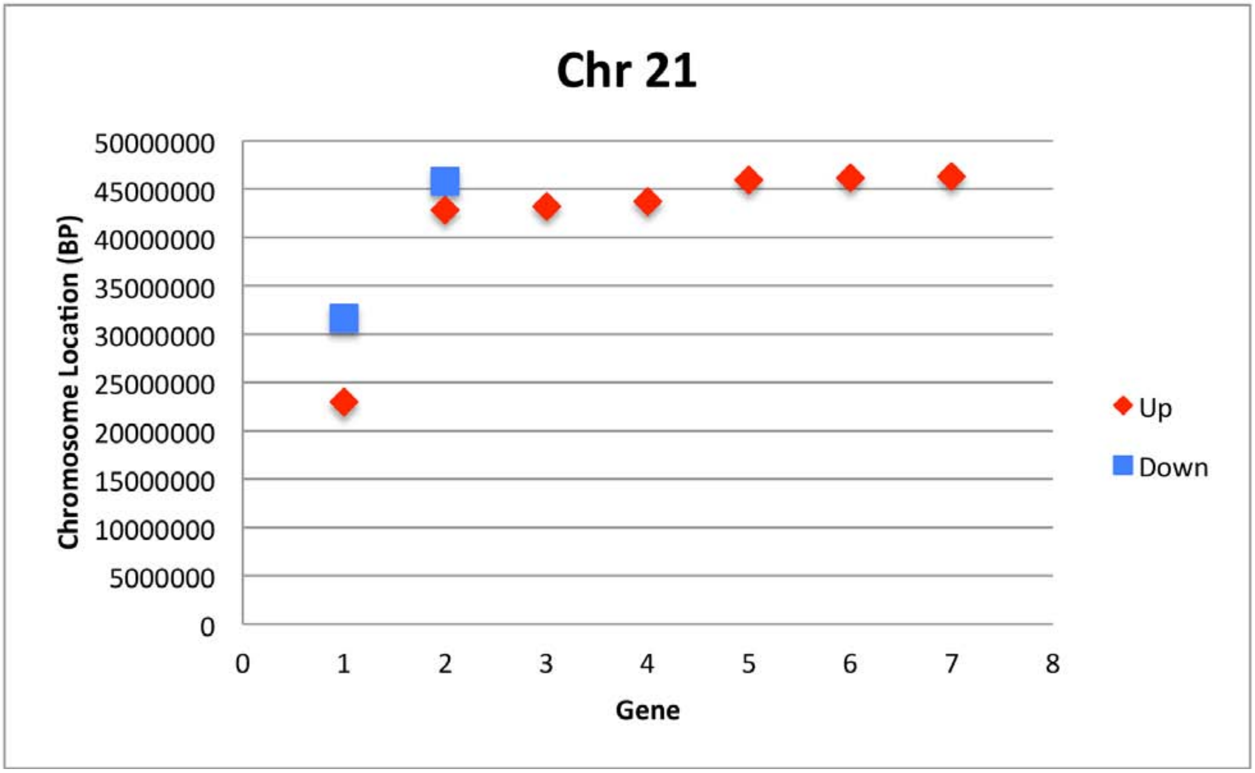

Gene Name	Gene Start (bp)	Gene End (bp)	Chromosome
CECR5-AS1	17640274	17646335	22
MICAL3	18270415	18507325	22
YPEL1	22051833	22090123	22
GAL3ST1	30950622	30970574	22
HMOX1	35776354	35790207	22
ELFN2	37764187	37764246	22
MAFF	38597889	38612518	22
SYNGR1	39780995	39781054	22
PHF21B	45277042	45405880	22

Gene Name	Gene Start (bp)	Gene End (bp)	Chromosome
UQCR10	30163358	30166402	22

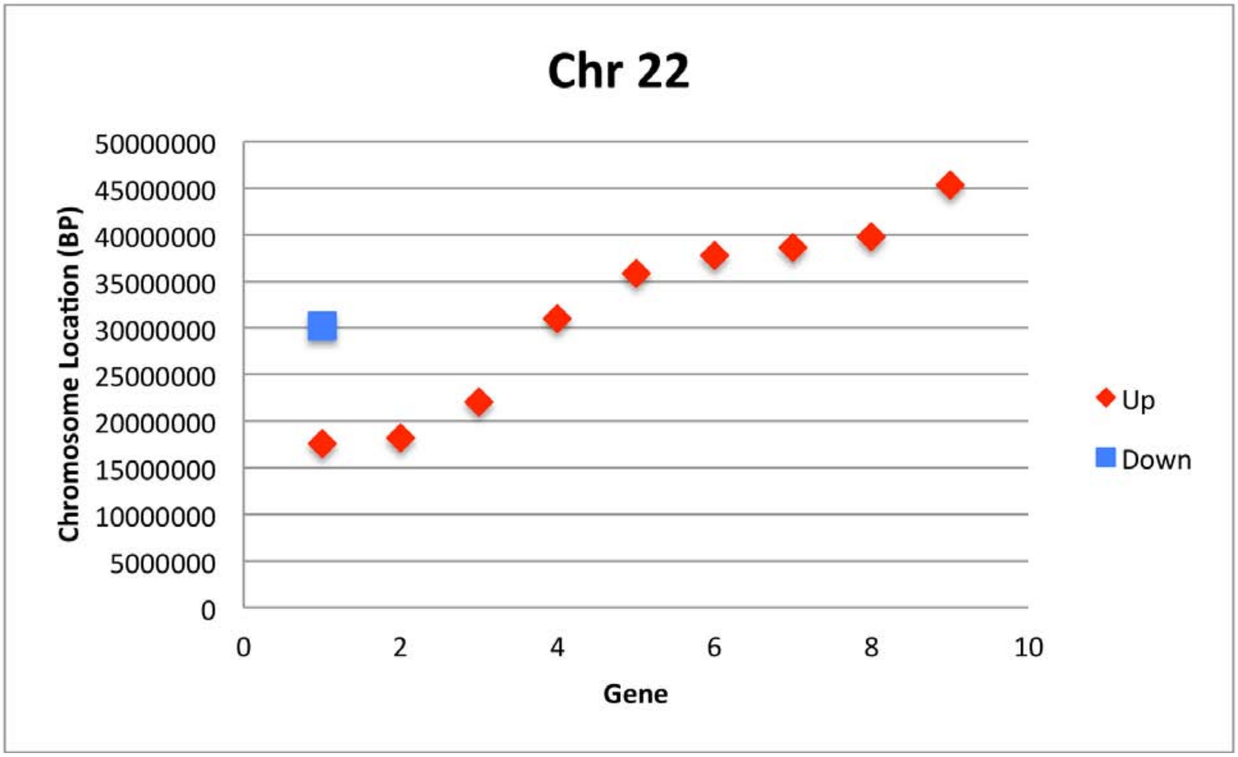

Gene Name	Gene Start (bp)	Gene End (bp)	Chromosome
MAP3K15	19378174	19533379	X
IL1RAPL1	29973954	29974013	X
SRPX	38008713	38008772	X
PIM2	48770771	48770830	X
PAGE2B	55101496	55105342	X
FOXO4	70322689	70322748	X
KIAA2022	73952684	74145282	X
PGK1	77320685	77384793	X
BRWD3	79926353	80065187	X
ARMCX4	100673275	100788446	X
TBC1D8B	106045910	106119375	X
TSC22D3	106956451	107020572	X
CHRDL1	109917220	109917279	X
RHOXF1	119243011	119249847	X
BCORL1	129191457	129191516	X
RAB33A	129305623	129318844	X
HS6ST2	131760088	131760147	X

Gene Name	Gene Start (bp)	Gene End (bp)	Chromosome
TAF9B	77385245	77395203	X
NAP1L3	92925929	92928567	X
L1CAM	153126969	153174677	X

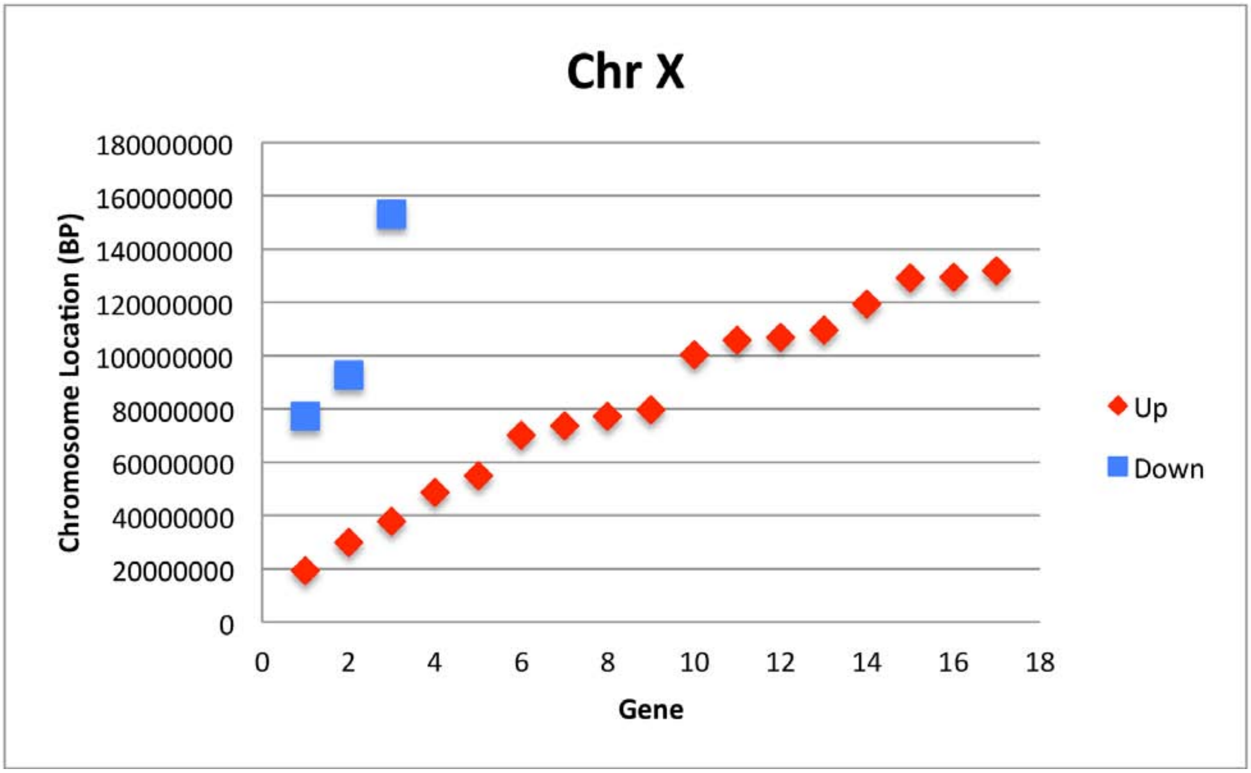

Supplementary Data File 3

Analysis Name: 50UR Rb_h vs Sc_n - 2014-01-30 04:59 PM
Analysis Creation Date: 2014-01-30
Build version: 261899
Content version: 18030641 (Release Date: 2013-12-06)

Analysis settings

View
Reference set: Ingenuity Knowledge Base (Genes Only)
Relationship to include: Direct and Indirect
Includes Endogenous Chemicals
Optional Analyses: My Pathways My List

Filter Summary:
Consider only relationships where
confidence = Experimentally Observed
Cutoff:

Top Networks

ID	Associated Network Functions	Score
1	Cardiac Arteriopathy, Cardiovascular Disease, Hematological Disease	44
2	Organ Morphology, Organismal Development, Reproductive System Development and Function	39
3	Cellular Development, Cellular Growth and Proliferation, Hematological System Development and Function	20
4	Organ Morphology, Reproductive System Development and Function, Endocrine System Development and Function	3

(c) 2000-2014 Ingenuity Systems, Inc. All rights reserved.

(c) 2000-2014 Ingenuity Systems, Inc. All rights reserved.

Top Diseases and Bio Functions

Diseases and Disorders

Name	p-value	# Molecules
Cancer	1.20E-05 - 2.16E-02	32
Neurological Disease	1.41E-04 - 1.92E-02	17
Cardiovascular Disease	1.28E-03 - 1.92E-02	10
Hematological Disease	1.28E-03 - 1.20E-02	10
Metabolic Disease	1.28E-03 - 1.20E-02	7

Molecular and Cellular Functions

Name	p-value	# Molecules
Cell Death and Survival	1.33E-04 - 1.92E-02	10
Cellular Movement	1.42E-04 - 2.13E-02	17
Cellular Development	2.41E-04 - 2.16E-02	17
Cell-To-Cell Signaling and Interaction	3.11E-04 - 2.13E-02	12
Cellular Compromise	3.35E-04 - 1.92E-02	7

Physiological System Development and Function

Name	p-value	# Molecules
Organismal Development	2.68E-05 - 2.16E-02	20
Digestive System Development and Function	3.69E-05 - 2.00E-02	9
Organ Morphology	7.14E-05 - 2.00E-02	15
Reproductive System Development and Function	7.14E-05 - 1.92E-02	11
Tumor Morphology	1.33E-04 - 1.97E-02	6

(c) 2000-2014 Ingenuity Systems, Inc. All rights reserved.

Top Canonical Pathways

Name	p-value	Ratio
Gluconeogenesis I	1.4E-03	2/47 (0.043)
Glycolysis I	1.53E-03	2/41 (0.049)
PI3K Signaling in B Lymphocytes	3.66E-03	3/143 (0.021)
April Mediated Signaling	3.81E-03	2/44 (0.045)
B Cell Activating Factor Signaling	4.21E-03	2/46 (0.043)

Top Molecules

Other up-regulated

Molecules	Exp. Value	Exp. Chart
HTR5A	↑256.399	
PLOD2	↑219.023	
SLC16A3	↑200.172	
ATP4A	↑157.926	
PLA2G4D	↑97.723	
NIM1	↑91.574	
CYP26A1	↑68.505	
CXCR4	↑62.557	
KISS1R	↑61.942	
ANGPTL4	↑53.166	

Other down-regulated

(c) 2000-2014 Ingenuity Systems, Inc. All rights reserved.

INGENUITY SYSTEMS

Molecules	Exp. Value	Exp. Chart

Top Upstream Regulators

Upstream Regulator	p-value of overlap	Predicted Activation State
HIF1A	2.49E-15	Activated
EPAS1	2.30E-14	Activated
farnesylthiosalicylic acid	1.83E-11	Inhibited
NEDD9	1.41E-08	Activated
VHL	6.75E-06	

(c) 2000-2014 Ingenuity Systems, Inc. All rights reserved.

INGENUITY® SYSTEMS

Top My Lists

Name	p-value	Ratio

Top My Pathways

Name	p-value	Ratio

Top Tox Lists

Name	p-value	Ratio
Increases Cardiac Dysfunction	3.61E-03	2/37 (0.054)
Cytochrome P450 Panel - Substrate is a Vitamin (Human)	1.44E-02	1/6 (0.167)
Cytochrome P450 Panel - Substrate is a Vitamin (Mouse)	1.68E-02	1/7 (0.143)
Cytochrome P450 Panel - Substrate is a Vitamin (Rat)	1.68E-02	1/7 (0.143)
Primary Glomerulonephritis Biomarker Panel (Human)	2.63E-02	1/11 (0.091)

(c) 2000-2014 Ingenuity Systems, Inc. All rights reserved.

INGENUITY® SYSTEMS

Top Tox Functions

Assays: Clinical Chemistry and Hematology

Name	p-value	# Molecules
Increased Levels of Potassium	3.57E-02 - 3.57E-02	1

Cardiotoxicity

Name	p-value	# Molecules
Cardiac Dysfunction	1.28E-03 - 1.28E-03	2
Cardiac Arteriopathy	4.83E-03 - 3.55E-02	3
Cardiac Fibrosis	1.68E-02 - 1.68E-02	1
Heart Failure	2.56E-02 - 2.23E-01	3
Cardiac Hypertrophy	3.84E-02 - 4.84E-01	2

Hepatotoxicity

Name	p-value	# Molecules
Liver Hematopoiesis	1.44E-02 - 1.44E-02	1
Liver Hyperplasia/Hyperproliferation	2.65E-02 - 3.19E-01	5
Liver Damage	3.34E-02 - 3.34E-02	1
Liver Proliferation	3.34E-02 - 5.41E-02	2
Liver Hypoplasia	9.91E-02 - 9.91E-02	1

Nephrotoxicity

Name	p-value	# Molecules
Renal Proliferation	1.20E-02 - 6.43E-02	2

(c) 2000-2014 Ingenuity Systems, Inc. All rights reserved.

INGENUITY SYSTEMS

Renal Necrosis/Cell Death	1.68E-02 - 1.30E-01	2
Glomerular Injury	4.96E-02 - 1.10E-01	1
Kidney Failure	1.10E-01 - 1.10E-01	1
Renal Fibrosis	1.10E-01 - 1.10E-01	1

(c) 2000-2014 Ingenuity Systems, Inc. All rights reserved.

Analysis Name: 50-50 Rb_h vs Sc_n - 2014-01-06 09:26 AM
Analysis Creation Date: 2014-01-06
Build version: 261899
Content version: 18030641 (Release Date: 2013-12-06)

Analysis settings

View
Reference set: Ingenuity Knowledge Base (Genes Only)
Relationship to include: Direct and Indirect
Includes Endogenous Chemicals
Optional Analyses: My Pathways My List

Filter Summary:
Consider only relationships where
confidence = Experimentally Observed
Cutoff:

Top Networks

ID	Associated Network Functions	Score
1	Cellular Movement, Hematological System Development and Function, Immune Cell Trafficking	27
2	Neurological Disease, Psychological Disorders, Cardiovascular Disease	24
3	Cardiovascular System Development and Function, Organismal Development, Skeletal and Muscular System Development and Function	24

(c) 2000-2014 Ingenuity Systems, Inc. All rights reserved.

4	Cardiovascular Disease, Developmental Disorder, Hereditary Disorder	23
5	Tissue Morphology, Organismal Survival, Cellular Compromise	23

(c) 2000-2014 Ingenuity Systems, Inc. All rights reserved.

Top Diseases and Bio Functions

Diseases and Disorders

Name	p-value	# Molecules
Nutritional Disease	1.84E-05 - 1.24E-02	13
Cancer	1.91E-04 - 1.26E-02	55
Infectious Disease	2.04E-04 - 1.09E-02	8
Inflammatory Response	2.04E-04 - 1.36E-02	17
Neurological Disease	3.37E-04 - 1.36E-02	30

Molecular and Cellular Functions

Name	p-value	# Molecules
Cell Morphology	8.43E-05 - 1.05E-02	27
Cellular Movement	8.55E-05 - 1.36E-02	24
Cell Death and Survival	8.57E-05 - 1.36E-02	12
Cellular Development	3.05E-04 - 1.18E-02	32
Cellular Growth and Proliferation	4.14E-04 - 1.18E-02	32

Physiological System Development and Function

Name	p-value	# Molecules
Nervous System Development and Function	1.16E-05 - 1.36E-02	21
Tissue Morphology	1.16E-05 - 1.11E-02	24
Organismal Development	1.58E-05 - 1.36E-02	32
Digestive System Development and Function	1.59E-05 - 1.81E-03	13
Organ Morphology	1.93E-05 - 1.36E-02	23

(c) 2000-2014 Ingenuity Systems, Inc. All rights reserved.

INGENUITY SYSTEMS

Top Canonical Pathways

Name	p-value	Ratio
Gluconeogenesis I	4.9E-03	2/47 (0.043)
Glycolysis I	5.32E-03	2/41 (0.049)
Sulfate Activation for Sulfonation	9.11E-03	1/8 (0.125)
Circadian Rhythm Signaling	1.05E-02	2/38 (0.053)
Neuropathic Pain Signaling In Dorsal Horn Neurons	1.08E-02	3/109 (0.028)

Top Molecules

Fold Change up-regulated

Molecules	Exp. Value	Exp. Chart
HTR5A	↑256.399	
PLOD2	↑219.023	
SLC16A3	↑200.172	
ATP4A	↑157.926	
PLA2G4D	↑97.723	
NIM1	↑91.574	
CYP26A1	↑68.505	
CXCR4	↑62.557	
KISS1R	↑61.942	
ANGPTL4	↑53.166	

Fold Change down-regulated

(c) 2000-2014 Ingenuity Systems, Inc. All rights reserved.

Molecules	Exp. Value	Exp. Chart
RB1	↓-17.702	
SPIC	↓-8.550	
DSC1	↓-7.922	
KBTBD8	↓-7.829	
UGT2B4	↓-7.661	
PTPRQ	↓-7.213	
EOMES	↓-6.839	
SKIDA1	↓-6.276	
CLEC2A	↓-6.112	
TARP	↓-6.111	

Top Upstream Regulators

Upstream Regulator	p-value of overlap	Predicted Activation State
EPAS1	5.50E-14	Activated
HIF1A	1.19E-13	Activated
farnesylthiosalicylic acid	1.73E-09	Inhibited
NEDD9	3.47E-07	Activated
Ca2+	6.77E-06	

(c) 2000-2014 Ingenuity Systems, Inc. All rights reserved.

INGENUITY SYSTEMS

Top My Lists

Name	p-value	Ratio

Top My Pathways

Name	p-value	Ratio

Top Tox Lists

Name	p-value	Ratio
CAR/RXR Activation	7.72E-03	2/29 (0.069)
Increases Cardiac Dysfunction	1.24E-02	2/37 (0.054)
Cytochrome P450 Panel - Substrate is a Vitamin (Human)	2.71E-02	1/6 (0.167)
Cytochrome P450 Panel - Substrate is a Vitamin (Mouse)	3.15E-02	1/7 (0.143)
Cytochrome P450 Panel - Substrate is a Vitamin (Rat)	3.15E-02	1/7 (0.143)

(c) 2000-2014 Ingenuity Systems, Inc. All rights reserved.

INGENUITY SYSTEMS

Top Tox Functions

Assays: Clinical Chemistry and Hematology

Name	p-value	# Molecules
Increased Levels of LDH	4.04E-02 - 4.04E-02	1
Increased Levels of Potassium	6.64E-02 - 6.64E-02	1
Increased Levels of Creatinine	1.52E-01 - 1.52E-01	1
Increased Levels of Alkaline Phosphatase	2.71E-01 - 2.71E-01	1
Increased Levels of Red Blood Cells	3.62E-01 - 3.62E-01	1

Cardiotoxicity

Name	p-value	# Molecules
Cardiac Arteriopathy	2.46E-03 - 9.11E-03	6
Cardiac Fibrosis	4.09E-03 - 3.15E-02	2
Cardiac Dysfunction	4.48E-03 - 4.48E-03	2
Congenital Heart Anomaly	5.32E-03 - 2.01E-01	4
Cardiac Necrosis/Cell Death	7.21E-03 - 5.88E-01	3

Hepatotoxicity

Name	p-value	# Molecules
Liver Hematopoiesis	3.05E-04 - 3.05E-04	2
Liver Damage	4.57E-03 - 2.05E-01	2
Liver Necrosis/Cell Death	9.11E-03 - 1.25E-01	4
Biliary Hyperplasia	1.36E-02 - 4.04E-02	1
Liver Proliferation	3.40E-02 - 7.91E-02	3

(c) 2000-2014 Ingenuity Systems, Inc. All rights reserved.

INGENUITY® SYSTEMS

Nephrotoxicity

Name	p-value	# Molecules
Renal Proliferation	2.26E-02 - 1.84E-01	2
Renal Necrosis/Cell Death	3.15E-02 - 3.32E-01	2
Kidney Failure	6.21E-02 - 2.59E-01	2
Glomerular Injury	9.17E-02 - 1.97E-01	1
Renal Damage	9.58E-02 - 3.47E-01	2

(c) 2000-2014 Ingenuity Systems, Inc. All rights reserved.
